# Dynamical regulations on mobility and vaccinations for controlling COVID-19 spread

**DOI:** 10.1038/s41598-022-07371-5

**Published:** 2022-03-03

**Authors:** Mevan Rajakaruna, Harshana Rajakaruna, Rupika Rajakaruna

**Affiliations:** 1 Department of Mathematical and Computational Sciences, University of Toronto, Mississauga, ON L5L1C6 Canada; 2 Battelle Center for Mathematical Medicine, The Abigail Wexner Research Institute, Nationwide Children’s Hospital, Columbus, OH 43215 USA; 3 Department of Zoology, University of Peradeniya, Peradeniya, 20400 Sri Lanka

**Keywords:** Diseases, Infectious diseases

## Abstract

Using a system of time-dynamical equations, we investigate how daily mobility indices, such as the homestay percentage above the pre-COVID normal ($H\%$; or *H*-forcing), and the vaccinated percentage ($V_c\%$; or *V*-forcing) impact the net reproductive rate (*R*0) of COVID-19 in ten island nations as a prototype, and then, extending it to 124 countries worldwide. Our *H*- and *V*-forcing model of *R*0 can explain the new trends in 106 countries. The disease transmission can be controlled by forcing down $R0(H,V_c) < 1$ with an enforcement of continuous $H > 40\%$ in $93\%$ of countries with $0\%$ vaccinated plus recovered, $V_p$. The required critical $H\%$ decreases with increasing $V_p\%$, dropping it down to $20\%$ with $25\% V_p$, and further down to $8\%$ with $50\% V_p$. However, the regulations on $H\%$ are context-dependent and country-specific. Our model gives insights into forecasting and controlling the disease’s transmission behaviour when the effectiveness of the vaccines is a concern due to new variants, and/or there are delays in vaccination rollout programs.

## Introduction

When a novel virus emerges, the community mitigation strategies, especially those concerning population mobility, are the most readily available intervention to slow down the transmission^1^. Most countries implemented strict population mobility policies to suppress transmission of SARS-CoV-2 (COVID-19), and in some countries, a convincing reduction in case-incidence was observed at least temporarily^2,3^. Even though many vaccination programs are rolled out worldwide, the current rate of spread of COVID-19, as a new infection wave, with Delta and other variants, concerns whether the vaccines may not be effective against the evolving variants. The reproductive rate, *R*0, of the Delta variant is much greater (5.08) compared to its ancestral variant (2.79)^4,5^. Due to the high *R*0 associated with higher transmissibility, low vaccine coverage rates, and low vaccine effectiveness, the social measures will need to be strengthened to combat the ever emerging variants. A high *R*0 also means much higher vaccine coverage rates need to be achieved compared to the ancestral variants^5^.

Understanding the extent of population mobility and the ongoing vaccination programs of a nation is essential for tracking the trajectory of the national epidemic and assessing the effectiveness of continuing control measures. Many studies have analyzed the efforts in controlling people’s mobility to reduce the spread of the disease^6–9^. Those include studies of correlations of *R*0 with the mobility metrics developed by Google^10^. Those metrics are based on Google logins by people and location identifiers, and computed as proxies for people’s spatial density movement as percentage changes from the pre-COVID-19 scenarios. Nouvellet et al.^9^ have shown that a drop in *R*0, below the critical $R0=1$ requiring for disease extinction, correlates with the homestay $H\%$. Many other studies also show similar findings^6–8^. However, the combined effect of people’s mobility restraints with nations’ vaccination percentages has not yet been understood enough through dynamical process modeling of COVID-19 disease transmission.

Here we designed a simple study to predict the degree of population mobility restrictions needed to bring the *R*0 below one for countries, given the numbers of their fully vaccinated individuals. We used a system of time-dynamical equations, incorporating the effect of homestay percentages $(H\%)$ and cumulative number of individuals vaccinated $(V_c)$, fitting them with mobility data from Google^10^ and new case and death data from the University of Oxford^11^, to compute the net reproductive rate *R*0 in 124 nations. We modeled the data from ten island nations as a prototype to select the best alternative model-hypothesis among three nested models: (1) Model 1: $M_H:$ incorporating the homestay $H\%$ effect, (2) Model 2: $M_V$: incorporating the effect of percentage vaccinations, *V*_*c*_% and (3) Model 3: $M_{HV}$: the effect of the combination of both the $H\%$ and the $V_c\%$, on the disease dynamics.

## Results

### The general case of $H\%$ and $V_c\%$ impact on *R*0 across nations

Among the three alternative models fitted to the data of new cases, *C*(*t*), and deaths, *D*(*t*), from ten island nations as a prototype, Model 3 $(M_{HV})$ that combines the effects of both the homestay $(H\%)$ and the vaccinations $(V_c\%)$, fitted the best, in general, as per the Akaike information criterion (AIC) (Table 1). In nations that had homestay regulations as the primary method of controlling the spread, the $M_H$ Model fitted equally well or slightly better than the $M_{HV}$ model, explaining the variations and the trends in the new case and the death data. Similarly, in nations that had vaccinations as the primary method of controlling the spread, the $M_V$ model fitted to the data equally well or better than the $M_{HV}$ model. This suggested that the $M_{HV}$ model represented the effect of both the homestay and the vaccinations, controlled via nations’ respective government and other regulations, on COVID-19 disease spread, as a general all-inclusive hypothesis, in understanding the *R*0 dynamics.

**Table 1:** The projected percentages of homestay $H\%$ and vaccinations plus recovered, $V_p\%$, required to bring *R*0 below 1, for the ten island nations (the prototype).

Island	Model	Parameters and metrics					
		$H(R0<1)$ $@Vp\%=ap$	$Vp(R0<1)$ $@H\%=0$	df	NLL	AIC	Working rank
United Kingdom	$M_H$	−	−	4	1.47E3	2.95E3	3
	$M_V$	−	−	2	1.47E3	2.95E3	2
	$M_{HV}$	$>20$	$>68$	4	1.46E3	2.93E3	1
Taiwan	$M_H$	−	−	4	0.80E3	1.62E3	2
	$M_V$	−	−	2	0.94E3	1.88E3	3
	$M_{HV}$	$>10$	$>85$	4	0.80E3	1.62E3	1
Sri Lanka	$M_H$	−	−	4	1.29E3	2.59E3	3
	$M_V$	−	−	2	1.27E3	2.57E3	2
	$M_{HV}$	$>30$	$>60$	4	1.22E3	2.46E3	1
Philippines	$M_H$	−	−	4	1.13E3	2.26E3	1
	$M_V$	−	−	2	1.14E3	2.29E3	3
	$M_{HV}$	$>22$	$>85$	4	1.13E2	2.27E3	2
Japan	$M_H$	−	−	4	1.40E3	2.81E3	1
	$M_V$	−	−	2	1.40E3	2.81E3	3
	$M_{HV}$	−	$>50$	4	1.40E2	2.81E3	2
Ireland	$M_H$	−	−	4	0.81E3	1.63E3	1
	$M_V$	−	−	2	0.88E3	1.77E3	3
	$M_{HV}$	$>12$	$>60$	4	0.83E3	1.67E3	2
Indonesia	$M_H$	−	−	4	1.33E3	2.68E3	2
	$M_V$	−	−	2	1.35E3	2.71E3	3
	$M_{HV}$	$>18$	$>82$	4	1.33E3	2.67E3	1
Haiti	$M_H$	−	−	4	0.84E3	1.69E3	2
	$M_V$	−	−	2	0.86E3	1.72E3	3
	$M_{HV}$	−	$>50$	4	0.84E3	1.69E3	1
Dominican Republic	$M_H$	−	−	4	1.04E3	2.08E3	3
	$M_V$	−	−	2	0.99E3	1.99E3	2
	$M_{HV}$	−	$>35$	4	0.99E3	1.98E3	1
Australia	*M*_*H*_	$-$	−	4	0.82E3	1.64E3	1
	$M_V$	−	−	2	0.84E3	1.68E3	3
	$M_{HV}$	−	$>50$	4	0.84E3	1.68E3	2

The Akaike Information Criteria (AIC) values of the fitted alternative $M_{H}$, $M_{V}$ and $M_{HV}$ models, and the critical values of $H(R0=1)\%$ and $V_p(R0=1)\%$ computed based on the all-representative $M_{HV}$ model are given (see graphs in Supplement S1 for all nations). The coefficient $\nu$, a proxy for the vaccine effectiveness, was set at 0.8 in island estimations. Thus, the degrees of freedom df in both $M_{H}$ and $M_{HV}$ models become 4. NLL-Negative log likelihood of the model fits. Parameter values and their CI are given in the Supplement S2. The graphs of $H(R0=1)\%$ with respect to $V_p\%$, and *R*0 with respect to $H\%$ based on the $M_{HV}$ are given in the Supplement S1. The *ap* stands for ‘as at present’.

**Figure 1:**
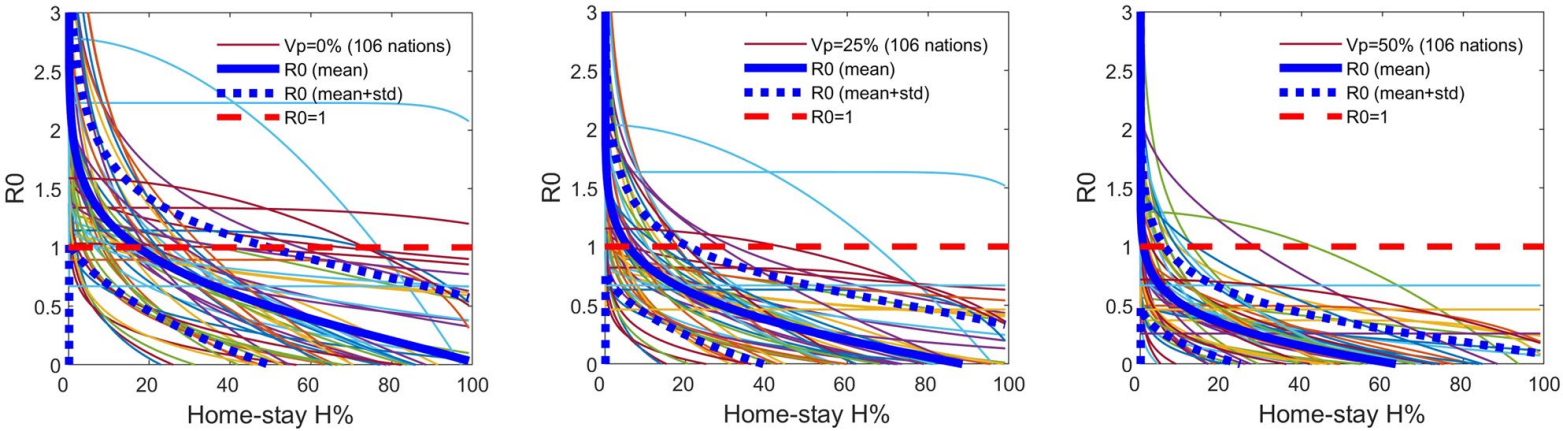
The World data -The net reproductive rate R0 vs. the percentages of Home-stay $H\%$ at different percentages of the population vaccinated plus recovered, $V_p\%$: The R0 decreases with the increasing $H\%$ at $V_p=0\%$, that is $0\%$ is vaccinated plus recovered from the susceptible (Left panel), $V_p=25\%$, (Middle panel) and $V_p=50\%$ (Right panel). Here, we plotted the 106 out of the 124 nations based on the estimated $M_{HV}$ model that explained the variation in the data of the respective nations. The 95 nations out of the 106 allowed enough variation in the degree of $H\%$ above the pre-COVID normal to make it possible to calibrate the *R*0 vs. $H\%$ functional relationship based on the model (Note that $V_p>=V_c$, where $V_c$ is the vaccinated population percentage). The functional relationship: $R0 = \gamma \Psi (1-\theta (H/100)^k)-\varepsilon$, where $\Psi$ is the susceptible population proportion, that is, the proportion of the total population *N* minus the effective number out of the vaccinated, $\nu V_c$, minus the number recovered, assuming $\nu$ as the likelihood that a vaccinated individuals not re-infected, or as a proxy for the average efficacy or the effectiveness of the vaccines. The $R0<1$ indicates the threshold below which there is a tendency for the disease going extinct. (see Supplement S1 for country-specific graphs).

We extended the $M_{HV}$ model-fitting and the analyses to the world data of 124 countries (see Supplements S1, S2). The model $M_{HV}$ could explain the variations and the trends in the data of 106 countries well w.r.t. the trends in their respective residual distributions. Ninety five out of the 106 countries showed a calibratable functional relationship between the *R*0 and the $H\%$ (Fig. 1), given their respective percentages of the people vaccinated plus the number recovered, denoted by $V_p\%$. Note that $V_p\% \ge$ the percentage vaccinated, $V_c\%$. The eleven out of the 106 countries did not show a marked variation in the *R*0 with respect to an increase in the $H\%$ because the variation in the $H\%$ data was not sufficient to capture such changes in the functional response of *R*0. That is, for those nations, the functional response of *R*0 with respect to $H\%$ , other than the values of *R*0 for their given $V_p\%$, was non-calibratable.

**Figure 2:**
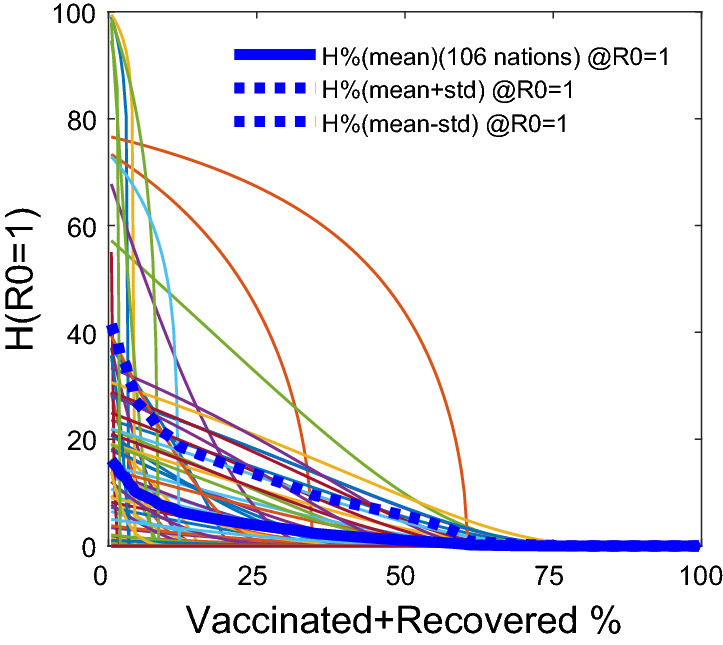
The World data- Homestay $H\%$ percentages vs. the vaccinated plus recovered population percentages: The homestay $H\%$, required at $R0=1$, given by the estimated $M_{HV}$ model, declines with the increase in the percentage $V_p\%$, that is, the percentage vaccinated plus the recovered in the populations. The functional relationship: $H(R0=1)=[(1/\theta )(1-(1/(\gamma \Psi ))(1+\varepsilon ))]^{(1/k)}$, where $\Psi =(1-V_p\%/100)$. The graph is drawn based on the $M_{HV}$ model that explained the variations in the data in 106 out of the 124 nations.

When the $H\%$ is increased, the *R*0 decreased for nations depending on the percentages of individuals vaccinated plus recovered, $V_p$: 0%, 25%, and 50%, in the said 106 countries (Fig. 1). The model $M_{HV}$ indicated that 93% of the nations, with both mobility regulations and effective vaccination programs, out of the 106, requires a minimum of 40% homestay above the pre-COVID normal to bring the $R0 < 1$ from the status quo (Fig. 1). This threshold of $H\%$ at $R0=1$ lowers with the increasing percentage of vaccinations. For example, with $> 25\%$ more vaccinated ($V_c\%$) plus the recovered, the $H\%$ at $R0=1$ lowers down to 20% in 95% of the 106 countries. For countries with $>50\% V_p$ , the $H\%$ requiring for $R0<1$ turns out to be about eight for about $92\%$ of the 106 nations (Fig. 1). Figure 2. shows how $H\%$ at $R=1$ decreases with the increasing $V_p\%$, i.e., the vaccinated plus recovered percentage. Overall, the *R*0, averaged over the last seven days, is negatively correlated with the percentage-vaccinated in the nations, with $R^2=0.41$ and $p<0.01$ (Fig. 65 in the Supplement S1). It indicates that $>80\%$ of fully vaccinated individuals in a nation does not fully guarantee a $R0<1$, with upper $95\%$ confidence interval crossing $R0=1$ is at $70\%$ fully vaccinated. The detailed country-specific plots, parameter values and their confidence intervals are given in the Supplements S1 and S2.

### Model-selection based on the island data -the prototype

**Figure 3:**
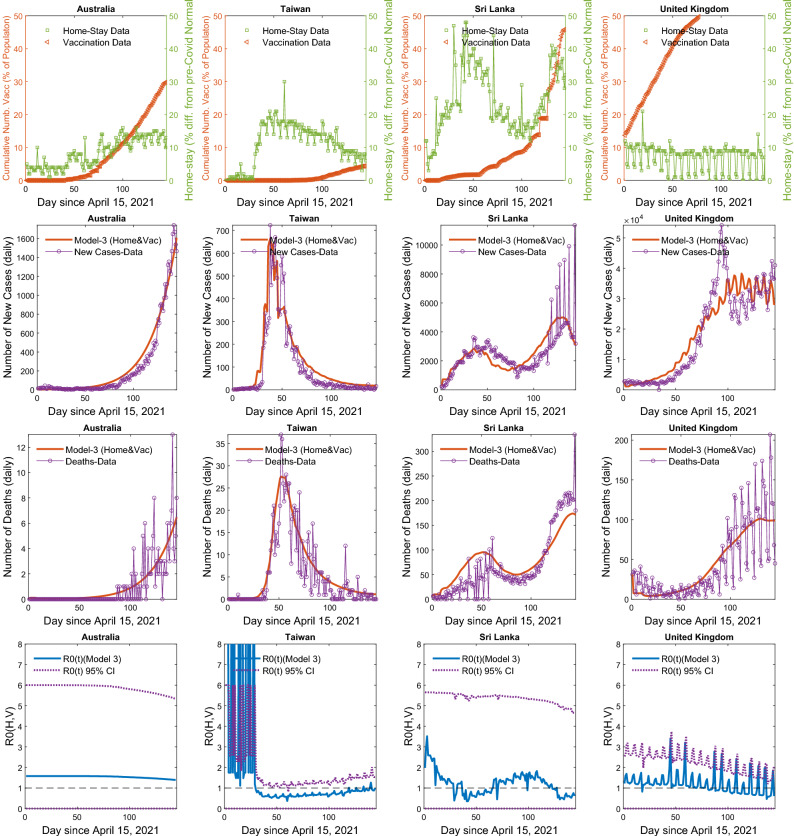
The $M_{HV}$ model hypothesis, incorporating forcing by homestay $H\%$ and percentage vaccinations $V_c\%$ on disease spread dynamics, fitted to the data in four regulatory-wise contrasting island nations: Top row panel: Daily homestay $H(t)\%$ and the percentage vaccinations $V_c(t)\%$ over time: Australia: No major *V*-forcing nor *H*-forcing: Taiwan: No major *V*-forcing but high *H*-forcing, Sri Lanka: Major increase in both *V*-forcing and *H*-forcing, United Kingdom: Major increase in *V*-forcing and no *H*-forcing. Second and third row panels: The model $M_{HV}$ fitted to new case, *C*(*t*), and death, *D*(*t*), data, and the resulting net reproductive rate, $R0(H(t),V_c(t))$, over time, given in the Bottom row panel. The $R0<1$ indicates a tendency towards decease-extinction. The values of the model selection criterion AIC are given in Table 1. The model fitted to all 124 countries are given in the Supplement S1, with parameter values and their CI’s given in the Supplement S2.

**Figure 4:**
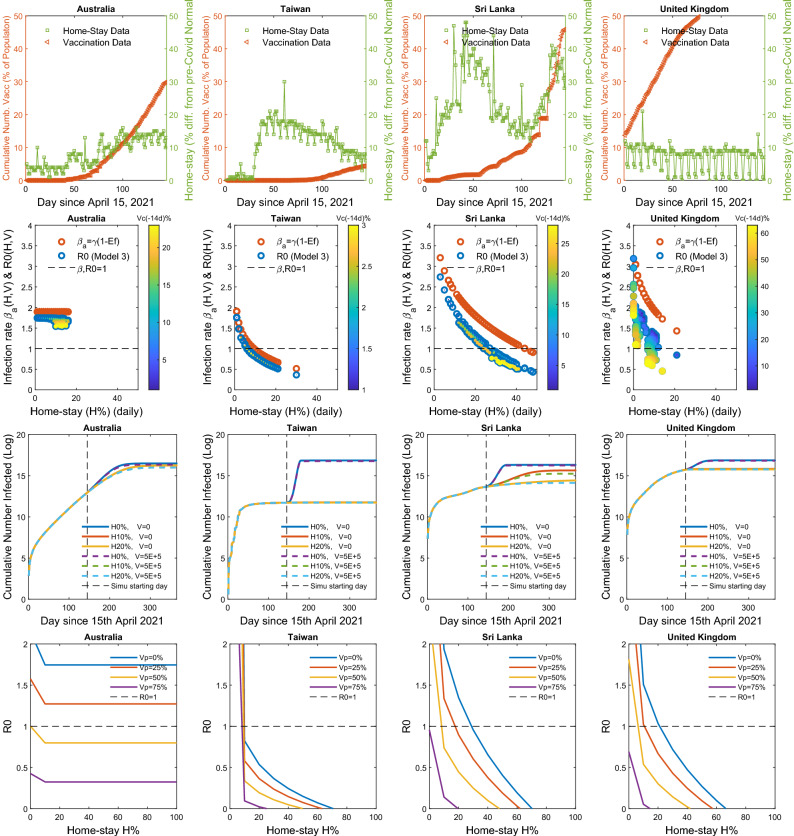
Functional responses of R0 vs. $H\%$ and $V_c\%$ for management forecasting: *Top row panel* The homestay percentage $H(t)\%$ and the percentage vaccinations $V_c(t)\%$ in four contrasting island nations: Australia: showing No major *V*-forcing nor *H*-forcing: Taiwan: No major *V*-forcing but high *H*-forcing, Sri Lanka: Major increase in both *V*-forcing and *H*-forcing, United Kingdom Major increase in *V*-forcing and no *H*-forcing. *Second row panel* Functional relationships between the infection rate $\beta$ vs. $H\%$, and the net reproductive rate *R*0 vs. $H\%$. The concave-up or-down relation is determined by the parameter *k*, depending on if $k<>1$ in the *H*-forcing function, which is $\beta =\gamma (1-\theta (H/100)^k)$. The $k=1$ yields the linear relationship. The curve may turn up or down depending on the quality and the strictness of the mobility controls. The $V_c\%$ pulls the $R0(H)\%)$ curve down forcing it towards $R0=1$ or lower. Here, the effect $E_f=\theta (H/100)^k$, s.t. $\beta =\gamma (1-E_f)$. Third row panel: Simulation forecasts based on the calibrated $M_{HV}$ model indicate how many more get infected from the status quo (i.e., as of today) for a choice of management scenarios of daily $H(t)\%$ and *V*(*t*) administered or done none. *Bottom row panel* The simulations further show how an increase in the percentage vaccinated plus recovered, $V_p\%$, forces the *R*0 to shift lower with respect to $H\%$. The graphs of all 124 countries are given in Supplement S1 with parameter estimates and their CI’s in Supplement S2.

The four nations presented in Figs. 3 and 4, among the ten island nations in the prototype analysis: Australia, Taiwan, Sri Lanka, and the UK, represent contrasting levels of $H\%$ vs $V_c\%$. Three of them, namely, Taiwan, Sri Lanka, and the UK showed a significant reduction in *R*0, except for the Australia. For Taiwan, the decline was due to an increased mobility restriction forcing a hike in $H\%$, but not due to vaccinations, while in the UK, it was due to escalating vaccinations but not by marked mobility restrictions (Fig. 3). In Sri Lanka, the control was driven by both the vaccinations and the mobility restrictions. The *R*0, in almost all islands in the analysis except for Australia, was less than one, indicating that their disease spread was under control. There was not enough variation in $H\%$ in the case of Australia to calibrate how *R*0 functionally responds to the changes in $H\%$. All four islands showed a concave-up functional relationships with respect to the increase in $H\%$. Sri Lanka shows the lowest sensitivity of *R*0 to the change in $H\%$, thus, needing a significant change of $H\%$ in reducing a unit of *R*0 compared to any other nation. The estimated values and AICs of the three alternative models $M_H$ , $M_V$ and $M_{HV}$ for the ten island nations are given in Table 1 (Supplement S2 gives estimated parameter values and their CI’s). The graphs of the model $M_{HV}$, fitted to new cases and deaths, plus other functional relationship of *R*0 vs. $H\%$ and $V_c\%$ are given in the Supplement S1 for all nations, with estimated parameter values given in Supplement S2.

The *R*0(*H*, *V*) vs. $H\%$ on the second row top panels in Fig. 4 shows the effect of the fully vaccinated percentages, $V_c(t)\%$, in pulling down the *R*0 curve stretched towards $R0=1$ or lower. The simulation results of the cumulative number of individuals infected vs time of the four island nations show the level of $H\%$ needed for $R0<1,$ indicating that the disease can be forced to extinct, i.e., $R0(H)<1$, from the status quo (i.e., on top of the already administered $V_c\%$ of the respective countries plus recovered) with an enforcement of a continuous homestay of $H\%>20$ from the pre-Covid normal. This is the same case for many other nations among the analyzed 106 nations given in the Supplement S1. The *R*0 vs. $H\%$ in the bottom panels in Fig. 4 shows that the percentage of vaccination, $V_c\%$, brings the required critical $H\%$ to meet the $R0(H)=1$ threshold further down, yielding a complementary effect on the reduction in the *R*0 forced by $H\%$. The analyses of the 106 nations in the Supplement S1 also show similar trends that we discussed here on the four island nations.

Furthermore, the average infection fatality rate, IFR, estimated from the model across 106 nations was 0.49% [0.37%, 0.61%]. The country-specific estimates are given in the Supplement S3. The mean incubation period across nations was estimated at 4.26 [4.01, 4.52] days, on average, and the length of infectiousness was at 8.12 [7.82, 8.42] days, on average, with 95% confidence intervals. The start of the death-window was estimated at 6.86 [6.53, 7.19] days, on average, from the day of the infection identification as new cases, and the average time-length of the death window was estimated at 15.89 [15.22, 16.55] days, on average, with $95\%$ confidence intervals. The parameter of vaccination effectiveness $\nu$ after two doses, with the time-lag of 14 days, given the mixed variants in the populations, yielded 62% [28%, 67%] on average, while the identification probability of new cases from the infected individuals, $\varepsilon$, was estimated at 32% [30%, 35%] on average, with 95% confidence intervals. The country-specific parameter estimates are given in the Supplement S2.

## Discussion

There is escalating uncertainty regarding the complexity behind the building up of new infection waves of COVID-19, both among the vaccinated and the non-vaccinated nations, in the presence of the ever-evolving new variants. Studies are inconclusive as to how the recommended, and approved vaccinations, respond to the new variants. Hence, it is imperative that we explore it in depth using models as to how the disease transmission responds to regulations on mobility along with the on-going vaccination efforts.

We show how the net reproductive rate, R0, functionally relates to the effect of homestay ($H\%$) and the percentage of individuals fully vaccinated ($V_C\%$). The regulations on people’s mobility, confining them to stay at homes > 20% more than the pre-COVID scenario (except for > 40% for three countries), have a grip on flattening the infection curve, and controlling the spread markedly, at about 75% of the people remaining susceptible, and > 8% at about 50% of the population remaining susceptible. Here, the susceptibility is a function of the number vaccinated, infected and died as at present. Nouvellet et al.^4^ indicate that 73% or more mobility restrictions from the pre-COVID normal baseline is required to reduce the *R*0 less than 1, based on a model which has not regarded the effect of vaccinations, analyzing the data for 52 countries. We show how *V*-forcing initiatives complement the *H*-forcing initiatives, flattening the infection curve further down lowering the R0 when the effect of vaccinations are taken into consideration. Studying, which policies and regulations of mobility restrictions that correlate with the Google mobility indices, will be country-specific, and will need ground-truthing. It will be more informative to model the same dynamics spatially explicitly in countries such as Japan and other, where the regional data on new cases, deaths, and mobility are available.

A meta-analysis by Meyerowitz-Katz et al.^12^ has yielded infection mortality rate, IFR, an average of 0.68% [0.53%, 0.82%] with 95% CI. Similar results have been reported: a locality specific average of 0.48% [0.32%, 52%] estimated by Radon et al.^13^, and a range of 0.02% − 0.86% estimated by Ioannidis^14^ in a meta analysis. These results of IFR of COVID-19 are comparable with our estimates yielding 0.49% [0.37%, 0.61%] for the data across 106 nations with mixed variants. Moreover the mean incubation period of $4.26\, (\pm \, 1.33)$ days that our model estimated for the data from mixed variants across 106 nations is comparable with the studies done by Kang et al.^15^ and Wang et al.^16^ who estimated the same for the Delta variant at 4–5.8 days, and 4–6 days, respectively. Rai et al.^17^ have reported an estimation of 5.76 [5.18%, 6.30%] days for earlier variants. Garcia-Garcia et al.^18^ have estimated the probability distribution of the incubation period as a log-normal distribution yielding a mean of 5.6 days and a median of 5 days. Moreover, the length of infectiousness, $8.12\, (\pm \, 1.58)$ days estimated by our model for the mixed variant data across 106 nations, was comparable with Xiang et al.^19^, who reported 2–10.2 days for earlier variants. Our model yielded a mean time-length of $15.89 \,(\pm \, 3.48)$ days for the death-window, with the starting day of deaths of the window estimated at $6.86\, (\pm \, 1.75)$ days , on average, since the day of individuals were identified as new cases. The mean deaths, estimated at 14.5 days with a median 23.2 days since individuals showed illness, has been shown by Garcia-Garcia et al.^18^ based on a log-normal distribution. Furthermore, a recent study by Liu et al.^20,21^ has estimated the pooled vaccine effectiveness, after two doses, at 85% [80%, 91%] for the prevention of Alpha variant of SARS-CoV-2 infections, 75% [71%, 79%] for the Beta variant, 54% [35%, 74%] for the Gamma variant, and 74% [62%, 85%] for the Delta variant. Our model, given the data from mixed variants, estimated the vaccine effectiveness at $63\%\, (\pm \, 24\%)$ on average across the 106 nations. Bernal et al.’s^21^ have shown large confidence intervals 30–95% for the same for various variants. Overall, our estimations for the data with mixed variants are comparable with the existing estimation ranges in the literature given for various variants.

Eighteen countries, out of 124, showing miss-fits to the model, may indicate that either the vaccinations do not respond to the disease spread as expected due to new variants, or the data are not representative enough of the true situations. There are a dearth of complaints regarding the miss-appropriate handling of the data by authorities in countries for political gains. Hence, the data can be biased and sparsed as well, not consistently reported, being unsuitable to model the reality.

The Google mobility data^10^ that we used as a surrogate for quantifying people’s homestay percentages can be under-representative of the true mobility of the people. This is because, not all people are using mobile phones in underdeveloped countries unlike those in the developed world. There had been no ground-truthing done to calibrate the correspondence between the Google mobility data^10^ versus the true mobility of the people. Also, the heterogeneity of population density over space may have a large effect on the variability of the disease spread, which is not accounted explicitly in our model. Our model can be improved to understand the effect of mobility, localized lockdowns, and vaccinations, spatially explicitly, where the data are available. Furthermore, peoples’ moving in and out of the country, the fluxes, can violate our model assumptions of systems being closed. The demographic factors such as age, sex, ethnicity, and also peoples’ walks of lives can impact the predictability of the true dynamics. To incorporate such, one may need a structurally more advanced model, together with demographic data to parameterize such. Besides, to tease apart the detailed effects, one may need drastically varying temporal state variables. Furthermore, when modeling disease-outbreaks, considering a country-wide population as one statistical unit may underestimate the true infection rates in large countries at the beginning of the disease spreads. Analysis of localized spread may be more suitable to grasp a better picture at the initial stages. Hence, our model may predict better when the countries are smaller and the populations are homogeneously distributed, and the case occurrences are randomly distributed, spatially. Ours captures the average dynamics within and across nations, generalizing the effect of mobility and vaccinations on the disease spread, representing more of a phenomenological perspective in the disease dynamics. That is, our study may answer the general nature of the impact of mobility and vaccinations in full on disease spread within nations.

The effect of restrictions on quantitative and qualitative *H* are country specific, depending on the nature of people’s responses against the nature of regulations, such as semi vs. full lock-downs in local vs. country-wide scenarios, together with other provisions—these need to be further studied specific to the given countries and localities. However, our study shows how the net reproductive rate responds to *H*-forcing and *V*-forcing, giving concave-up vs concave-down functional responses, along with *V*-forcing, beginning to turn $R0 < 1$ in many nations. The apparent in-elasticity in *R*0 w.r.t. *H* in Sri Lanka may suggest that there may be mixed-effects in people’s adhering to regulations. In contrast, countries such as Taiwan are more elastic, thus showing the firmness and the strength in controlling the wave drastically. What regulations attribute to the elasticity in the degree of $H\%$ needs a country-specific analyses.

Our findings may be useful as a tool for decision-support in controlling the disease spread and deaths in nations, when the effect of vaccines on forcing down the transmission is either weak, given the new variants, or not administered, or less affordable to nations.

## Methods

We obtained COVID-19 related daily new cases *C*(*t*), deaths *D*(*t*) and vaccinations *V*(*t*) data of 127 countries from Oxford University COVID-19 database^11^ for which Google’s homestay ($H\%$) metrics were available (Note: *t* is time). The country specific homestay ($H\%$) proxy metrics from Google^10^ is a surrogate for the percentage increase in people’s homestay with respect to their pre-COVID normal. The effect of various mobility restrictions enforced by governments are reflected upon their respective Google’s homestay metric $H\%$ (see Adams et al.^6^; Hakim et al.^7^). Our objective was to model the effect of $H(t)\%$ and the number of individuals fully vaccinated over time, $V_c(t)$, that is, with two doses for most vaccines, in a country, on the disease’s spread, in terms of the net reproductive rate metric *R*0(*t*) (Lui et al.^19^).

Taking Sri Lanka as a reference, we modeled the data from April 15, 2021 as a starting point of a new infection wave as a new surge of deaths began around that time. We used a system of discrete time-dynamical equations to model the decease transmission processes over time, incorporating the effects of $H(t)\%$ on decreasing the infection rate $\beta$, and *V*(*t*) on decreasing the number of susceptible *S*(*t*) in the population over time. This allowed us to compute the net reproductive rate R0(t) of the disease in each country, and analyze how it responds to various levels of $H(t)\%$ and the number of individuals in the country fully vaccinated $V_c(t)$ by time t, to support in management decision making (Note that our objective here is not to model the effect of government specific regulations and restrictions on $H(t)\%$. It may require detailed country specific studies).

Firstly, we took ten island nations (listed in Table 1) greater than 25,000 to 8.0E6 $\hbox {km}^2$ in land area, ranging from Haiti to Australia with countries’ populations ranging from 10 to 270 million to test our prototype disease dynamical model. We tested three alternative nested model hypotheses: (1) $M_{H}$ incorporating only the effect of $H\%$, (2) $M_V$ incorporating only the effect of $V_c\%$, i.e., the percentage vaccinated, and (3) $M_{HV}$ incorporating the effects of both the $H\%$ and the $V_c\%$. We used the all-representative best-fitted model for the general analyses of the disease dynamics across all 127 nations using Akaike information criteria.

### Modeling R0 as a function of *H*(*t*) and $V_c(t)$

We take *S*(*t*) as the number of susceptible individuals to the disease in a total population of, *N*, and *I*(*t*) as the number of newly infected individuals on day *t*. Per the mass-action law, we write the number of daily new infections as a linear function of *S*(*t*), normalized with respect to *N*, with a time-integral of *I*(*t*) of the infected individuals who are not quarantined or hospitalized, hence available for spreading the disease during the past period from $t=(t-t_{Is})$ to $t=(t-t_{Ie})$ days through which the infection is transferable to others after staying an incubation period of $t_{Ie}$ days by each. Thus, we write the number of new infections on day *t* as $\beta (S(t)/N)I_r(t)$, where $\beta$ is the infection rate per day, and $I_r(t)$ is an integral given by $I_r=\int _{t=t-t_{Ie}}^{t=t-t_{Is}}I(t)$ normalized dividing by the infectious period $(t_{Ie}-t_{Is})$.

Rai et al.^17^ reported the mean incubation period of COVID-19 at 5.67 [5.12, 6.30] days with 95% CI, during early-mid 2021, whereas a study by Elias et al.^22^ indicated a higher value of 6.38 days. The Delta variant has an incubation period of 4–5.8 (Kang et al.^15^) and 4–6 days (Wang et al.^16^). Garcia-Garcia et al.^18^ have estimated the probability distribution of the incubation period suggesting a log-normal distribution with mean 5.6 and median 5 days. We assume that the incubation periods in our data come from a mixture distributions due to various variants, including Delta, dominating in various countries. Hence, to minimize our uncertainties in variant mixtures in different populations, we estimated the infectiousness period $(t_{Is}-t_{Ie})$, with the incubation period given by $t_{Is}$, as parameters, from the data for each country using the full dynamical model. In doing so, we make a step-functional approximation to the infectious-window showing a hump-shaped curve (see illustration in Fig. 1a in Supplement S4), suggested by the proportion of individuals who are infectious today from those who were infected *t* days ago. A review on epidemic models of COVID-19 by Xiang et al.^19^ has suggested that the infectious period persists for 2.3–10 days on average. Thus, we used initial values ranging [3–6] days, on average, for the incubation period, and values ranging [6–10] days, on average, for the infectious period for a country, for estimating the infectious-window parameters. Furthermore, we assumed that the recovered individuals never get re-infected during the modeling time, which is 144 days since the beginning of the new COVID wave. Thus, at the beginning of the process, we can write $S(t=0)=N-\int _{-\inf }^{t=0-t_{Ie}}[I(t)+C(t)]-\int _{-\inf }^{t=0}D(t)$ for the non-vaccinated scenario, where *D*(*t*) are the number of deaths on day *t*, and *C*(*t*) are those quarantined or hospitalized after being identified as infected on day *t*. As the total number of individuals already infected is unknowable at the beginning of model-fitting for the computation of the remaining susceptible in the populations, we calibrated it as a parameter.

For the vaccinated scenario, we deduct $S(t=0)$ by the cumulative number of individuals fully vaccinated, $V_c$, up to $t_v$ days backwards in time from day $t=0$, assuming that it takes about $t_v=14$ days for an individual to be fully immune after receiving the full dose (two doses in most vaccines) as per the recommendations, thus, writing the the likelihood of those vaccinated being non-reinfecting, or removed from the susceptible as, $\nu V(t-t_v)$, with $\nu$ being the vaccine effectiveness, s.t., $0<\nu <1$. Note that thus $V_c$ data, accounted at time *t*, are those only fully vaccinated 14 days before the day *t*, considering their immunity was coming to full effect only after a two week time-interval). Shapiro et al.^23^ have indicated that the efficacy of COVID-19 vaccines against the known variants was at 84% on average, and while those vaccinated get infected, their likelihood of transmission of the disease to others estimated at 54%. We assume $\nu$ to be a conservative, fixed at 0.8 with fully vaccinated, in initial prototypical model-parameterization of the ten nations for model-comparison, and between 0.4 and 0.9 as bounds when parameterizing the models for all nations. This wide range allows lowering the effectiveness of the vaccine in scenarios such as in the emergent of new variants.

We consider the observed, or the identified new cases, *C*(*t*), are immediately self-quarantined or hospitalized in the case of COVID-19, such that, they are isolated from being able to infect other individuals in the community, that is, being removed from the $I_r(t)$, not being considered for the mass-action effect in contributing to further disease spread. We take the probability of identification of the infected from the currently infectious individuals as $\varepsilon$, allowing its range to be between a conservative, 0.15 to 0.6 bounds, in parameter estimations. It is known that symptomatic percentage was 13–18%^24^ in COVID-19. Thus, we further allowed a provision for being some identified in random checking.

 Study by Garcia-Garcia et al.^18^ has indicated that the probability of death at time (day) since an individual showed illness is a log-normal probability distribution. We assumed that the probability of death at time (day) since an individual was identified as a new case has a similar distribution, and hence, a step-function distribution approximation to the death-window for simplicity as illustrated in Fig. 1b in Supplement S4. Thus, we model daily deaths *D*(*t*) as a fraction of those individuals identified and quarantined, or hospitalized cases, *C*(*t*), accumulated between the $t_{Ds}$ and $t_{De}$ time window into the past, such that, the daily deaths are give by $\mu _1 I_d$, s.t., $I_d=\int _{t=t-t_{De}}^{t=t-t_{Ds}}C(t)$ averaged over the interval $(t_{De}-t_{Ds})$, s.t., $0<\mu _1<1$. Here, we estimate the average death-period $(t_{De}-t_{Ds})$ since identification as new cases, including $t_{De}$, as parameters.

Contou et al.^25^ have reported that median survival time, of 73 out of 153 patients admitted to ICU’s being critically ill due to refractory respiratory failure, shock with multiorgan failure, cardiac death, and neurological death, was 14 days. Geetha et al.^26^ have shown that the average time to peak the severity of symptoms, since the symptoms began, was 7 days on average. Garcia-Garcia et al.’s^18^ estimations of the probability distribution of death since illness yielded a mean of 14.5 days and a median of 13.2 days. As our data come from different mixtures of variants from different countries, we estimated the parameters of death-window using the full model, with starting day at $t_{De}$ for the initial values ranging [5–9] days, with death-window time-period $(t_{De}-t_{Ds})$ for initial values ranging [12–20] days.

To directly estimate the infection fatality rate IFR’s, which is defined as the rate of the number of associated deaths per the total number of infections (Staerk et al.^27^) over time windows, presented as a percentage, from the model, we assumed that daily deaths *D*(*t*) are a function of the total infected population, that is, proportion to $I_d=\int _{t=t-t_{De}}^{t=t-t_{Ds}}[I(t)+C(t)]$ averaged over the interval $(t_{De}-t_{Ds})$, s.t., $0<\mu _2<1$. Thus, it yields $IFR=100 \mu _2 \%$.

Thus, a discrete time-dynamical community COVID-19 infection model can be written as

$$\begin{aligned} S(t+\Delta t)&=S(t)-\beta (S(t)/N) I_r(t)\Delta t -\nu V(t-t_v)\Delta t,\\ I(t+\Delta t)&=\beta (S(t)/N) I_r(t)\Delta t -\varepsilon I_r(t)\Delta t,\\ C(t+\Delta t)&=\varepsilon I_r(t) \Delta t,\\ D(t+\Delta t)&=\mu I_d(t) \Delta t, \end{aligned}$$

where, $\Delta t=1$ is 1 day in our study.

To model the effect of homestay $H(t)\%$ percentage on reducing the infection rate $\beta$, we take $\beta$ as, $\beta =\gamma (1- \theta (H(t)/100)^k)$. Note that this functional formulation allows $\beta$ to decrease as a concave-up or -down function with increasing $H\%$, capturing the effectiveness of mobility regulations on $H\%$ reflecting on the rate of infection. We assume that countries having firm restriction on people’s mobility may produce a concave-down relationships, vice versa. Furthermore, $k=1$ yields the linearity in the relationship, and $\theta =0$ yields the Null model, in which the $H\%$ has no impact on the dynamics yielding $\beta =\gamma$. Note that the effect of daily $H(t)\%$ must have an immediate impact on the disease transmissibility without a time-delay.

Thus, the three alternative model-hypotheses we considered are give as below:

- *Model 1* $(M_H):$ Given by setting $\nu =0$.
- *Model 2* $(M_V):$ Given by setting $\theta =0$.
- *Model 3* $(M_{HV}):$ The Full model.

From the above system of equations, we can write the number gets newly infected at day $t+\Delta t$ as $I(t+\Delta t)=\beta (S(t)/N) I_r(t) -\varepsilon I_r(t)$. Hence, it yields the proportion $I(t+\Delta t)/I_r(t)=(\beta \Psi (t)-\varepsilon )$, where $\Psi (t)= S(f(V_c(t-t_v))/N$ between 0 and 1, s.t., $V_c=\int _{t=-inf}^{t=t-t_v}V(t)$, i.e., the cumulative number of individuals vaccinated by time point $(t-t_v)$. Note that when the $\int I(t+\Delta t)/\int I_r(t)>1$ over an infectious window, the number infected in the community inclines, whereas, when it is $<1$, the number infected in the community declines. Incorporating the *H*-forcing, s.t., $\beta =\gamma (1-\theta (H(t)/100)^k)$, thus, we can write,

$$\begin{aligned} R0(H(t))=\gamma \Psi (t)(1-\theta (H(t)/100)^k)-\varepsilon. \end{aligned}$$

This is a metric of net reproductive rate R0 of the infection written as function of H- and V-forcing over time.

### Fitting alternative model-hypotheses to the data and running simulations

We fitted the discrete system of time-dynamical equations to daily *C*(*t*) and *D*(*t*) data using the Matlab non-linear least-squares optimization function $lsqcurvefit$ to estimate the model parameters. We used the $n lparci$ function to derive the confidence intervals of the estimations from the resulting residuals, together with the Jacobian. Furthermore, we computed the Arkaike information criteria (AIC), of which the smallest value suggests the best among the competing alternative model hypotheses, after penalizing for the degrees of freedom of the models in explaining the variations in the dynamical data. Using the model that best-explained the data, we computed the net reproductive rate *R*0(*H*(*t*), *V*(*t*)) and $\beta (H(t))$ and their functional relationships with *H*- and *V*-forcing for further analyses. In fitting the data to models, we did not smooth data, but let the models capture the moving averages by themselves.

We simulated forecast of the model dynamics based on the general $M_{HV}$ model for the following different scenario: $H(t)\% =$ [0, 10, 20], and $V(t) =$ [0, 5E+5] per day. It also allowed comparing the threshold values of $H(R0 = 1)$ which the mathematical theory suggested.

### Analysis of the World data from 124 countries

We fitted the generalized model $M_{HV}$ to the data from 124 countries (listed in the Supplement S2). We also (1) tracked *R*0(*t*) and $\beta (t)$ over time *t* with their confidence intervals, (2) computed the functional responses of *R*0 and $\beta$ w.r.t. $H\%$, (3) change of *R*0 w.r.t. vaccinated percentage $V_c\%$, and (4) *R*0 w.r.t. $H\%$ and $V_c\%$ simultaneously, for each individual nation. We summarize the functional relationships between the (1) current R0 (averaged over the last 7 days) and the percentage vaccinations $V_c\%$ for all nations, (2) $H(R0=1)$ vs. $V_p\%$, the proportion non-susceptible, and (3) *R*0 vs. $H\%$ for all nations. Here, the functional relationships used for the above were: $R0=\gamma \Psi (1-\theta (H/100)^k)-\varepsilon$, where $\Psi$ is the susceptible population proportion which is a function of $V_c\%$, and $H(R0=1)=[(1/\theta )(1-(1/(\gamma \Psi ))(1+\varepsilon ))]^{1/k}$.

## Data

The study was carried out following PRISMA 2020 guidelines and regulations. Publicly available data-sets used: (1) The Google mobility data^10^: https://www.google.com/covid19/mobility/. (2) Daily new cases and deaths data from Oxford University^11^: https://github.com/owid/covid-19-data/blob/master/public/data/README.md.

The copies of the COVID-19 new cases, deaths and vaccination data obtained from the Oxford University^11^ and the community mobility reports for the same from Google^10^, of the 127 nations, plus the Matlab program coding developed for model-fitting and data analyses needing to reproduce the results of all graphs, tables, and estimations are stored at the online repository https://github.com/SOMAburr/dynamical-regulations-on-mobility-and-vaccinations-for-controlling-COVID-19-spread.git.

## Supplementary Information

Supplementary Information.

Supplementary Information 1.

Supplementary Information 2.

Supplementary Information 3.

## References

[1] CDC (2020). Implementation of Mitigation Strategies for Communities with Local COVID-19 Transmission.

[2] Unwin HJ (2020). State-level tracking of COVID-19 in the United States. Nat. Commun..

[3] Vollmer MAC (2020). A sub-national analysis of the rate of transmission of COVID-19 in Italy. MedRxiv..

[4] Liu Y, Gayle AA, Wilder-Smith A, Rocklov J (2020). The reproductive number of COVID-19 is higher compared to SARS coronavirus. J. Travel Med..

[5] Liu Y, Rocklov J (2021). The reproductive number of the Delta variant of SARS-CoV-2 is far higher compared to the ancestral SARS-CoV-2 virus. J. Travel Med..

[6] Adams L, Adams RJ, Bastiampillai T (2020). Australia can use population level mobility data to fight COVID-19. Med. J. Aust..

[7] Hakim AJ (2021). Mitigation policies, community mobility, and COVID-19 case counts in Australia, Japan, Hong Kong, and Singapore. Public Health.

[8] Li Y (2021). The impact of policy measures on human mobility, COVID-19 cases, and mortality in the US: A spatiotemporal perspective. Int. J. Environ. Res. Public Health.

[9] Nouvellet P (2021). Reduction in mobility and COVID-19 transmission. Nat. Commun..

[10] *COVID-19 Community Mobility Reports*. https://www.google.com/COVID-19/mobility (Accessed 10 July 2021).

[11] Ritchie, H. *et al*. *Coronavirus Pandemic (COVID-19). Published Online at OurWorldIn-Data.org* (2020). https://ourworldindata.org/coronavirus (Accessed 10 July 2021).

[12] Meyerowitz-Katz G, Merone L (2020). A systematic review and meta-analysis of published research data on COVID-19 infection-fatality rates. Int. J. Infect. Dis..

[13] Radon K (2020). Protocol of a population-based prospective COVID-19 cohort study Munich, Germany (KoCo19). BMC Public Health.

[14] Ioannidis JPA (2021). Infection Fatality Rate of COVID-19 Inferred from Seroprevalence Data.

[15] Kang M (2021). Transmission dynamics and epidemiological characteristics of Delta variant infections in China. MedRxiv..

[16] Wang Y (2021). Transmission, viral kinetics and clinical characteristics of the emergent SARS-CoV-2 Delta VOC in Guangzhou, China. EClinicalMedicine.

[17] Rai B, Shukla A, Dwivedi LK (2021). Incubation period for COVID-19: A systematic review and meta-analysis. Z. Gesundh. Wiss..

[18] Garcıa-Garcıa D (2021). Retrospective methodology to estimate daily infections from deaths (REMEDID) in COVID-19: The Spain case study. Sci. Rep. U.K..

[19] Xiang Y (2021). COVID-19 epidemic prediction and the impact of public health interventions: A review of COVID-19 epidemic models. Infect. Dis. Model..

[20] Liu Q, Qin C, Liu M, Liu J (2021). Effectiveness and safety of SARS-CoV-2 vaccine in real-world studies: A systematic review and meta-analysis. Infect. Dis. Poverty.

[21] Bernal JL (2021). Effectiveness of COVID-19 vaccines against the B.1.617.2 (Delta) variant. N. Engl. J. Med..

[22] Elias C, Sekri A, Leblanc P, Cucherat M, Vanhems P (2021). The incubation period of COVID- 19: A meta-analysis. Int. J. Infect. Dis..

[23] Shapiro J (2021). Efficacy Estimates for various COVID-19 vaccines: What we know from the literature and reports. MedRxiv..

[24] Subramanian R, He Q, Pascual M (2021). Quantifying asymptomatic infection and transmission of COVID-19 in New York City using observed cases, serology, and testing capacity. Proc. Natl. Acad. Sci..

[25] Contou D (2021). Causes and timing of death in critically ill COVID-19 patients. Crit. Care.

[26] Geetha S, Narayanamoorthy S, Manirathinam T, Kang D (2021). Fuzzy case-based reasoning approach for finding COVID-19 patients priority in hospitals at source shortage period. Exp. Syst. Appl..

[27] Staerk C, Wistuba T, Mayr A (2021). Estimating effective infection fatality rates during the course of the COVID-19 pandemic in Germany. BMC Public Health.

