# Supplementary material for: Dynamical regulations on mobility and vaccinations for controlling COVID-19 spread
Source: Sci Rep. 2022 Mar 3;12:3554. doi: 10.1038/s41598-022-07371-5 (PMC8894369; doi:10.1038/s41598-022-07371-5)
Supplement: Supplementary file 1 — Supplementary Information. [file 41598_2022_7371_MOESM1_ESM.pdf]

Dynamical regulations on mobility and vaccinations for controlling COVID-19 spread

Model M_{HV} fitted to the World data of 124 nations

The following analyses contain Homestay $H\%$ data from Covid19 Community Mobility Reports[1]1 of 124 nations. The country-related data of new cases, deaths, and vaccinations were downloaded from the Oxford University Covid19 database[10]. Out of those 127 nations, three nations, namely, Barbados, Bekina Farco, and Benn, were excluded from the analyses due to lack of numbers in deaths and/or new cases to model the dynamics. All the data and the Matlab program code used for the analyses are made available in the Repository.

Pg. 2:65: The model MHV with H- and V -forcing could explain the trends and the variations in the data in 106 out of the 124 nations. The data of the following nations fitted to the model qualitatively poorly as per the trends in residuals: Bangladesh, Belarus, Bulgaria, Croatia, Egypt, Estonia, Kyrgiztan, Latvia, Moldova, Romania, Russia, Serbia, Slovakia, Solvania, Sweden, Tajikistan, Tonga, and Ukraine (18 nations).

Figures of each country: The M_{HV} model hypothesis, incorporating forcing by Homestay $H\%$ and cumulative number fully Vaccinated $V_c\%$ on disease spread dynamics, fitted to the data of 124 countries:

Page 1 of each country: The top panel: Daily homestay $H(t)\%$ and the percentage of cumulative number of individuals fully vaccinated $V_c(t)\%$ over time. Second and third row panels: The model M_{HV} fitted to new Cases, $C(t)$, and Death, $D(t)$, data. Fourth row panel: The resulting net reproductive rate, $R0(H(t), V_c(t))$, and the infection rate, $\beta(H(t))$, over time. Bottom row panel: How $R0(H(t), V_c(t))$ behaves w.r.t. $H\%$ together with the countries' $Vc\%$ given in color.

Page 2 of each country: The top panel: Daily homestay $H(t)\%$ and the percentage of cumulative number vaccinated $V_c(t)\%$ over time. Second row panel: How $R0(H(t), V_c(t))$ behaves

w.r.t. $V_c\%$ together with varied $H\%$ given in color. Third row panel: $R0(H, V) = 1$ shown with a red ring, given the $H\%$ vs $V_c\%$ of the country. Fourth row panel: Simulations done from the last day of the model fitting imposing varying levels of $H\%$ together with continued daily vaccinations V vs none administered. Bottom row panel: The resulting $R0$ w.r.t. $H\%$ for simulated $V_p\%$.

The estimated parameter values and their confidence intervals are given in Appendices A2.

Pg. 67: The World data: Current R0's vs. the vaccinated population percentages.

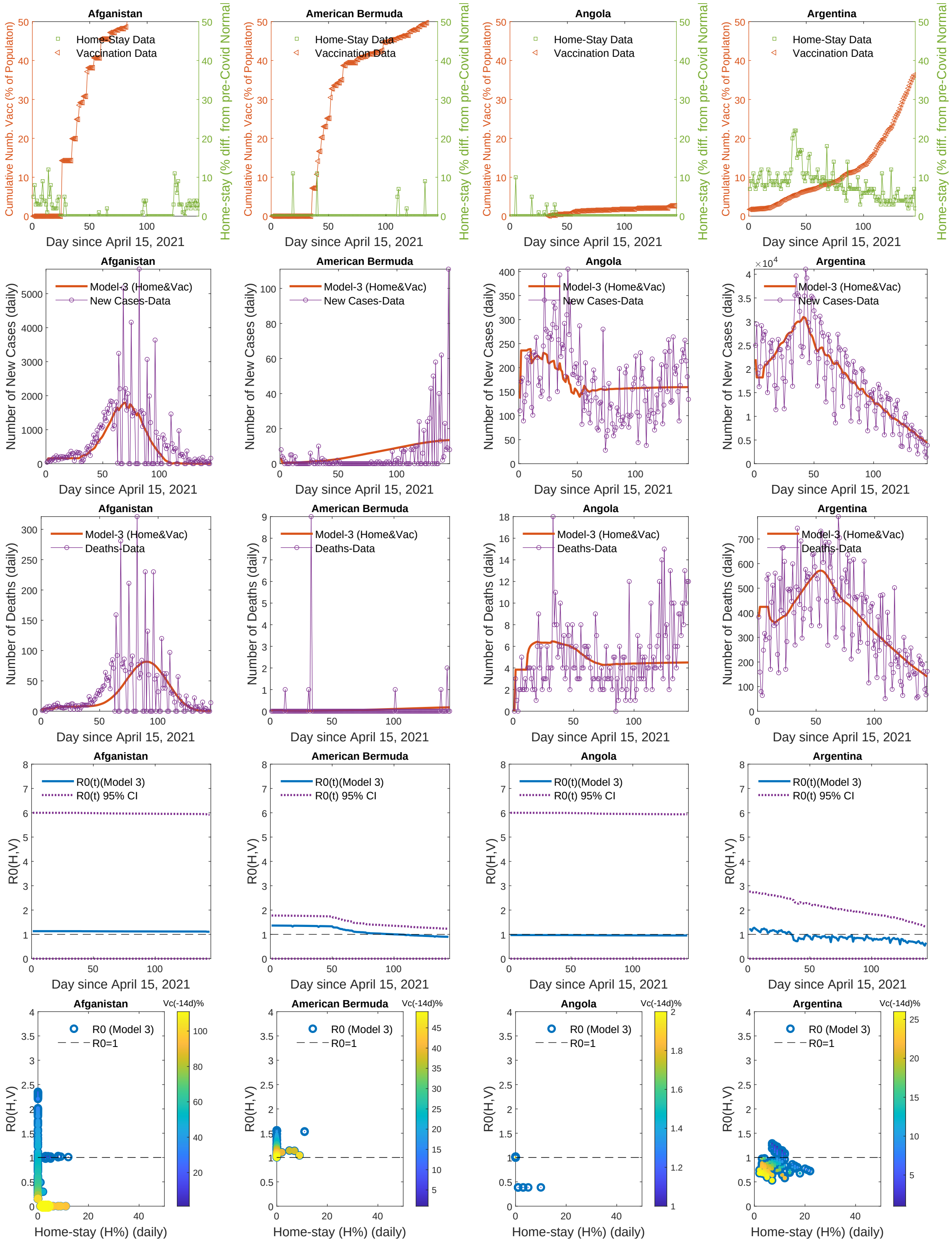

Afganistan
American Bermuda
Angola
Argentina
Home-Stay Data
Vaccination Data
Cumulative Numb. Vacc (% of Populaton)
Home-stay (% diff. from pre-Covid Normal
Day since April 15, 2021
Model-3 (Home&Vac)
New Cases-Data
Number of New Cases (daily)
Deaths-Data
Number of Deaths (daily)
R0(t)(Model 3)
R0(t) 95% CI
R0(H,V)
R0 (Model 3)
R0=1
Vc(-14d)%
Home-stay (H%) (daily)

Figure 1

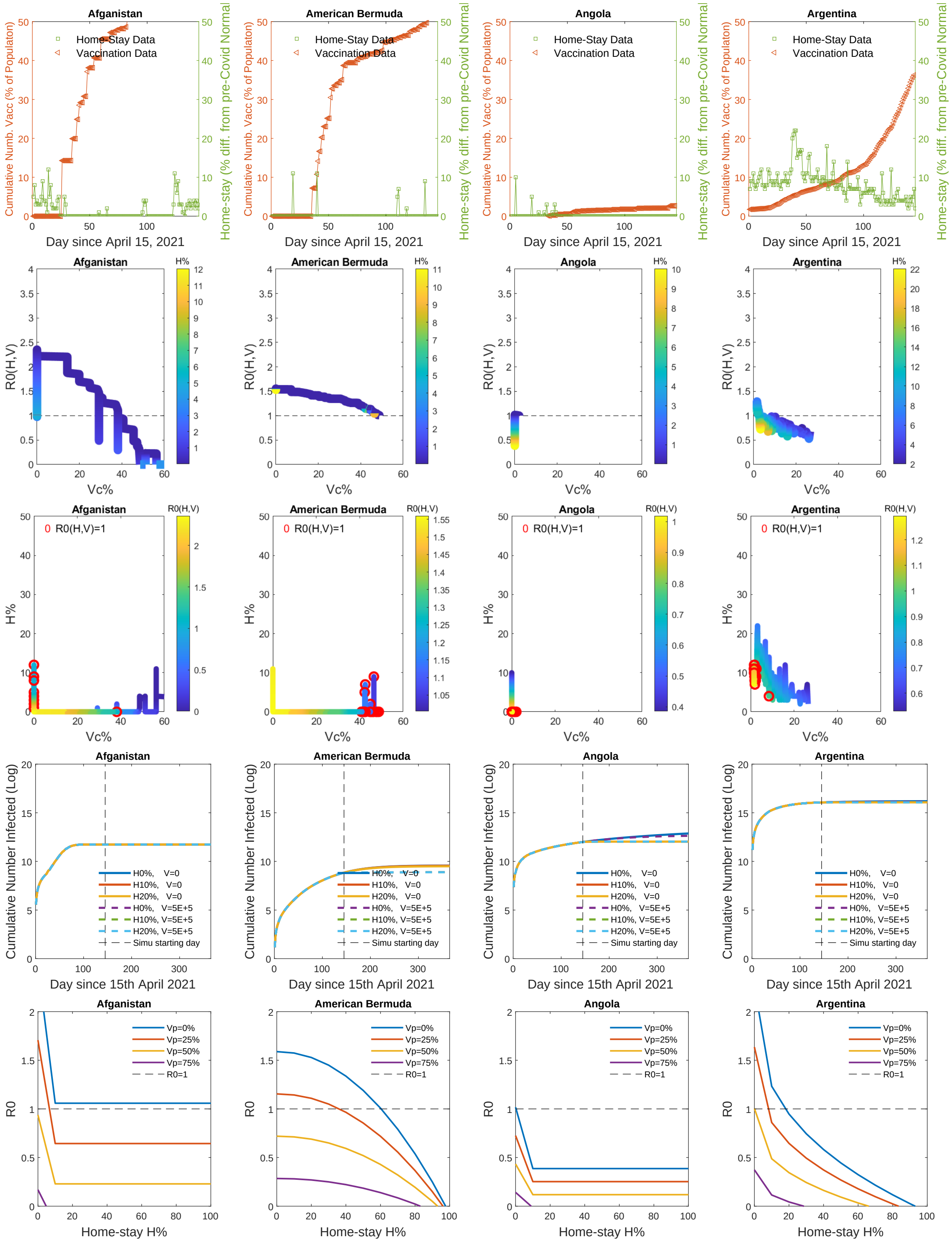
Afganistan
American Bermuda
Angola
Argentina
Home-Stay Data
Vaccination Data
Cumulative Numb. Vacc (% of Populaton)
Home-stay (% diff. from pre-Covid Normal
Day since April 15, 2021
R0(H,V)
H%
Vc%
R0(H,V)=1
Cumulative Number Infected (Log)
H0%, V=0
H10%, V=0
H20%, V=0
H0%, V=5E+5
H10%, V=5E+5
H20%, V=5E+5
Simu starting day
Day since 15th April 2021
R0
Vp=0%
Vp=25%
Vp=50%
Vp=75%
R0=1
Home-stay H%

Figure 2

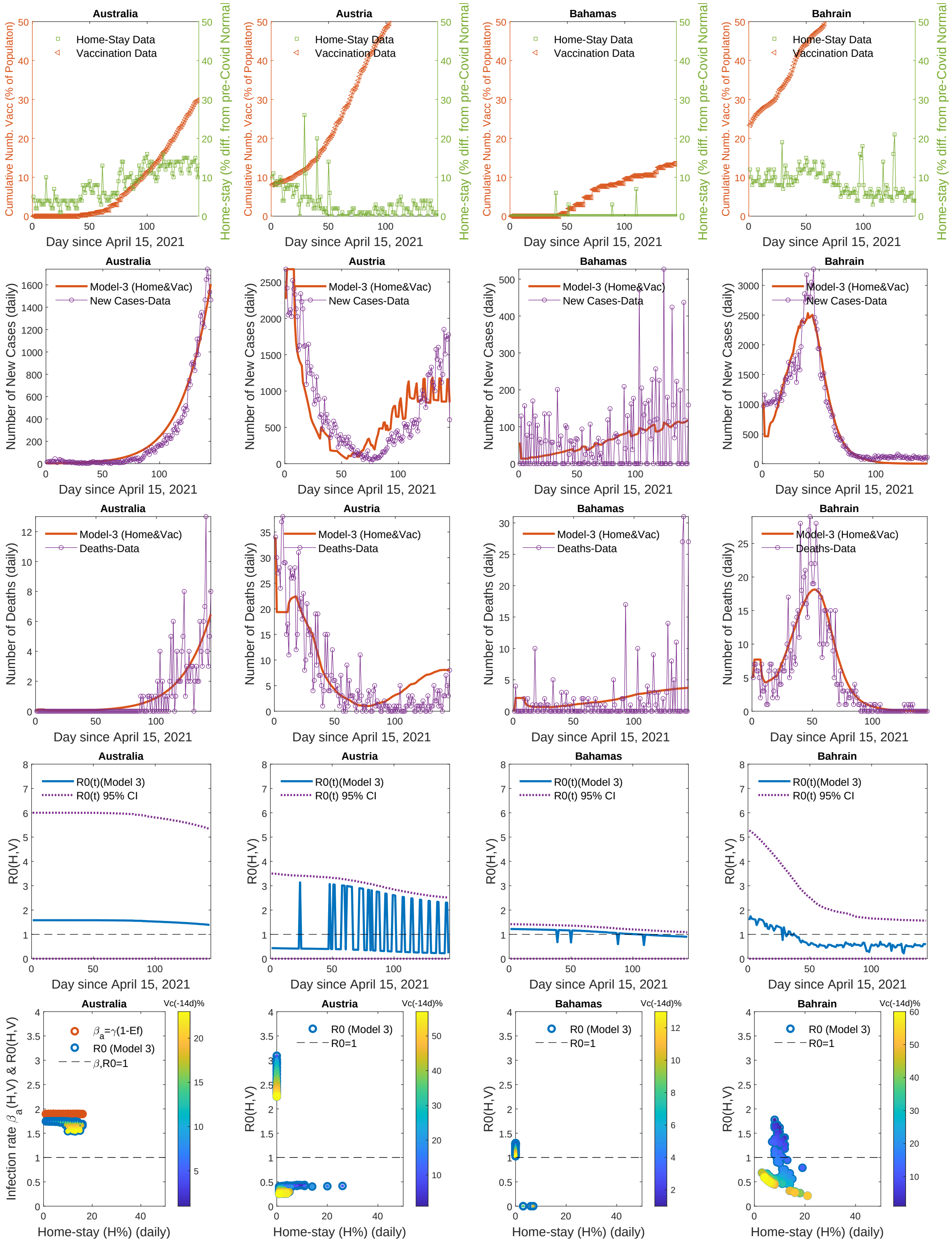

Australia
Austria
Bahamas
Bahrain
Home-Stay Data
Vaccination Data
Cumulative Numb. Vacc (% of Populaton)
Home-stay (% diff. from pre-Covid Normal
Day since April 15, 2021
Model-3 (Home&Vac)
New Cases-Data
Number of New Cases (daily)
Deaths-Data
Number of Deaths (daily)
R0(t)(Model 3)
R0(t) 95% CI
R0(H,V)
β_a=γ(1-Ef)
R0 (Model 3)
β,R0=1
R0=1
Infection rate β_a(H,V) & R0(H,V)
Vc(-14d)%
Home-stay (H%) (daily)

Figure 3

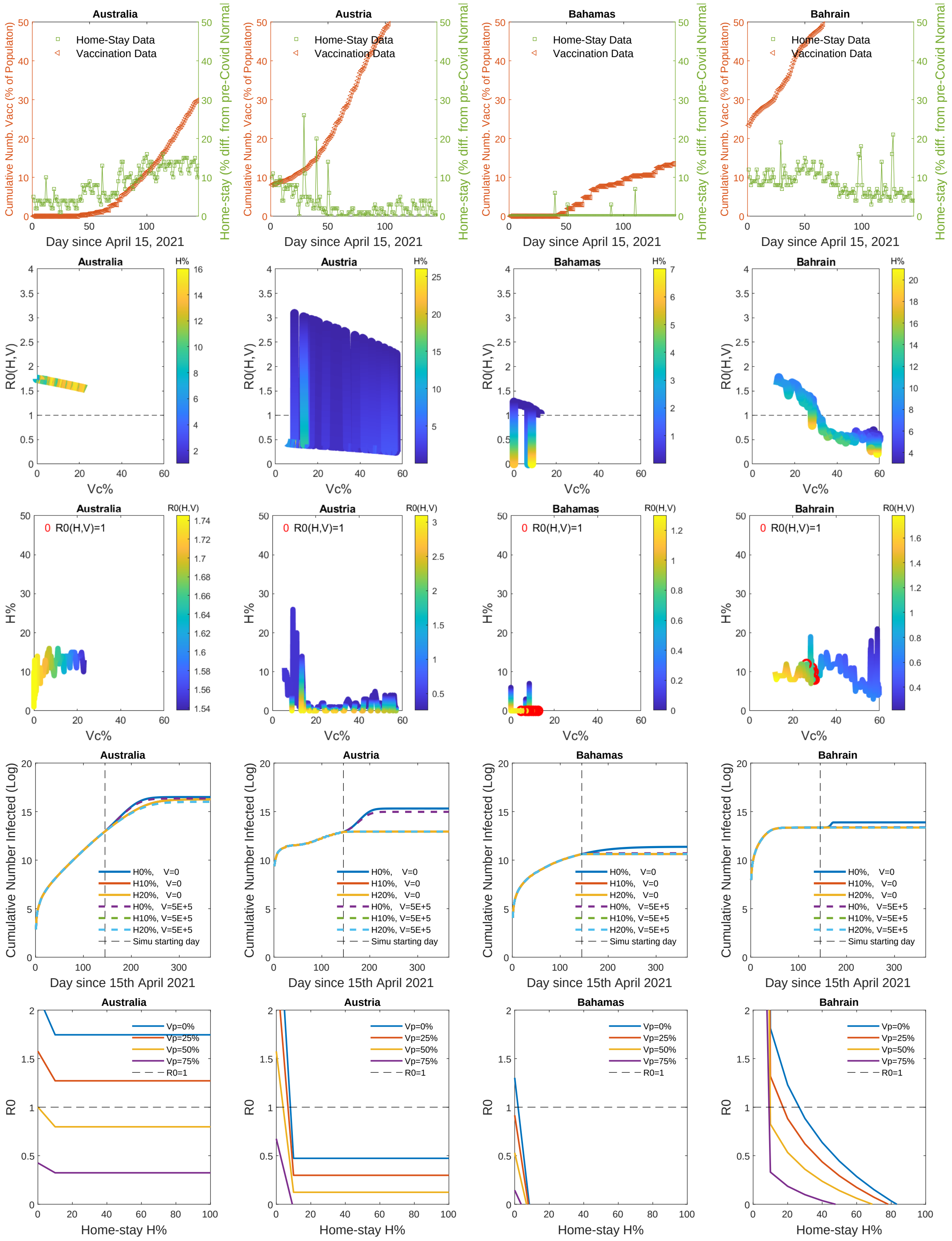
Australia
Austria
Bahamas
Bahrain
Home-Stay Data
Vaccination Data
Cumulative Numb. Vacc (% of Populaton)
Home-stay (% diff. from pre-Covid Normal
Day since April 15, 2021
R0(H,V)
H%
Vc%
0 R0(H,V)=1
Cumulative Number Infected (Log)
H0%, V=0
H10%, V=0
H20%, V=0
H0%, V=5E+5
H10%, V=5E+5
H20%, V=5E+5
Simu starting day
Day since 15th April 2021
R0
Vp=0%
Vp=25%
Vp=50%
Vp=75%
R0=1
Home-stay H%

Figure 4

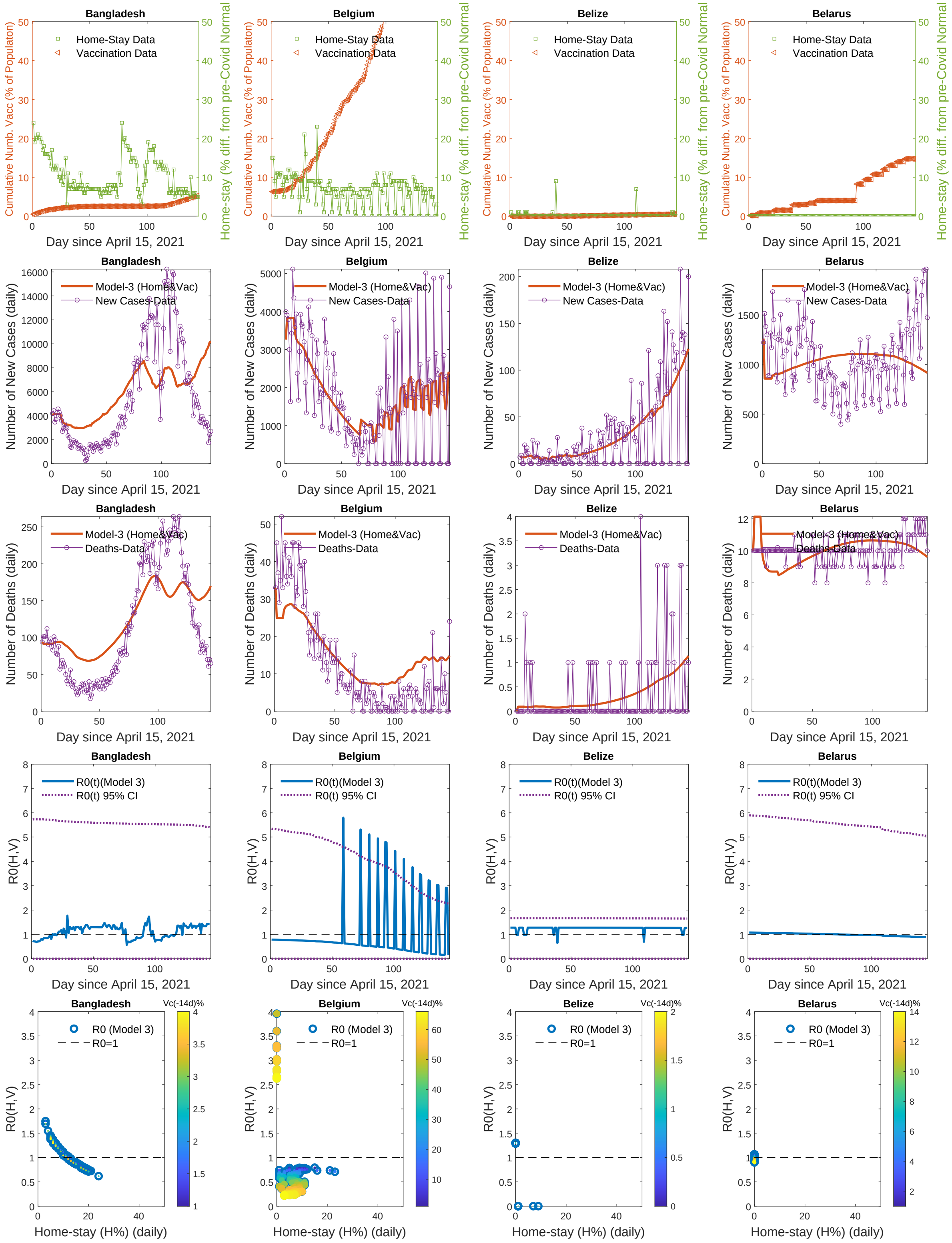

Bangladesh
Belgium
Belize
Belarus
Home-Stay Data
Vaccination Data
Cumulative Numb. Vacc (% of Populaton)
Home-stay (% diff. from pre-Covid Normal
Day since April 15, 2021
Model-3 (Home&Vac)
New Cases-Data
Number of New Cases (daily)
Deaths-Data
Number of Deaths (daily)
R0(t)(Model 3)
R0(t) 95% CI
R0(H,V)
R0 (Model 3)
R0=1
Vc(-14d)%
Home-stay (H%) (daily)

Figure 5

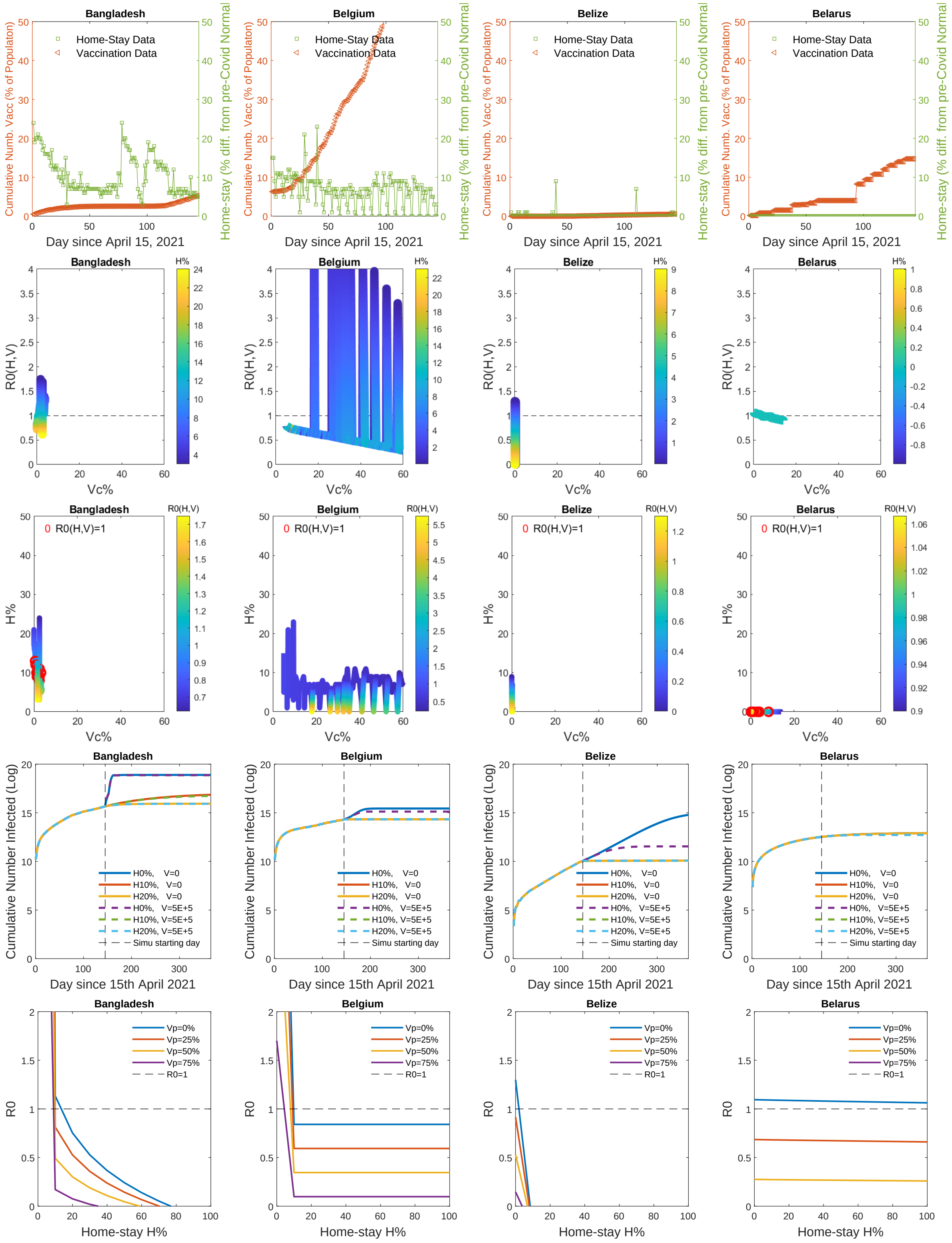
Bangladesh
Belgium
Belize
Belarus
Home-Stay Data
Vaccination Data
Cumulative Numb. Vacc (% of Populaton)
Home-stay (% diff. from pre-Covid Normal
Day since April 15, 2021
R0(H,V)
H%
Vc%
R0(H,V)=1
Cumulative Number Infected (Log)
H0%, V=0
H10%, V=0
H20%, V=0
H0%, V=5E+5
H10%, V=5E+5
H20%, V=5E+5
Simu starting day
Day since 15th April 2021
R0
Vp=0%
Vp=25%
Vp=50%
Vp=75%
R0=1
Home-stay H%

Figure 6

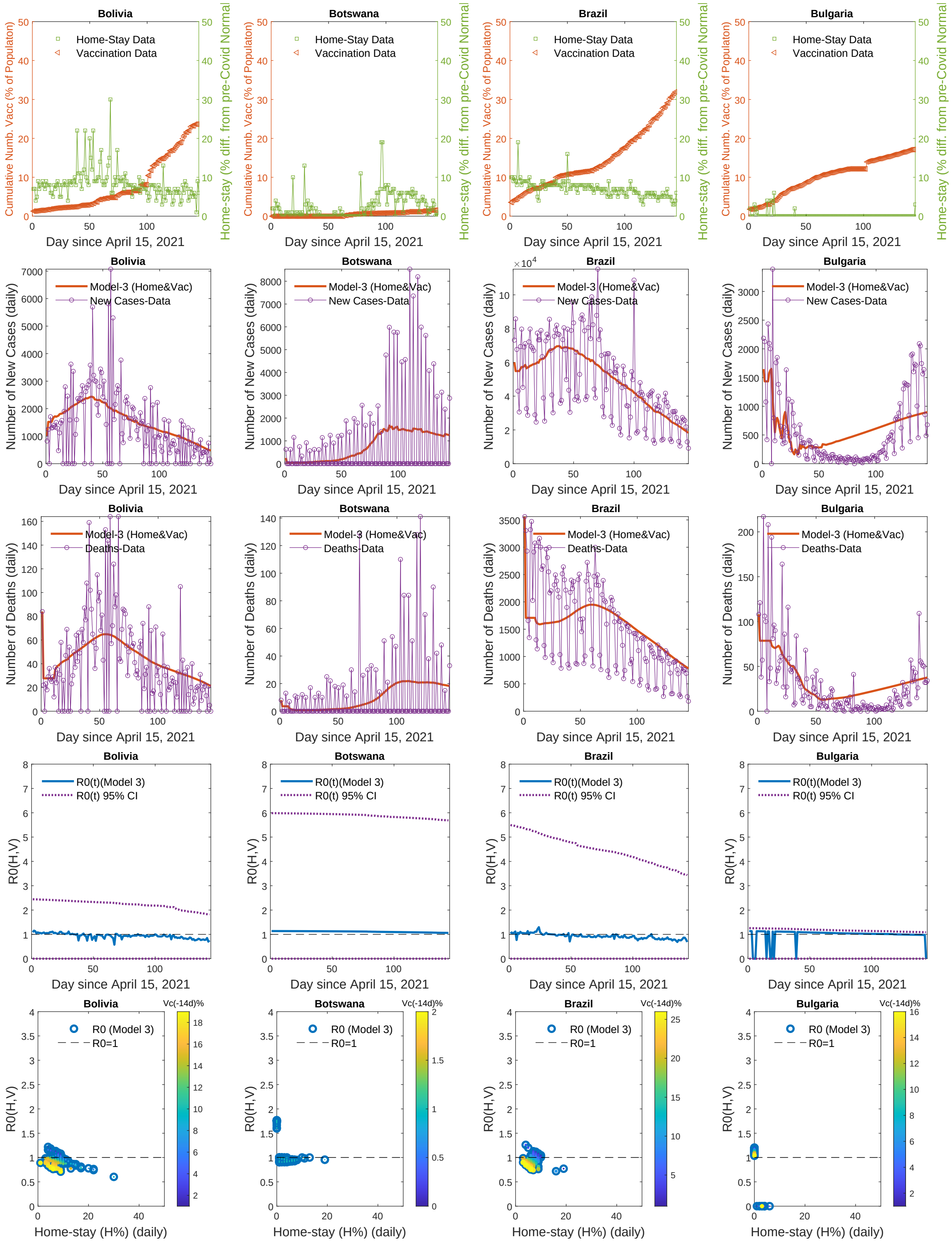
Bolivia
Botswana
Brazil
Bulgaria
Home-Stay Data
Vaccination Data
Cumulative Numb. Vacc (% of Populaton)
Home-stay (% diff. from pre-Covid Normal
Day since April 15, 2021
Model-3 (Home&Vac)
New Cases-Data
Number of New Cases (daily)
Deaths-Data
Number of Deaths (daily)
R0(t)(Model 3)
R0(t) 95% CI
R0(H,V)
R0 (Model 3)
R0=1
Vc(-14d)%
Home-stay (H%) (daily)

Figure 7

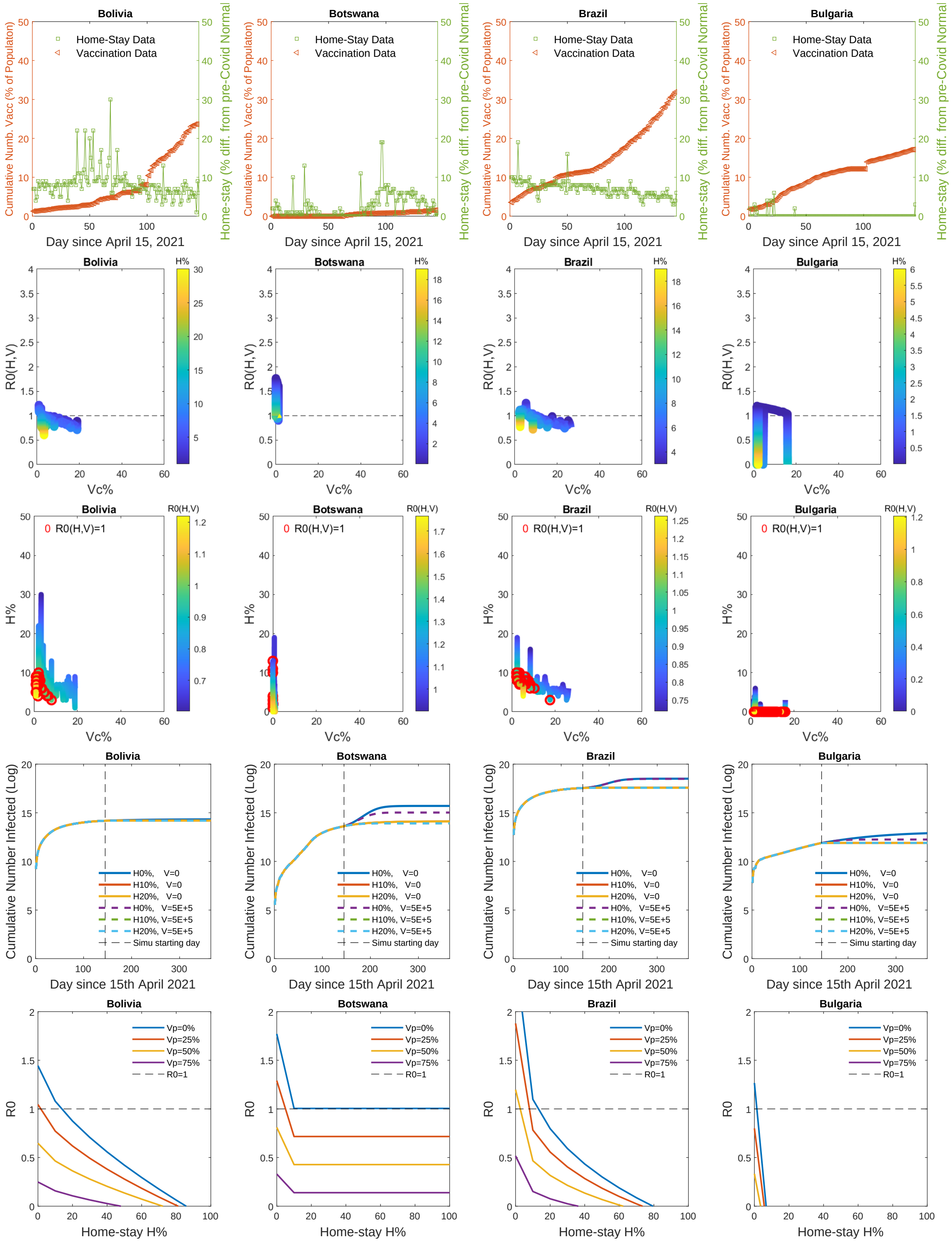
Bolivia
Botswana
Brazil
Bulgaria
Home-Stay Data
Vaccination Data
Cumulative Numb. Vacc (% of Populaton)
Home-stay (% diff. from pre-Covid Normal
Day since April 15, 2021
R0(H,V)
Vc%
H%
R0(H,V)=1
Cumulative Number Infected (Log)
H0%, V=0
H10%, V=0
H20%, V=0
H0%, V=5E+5
H10%, V=5E+5
H20%, V=5E+5
Simu starting day
Day since 15th April 2021
R0
Vp=0%
Vp=25%
Vp=50%
Vp=75%
R0=1
Home-stay H%

Figure 8

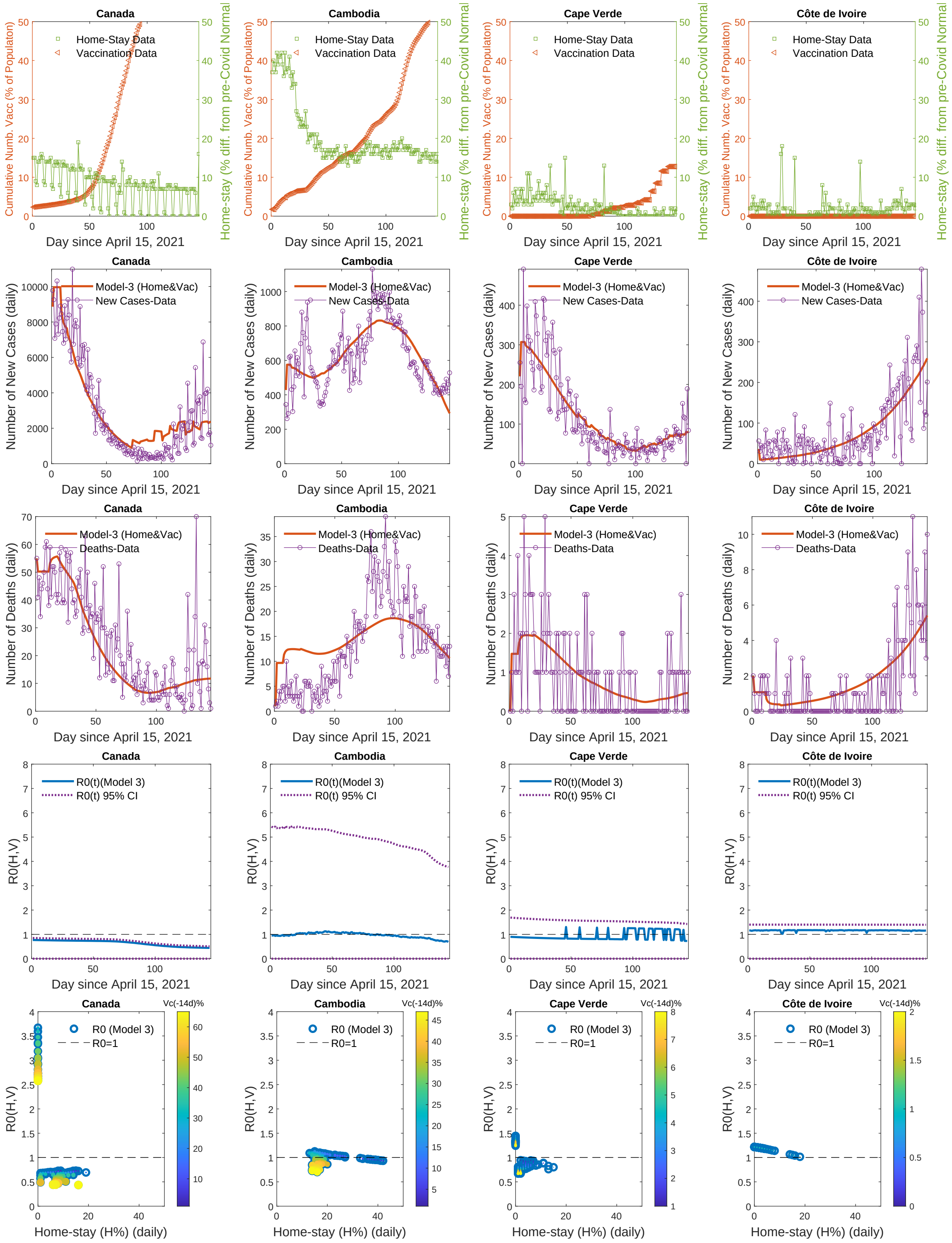
Canada
Cambodia
Cape Verde
Côte de Ivoire
Home-Stay Data
Vaccination Data
Cumulative Numb. Vacc (% of Populaton)
Home-stay (% diff. from pre-Covid Normal
Day since April 15, 2021
Model-3 (Home&Vac)
New Cases-Data
Number of New Cases (daily)
Deaths-Data
Number of Deaths (daily)
R0(t)(Model 3)
R0(t) 95% CI
R0(H,V)
R0 (Model 3)
R0=1
Vc(-14d)%
Home-stay (H%) (daily)

Figure 9

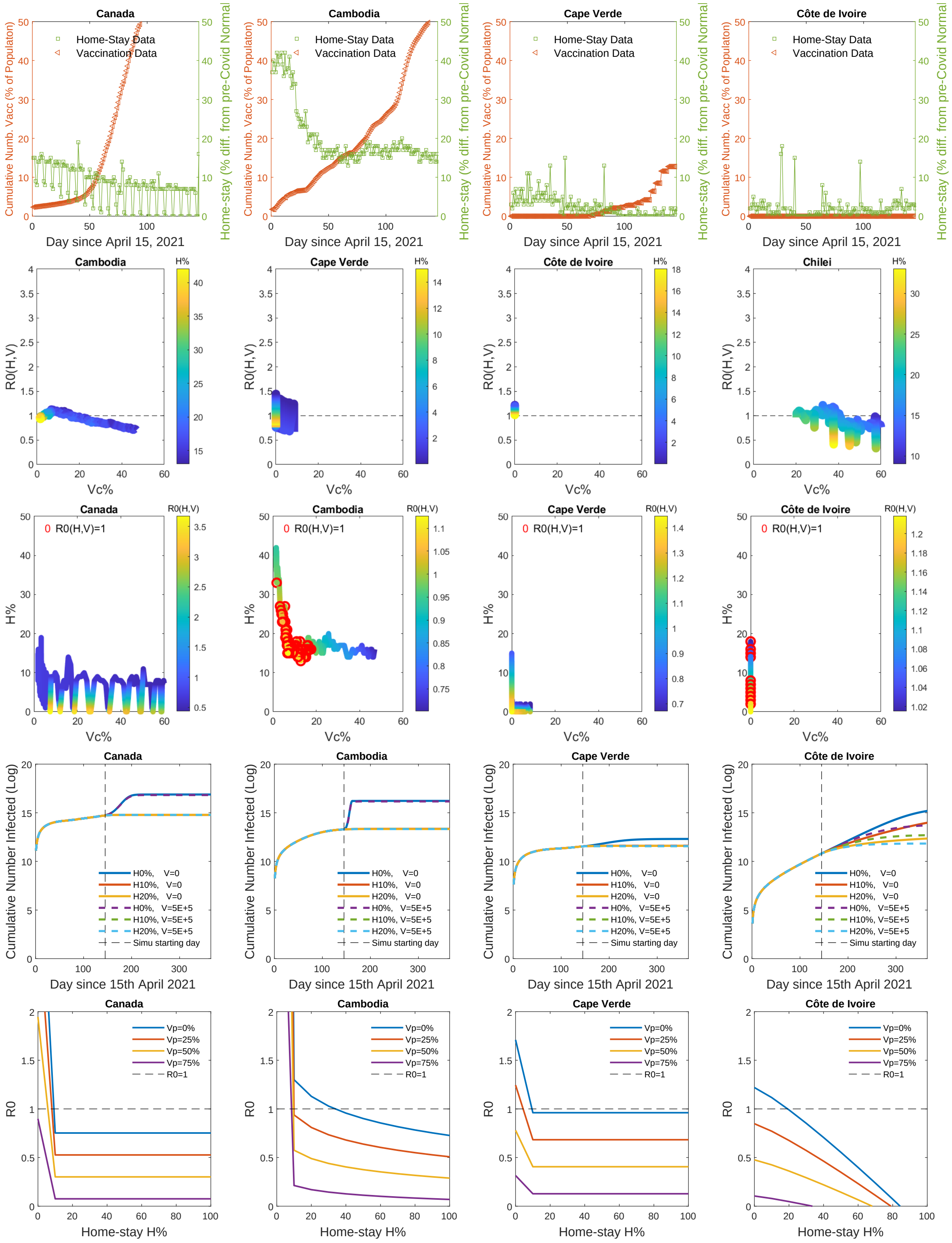
Canada
Cambodia
Cape Verde
Côte de Ivoire
Home-Stay Data
Vaccination Data
Cumulative Numb. Vacc (% of Populaton)
Home-stay (% diff. from pre-Covid Normal
Day since April 15, 2021
Chilei
R0(H,V)
H%
Vc%
0 R0(H,V)=1
Cumulative Number Infected (Log)
H0%, V=0
H10%, V=0
H20%, V=0
H0%, V=5E+5
H10%, V=5E+5
H20%, V=5E+5
Simu starting day
Day since 15th April 2021
R0
Vp=0%
Vp=25%
Vp=50%
Vp=75%
R0=1
Home-stay H%

Figure 10

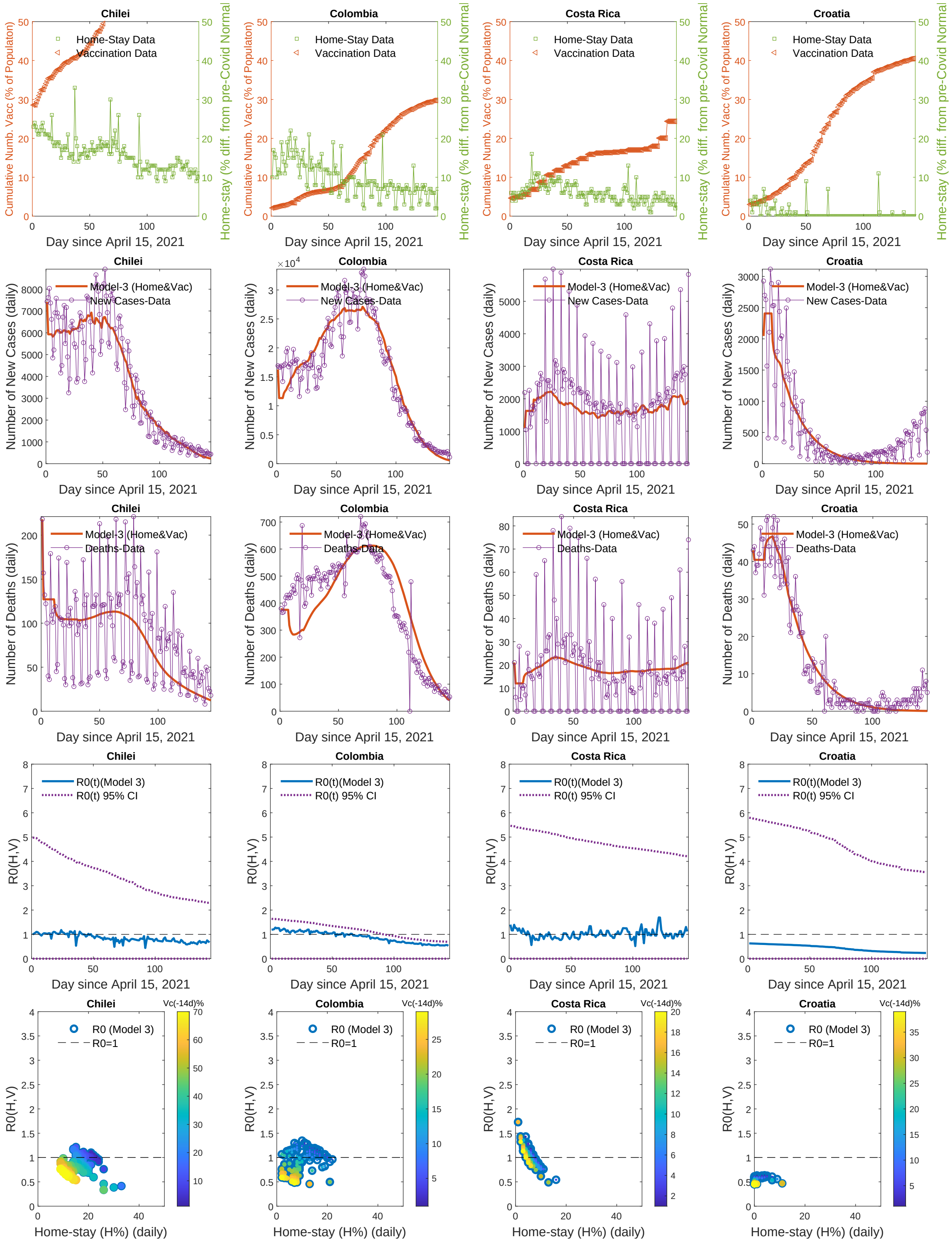
Chilei
Colombia
Costa Rica
Croatia
Home-Stay Data
Vaccination Data
Cumulative Numb. Vacc (% of Populaton)
Home-stay (% diff. from pre-Covid Normal
Day since April 15, 2021
Model-3 (Home&Vac)
New Cases-Data
Number of New Cases (daily)
Deaths-Data
Number of Deaths (daily)
R0(t)(Model 3)
R0(t) 95% CI
R0(H,V)
R0 (Model 3)
R0=1
Vc(-14d)%
Home-stay (H%) (daily)

Figure 11

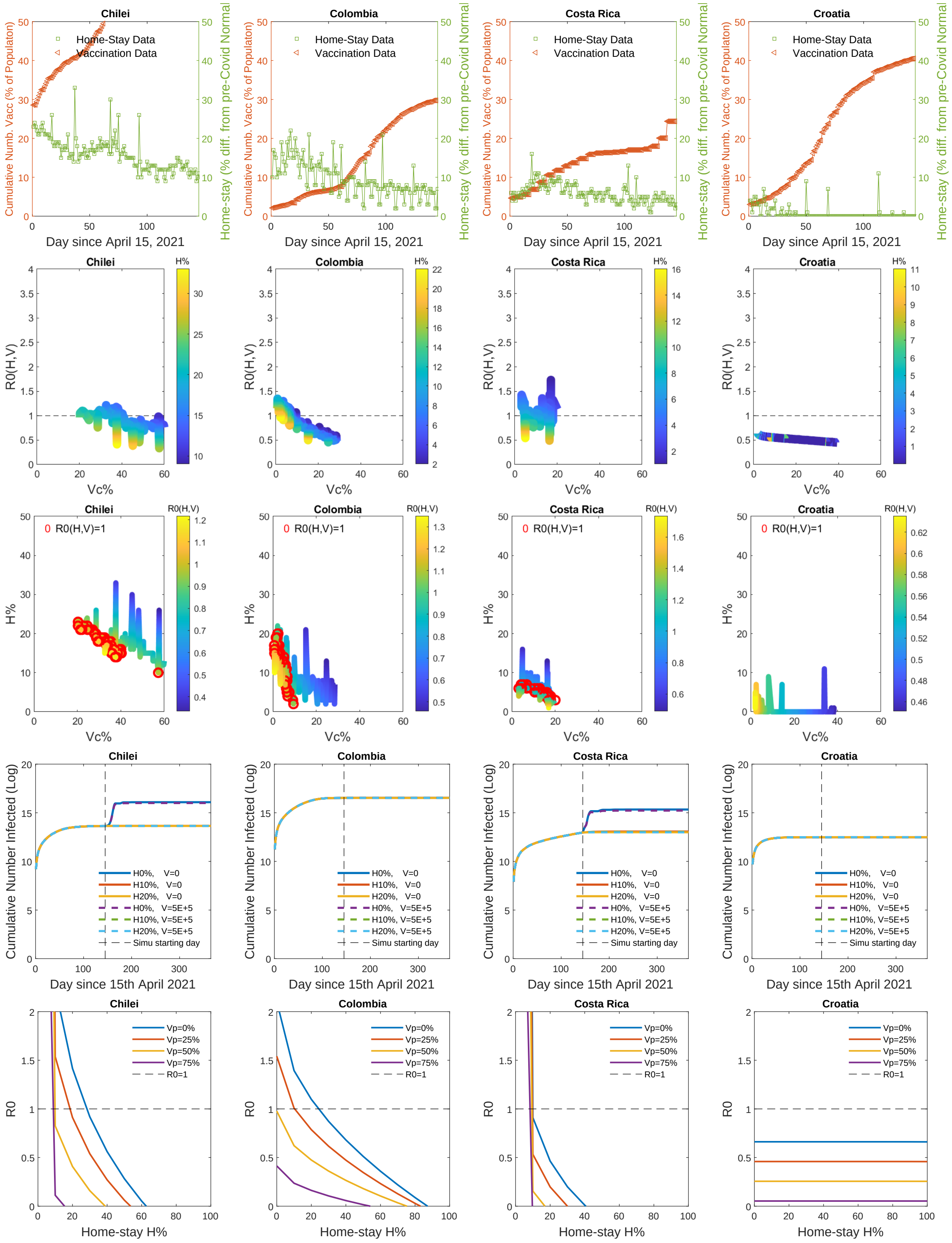
Chilei
Colombia
Costa Rica
Croatia
Home-Stay Data
Vaccination Data
Cumulative Numb. Vacc (% of Populaton)
Home-stay (% diff. from pre-Covid Normal
Day since April 15, 2021
R0(H,V)
H%
Vc%
R0(H,V)=1
Cumulative Number Infected (Log)
H0%, V=0
H10%, V=0
H20%, V=0
H0%, V=5E+5
H10%, V=5E+5
H20%, V=5E+5
Simu starting day
Day since 15th April 2021
R0
Vp=0%
Vp=25%
Vp=50%
Vp=75%
R0=1
Home-stay H%

Figure 12

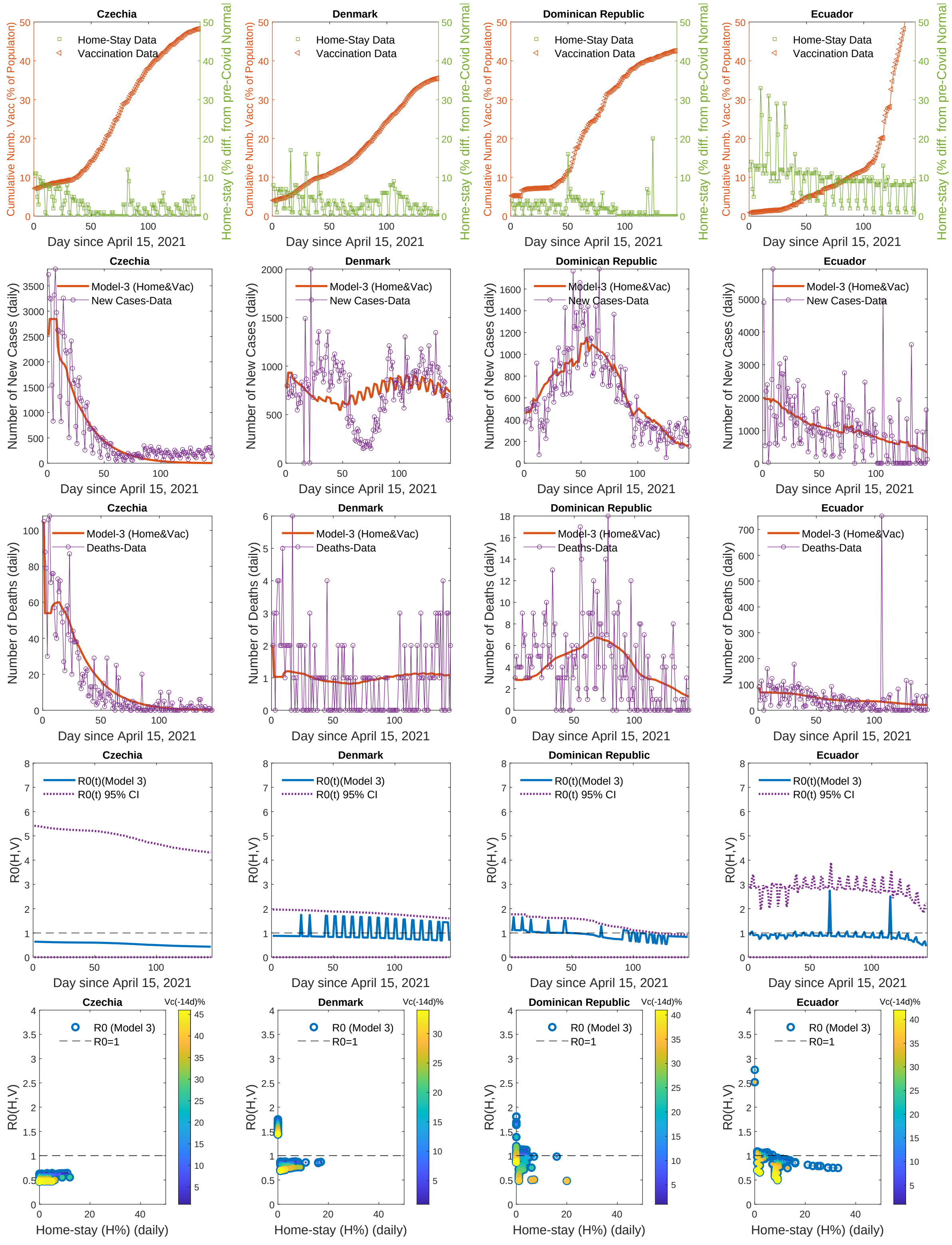

Czechia
Denmark
Dominican Republic
Ecuador
Home-Stay Data
Vaccination Data
Cumulative Numb. Vacc (% of Populaton)
Home-stay (% diff. from pre-Covid Normal
Day since April 15, 2021
Model-3 (Home&Vac)
New Cases-Data
Number of New Cases (daily)
Deaths-Data
Number of Deaths (daily)
R0(t)(Model 3)
R0(t) 95% CI
R0(H,V)
R0 (Model 3)
R0=1
Vc(-14d)%
Home-stay (H%) (daily)

Figure 13

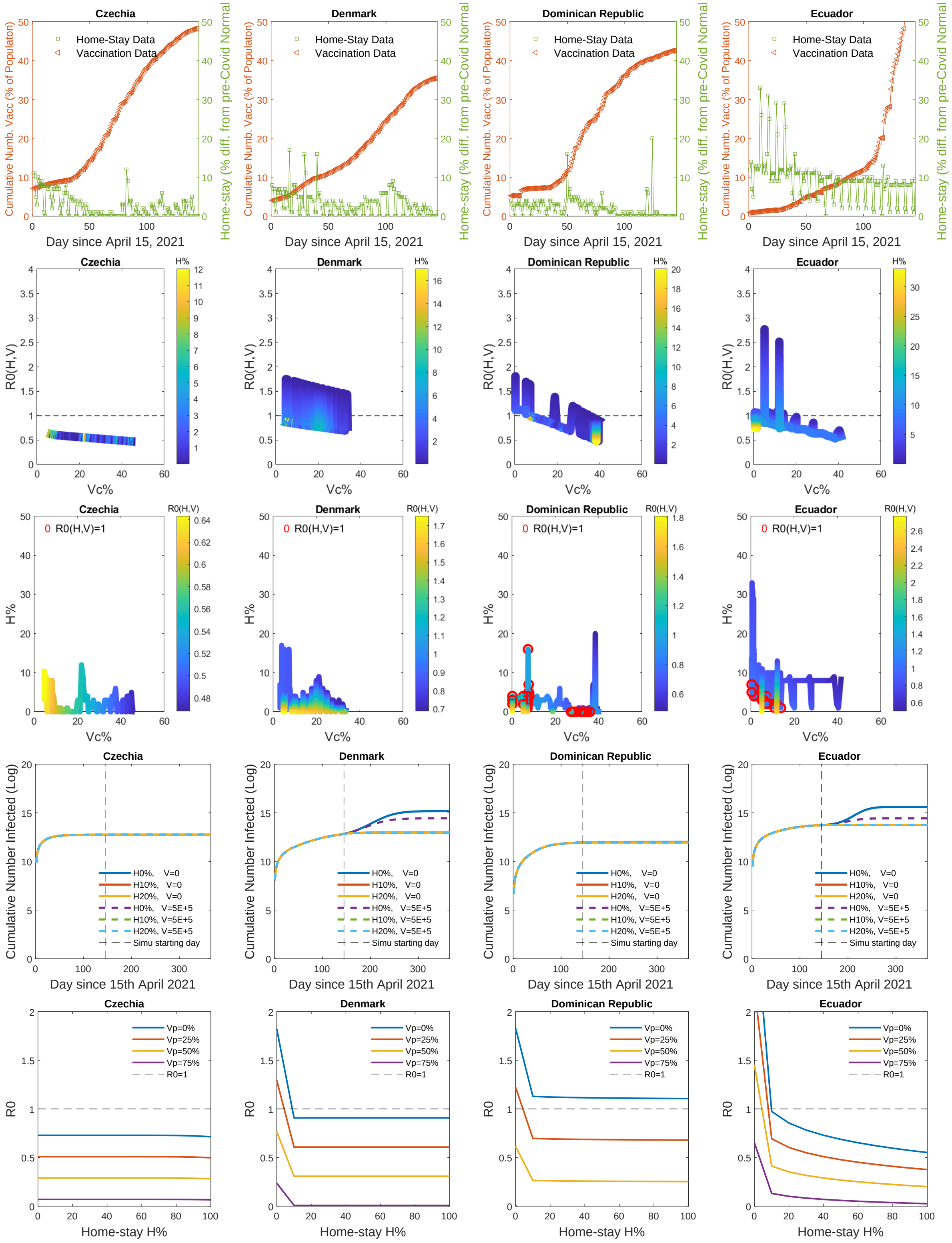
Czechia
Denmark
Dominican Republic
Ecuador
Home-Stay Data
Vaccination Data
Cumulative Numb. Vacc (% of Populaton)
Home-stay (% diff. from pre-Covid Normal
Day since April 15, 2021
R0(H,V)
H%
Vc%
0 R0(H,V)=1
Cumulative Number Infected (Log)
H0%, V=0
H10%, V=0
H20%, V=0
H0%, V=5E+5
H10%, V=5E+5
H20%, V=5E+5
Simu starting day
Day since 15th April 2021
R0
Vp=0%
Vp=25%
Vp=50%
Vp=75%
R0=1
Home-stay H%

Figure 14

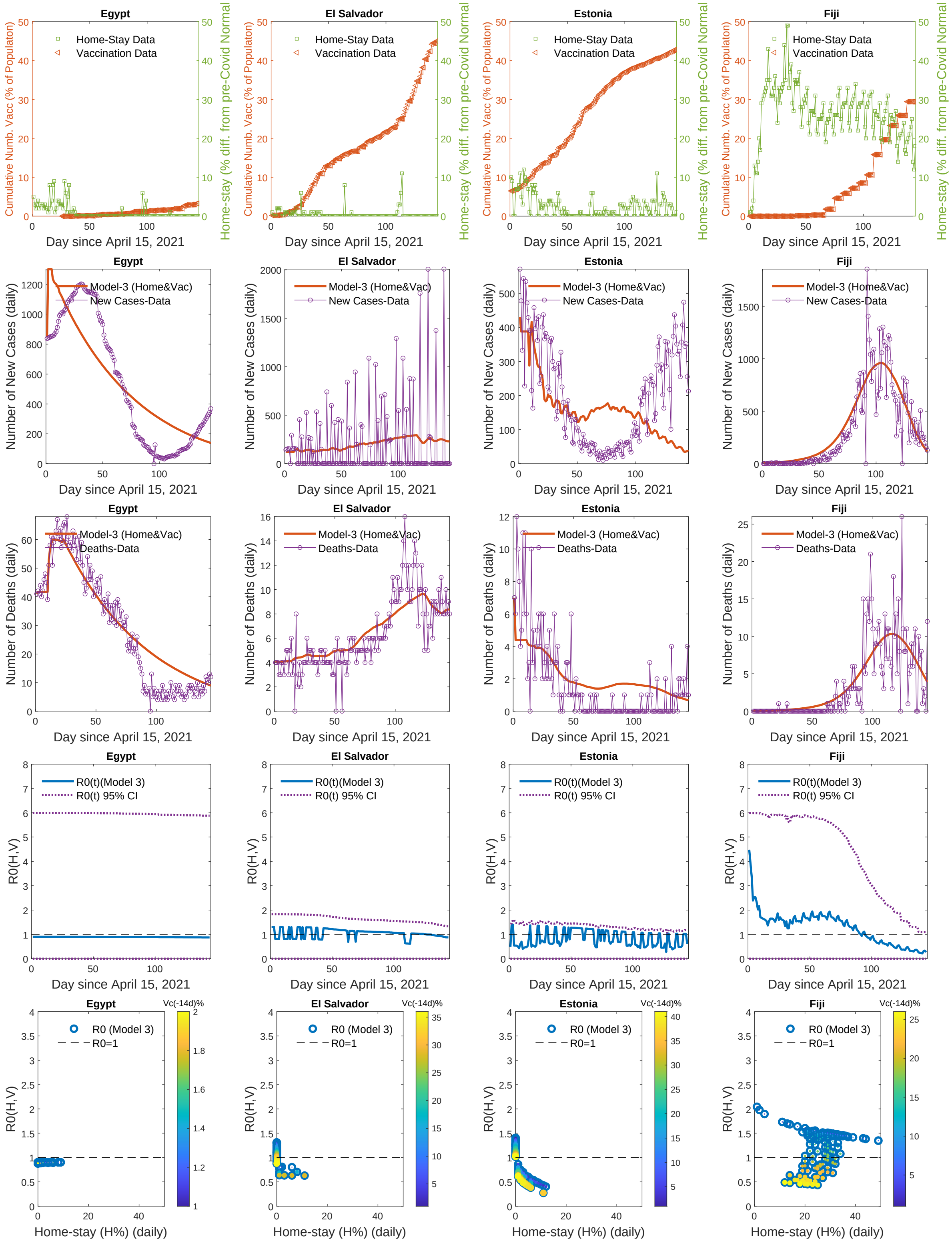
Egypt
El Salvador
Estonia
Fiji
Home-Stay Data
Vaccination Data
Cumulative Numb. Vacc (% of Populaton)
Home-stay (% diff. from pre-Covid Normal
Day since April 15, 2021
Model-3 (Home&Vac)
New Cases-Data
Number of New Cases (daily)
Deaths-Data
Number of Deaths (daily)
R0(t)(Model 3)
R0(t) 95% CI
R0(H,V)
R0 (Model 3)
R0=1
Vc(-14d)%
Home-stay (H%) (daily)

Figure 15

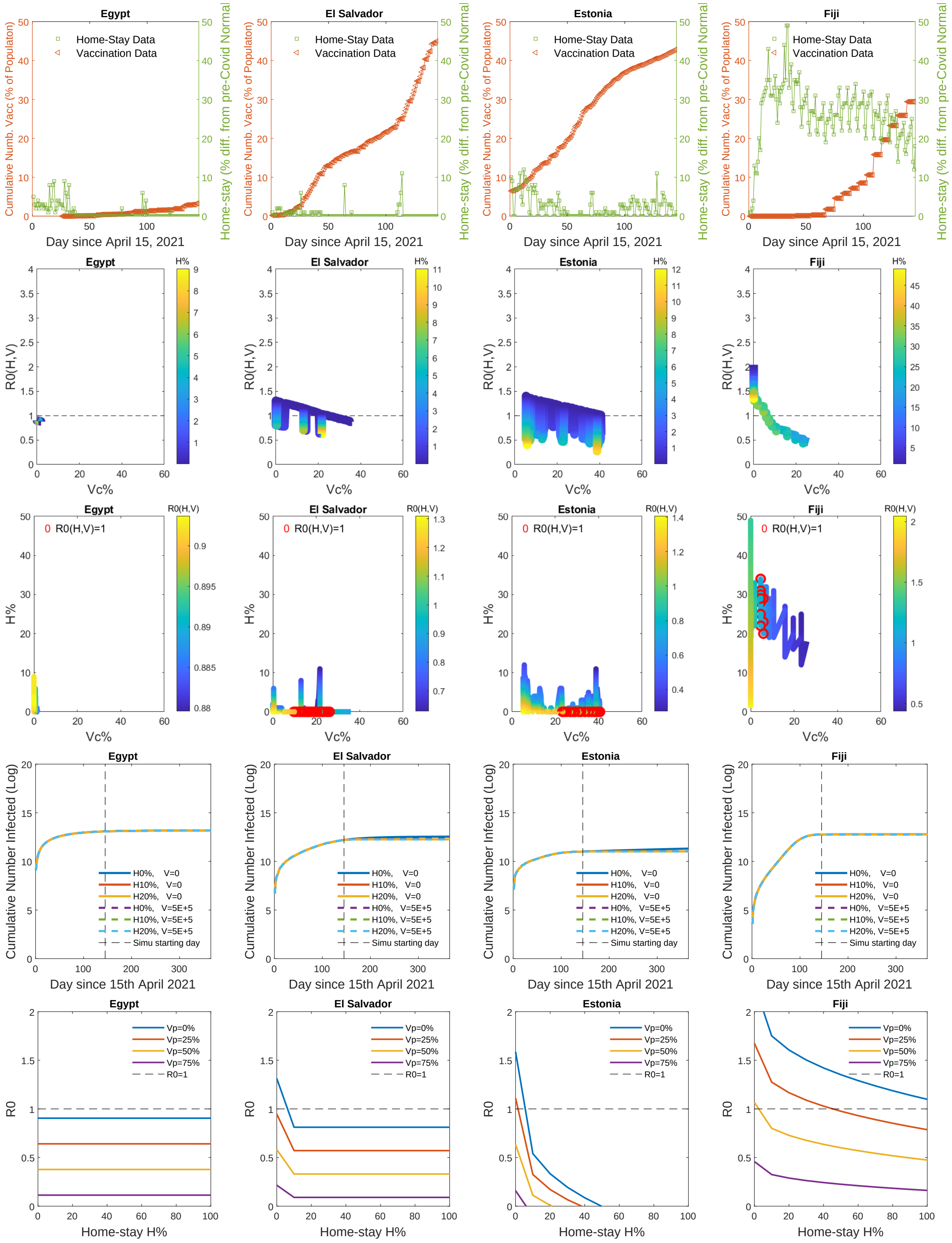
Egypt
El Salvador
Estonia
Fiji
Home-Stay Data
Vaccination Data
Cumulative Numb. Vacc (% of Populaton)
Home-stay (% diff. from pre-Covid Normal
Day since April 15, 2021
R0(H,V)
H%
Vc%
0 R0(H,V)=1
Cumulative Number Infected (Log)
H0%, V=0
H10%, V=0
H20%, V=0
H0%, V=5E+5
H10%, V=5E+5
H20%, V=5E+5
Simu starting day
Day since 15th April 2021
R0
Vp=0%
Vp=25%
Vp=50%
Vp=75%
R0=1
Home-stay H%

Figure 16

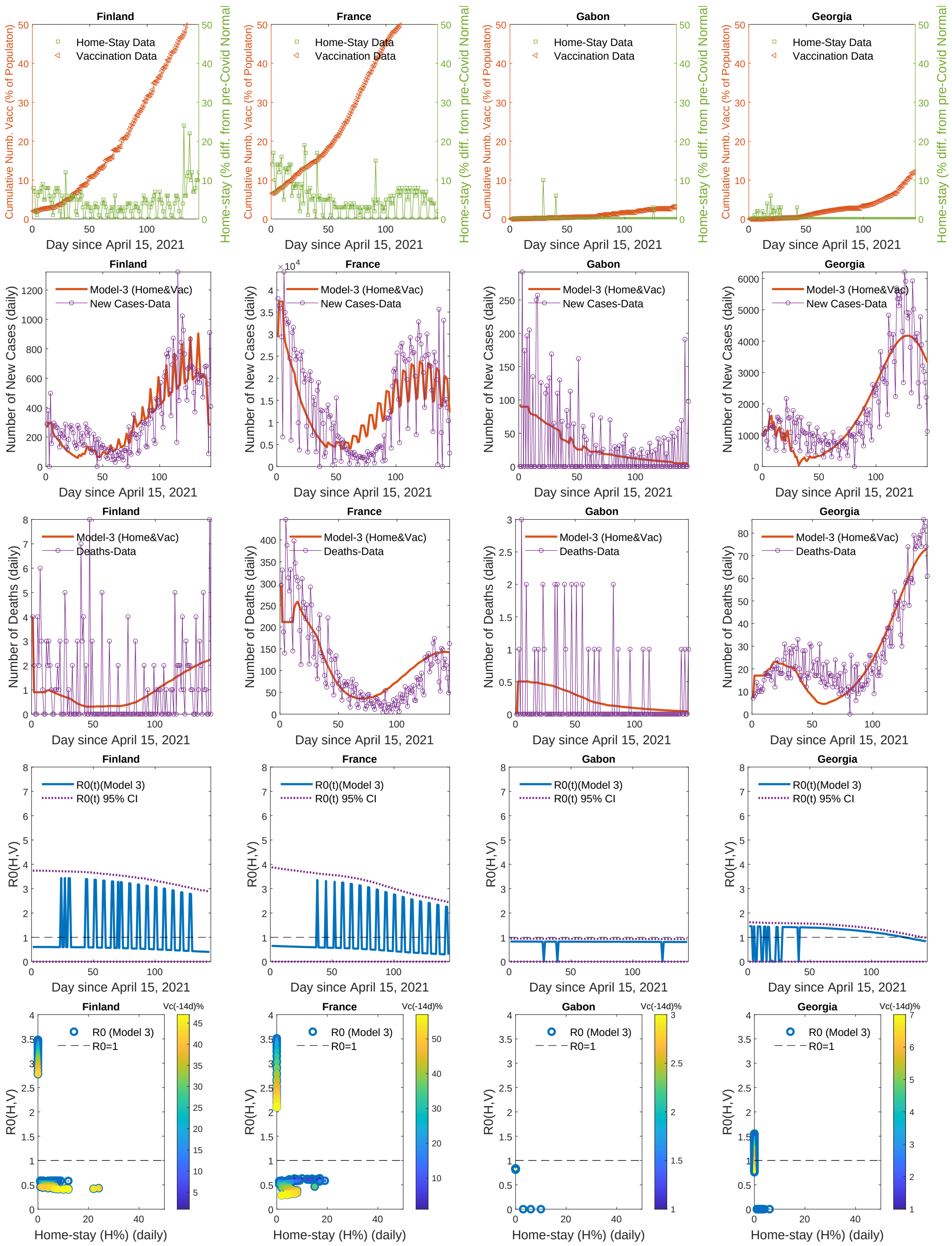
Finland
France
Gabon
Georgia
Home-Stay Data
Vaccination Data
Cumulative Numb. Vacc (% of Populaton)
Home-stay (% diff. from pre-Covid Normal
Day since April 15, 2021
Model-3 (Home&Vac)
New Cases-Data
Number of New Cases (daily)
Deaths-Data
Number of Deaths (daily)
R0(t)(Model 3)
R0(t) 95% CI
R0(H,V)
R0 (Model 3)
R0=1
Vc(-14d)%
Home-stay (H%) (daily)

Figure 17

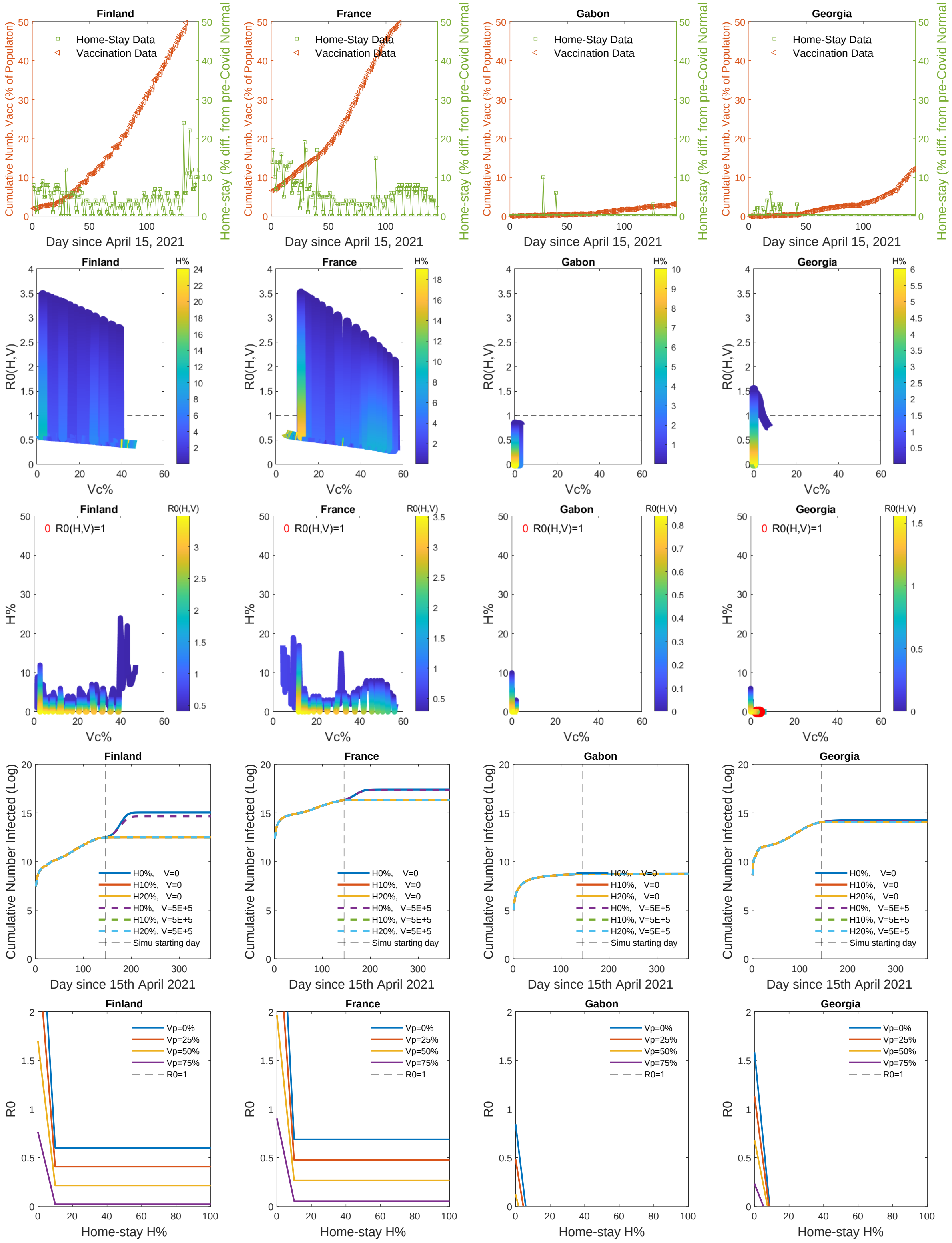
Finland
France
Gabon
Georgia
Home-Stay Data
Vaccination Data
Cumulative Numb. Vacc (% of Populaton)
Home-stay (% diff. from pre-Covid Normal
Day since April 15, 2021
R0(H,V)
H%
Vc%
0 R0(H,V)=1
Cumulative Number Infected (Log)
Day since 15th April 2021
H0%, V=0
H10%, V=0
H20%, V=0
H0%, V=5E+5
H10%, V=5E+5
H20%, V=5E+5
Simu starting day
R0
Vp=0%
Vp=25%
Vp=50%
Vp=75%
R0=1
Home-stay H%

Figure 18

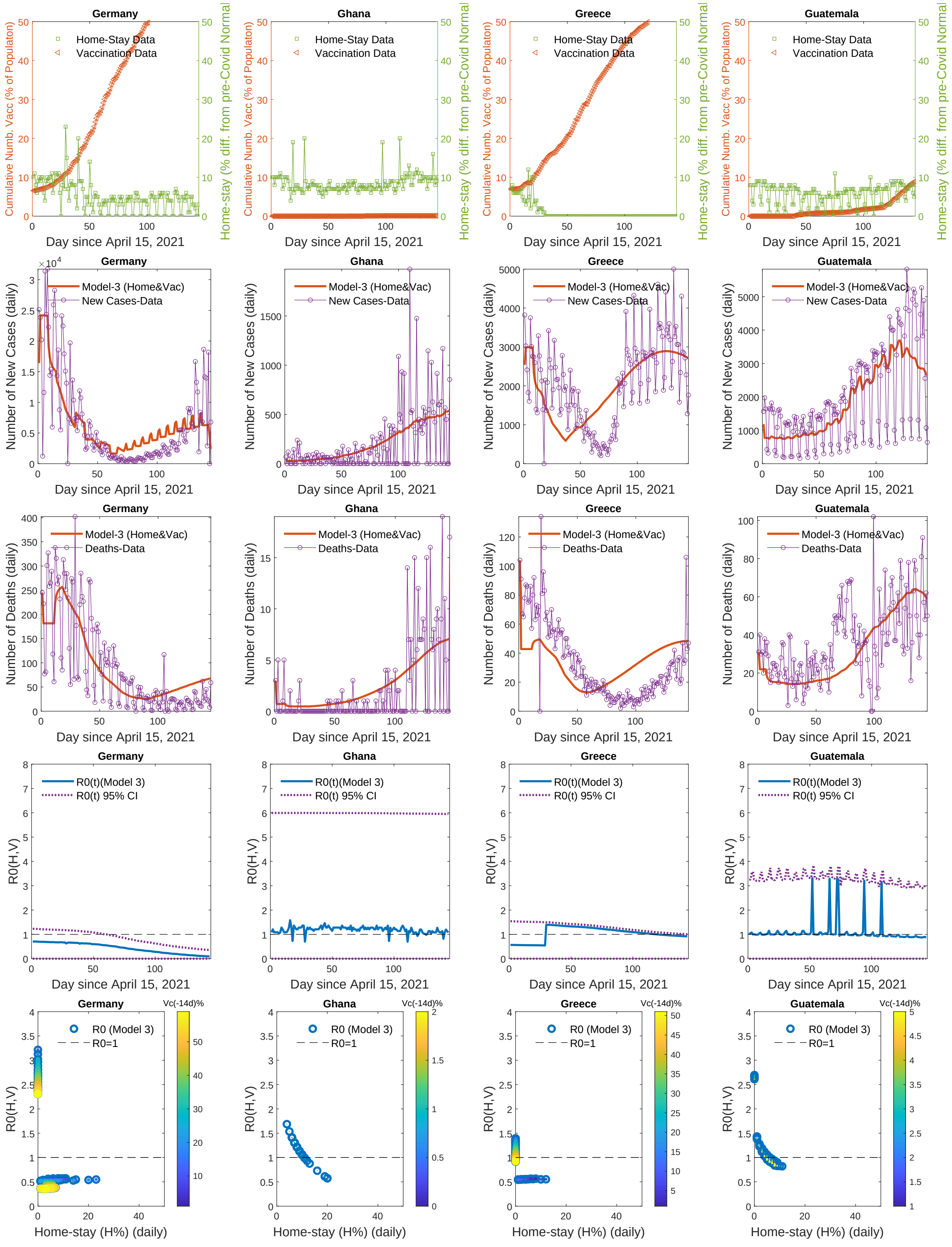

Germany
Ghana
Greece
Guatemala
Home-Stay Data
Vaccination Data
Cumulative Numb. Vacc (% of Populaton)
Home-stay (% diff. from pre-Covid Normal
Day since April 15, 2021
Model-3 (Home&Vac)
New Cases-Data
Number of New Cases (daily)
Deaths-Data
Number of Deaths (daily)
R0(t)(Model 3)
R0(t) 95% CI
R0(H,V)
R0 (Model 3)
R0=1
Vc(-14d)%
Home-stay (H%) (daily)

Figure 19

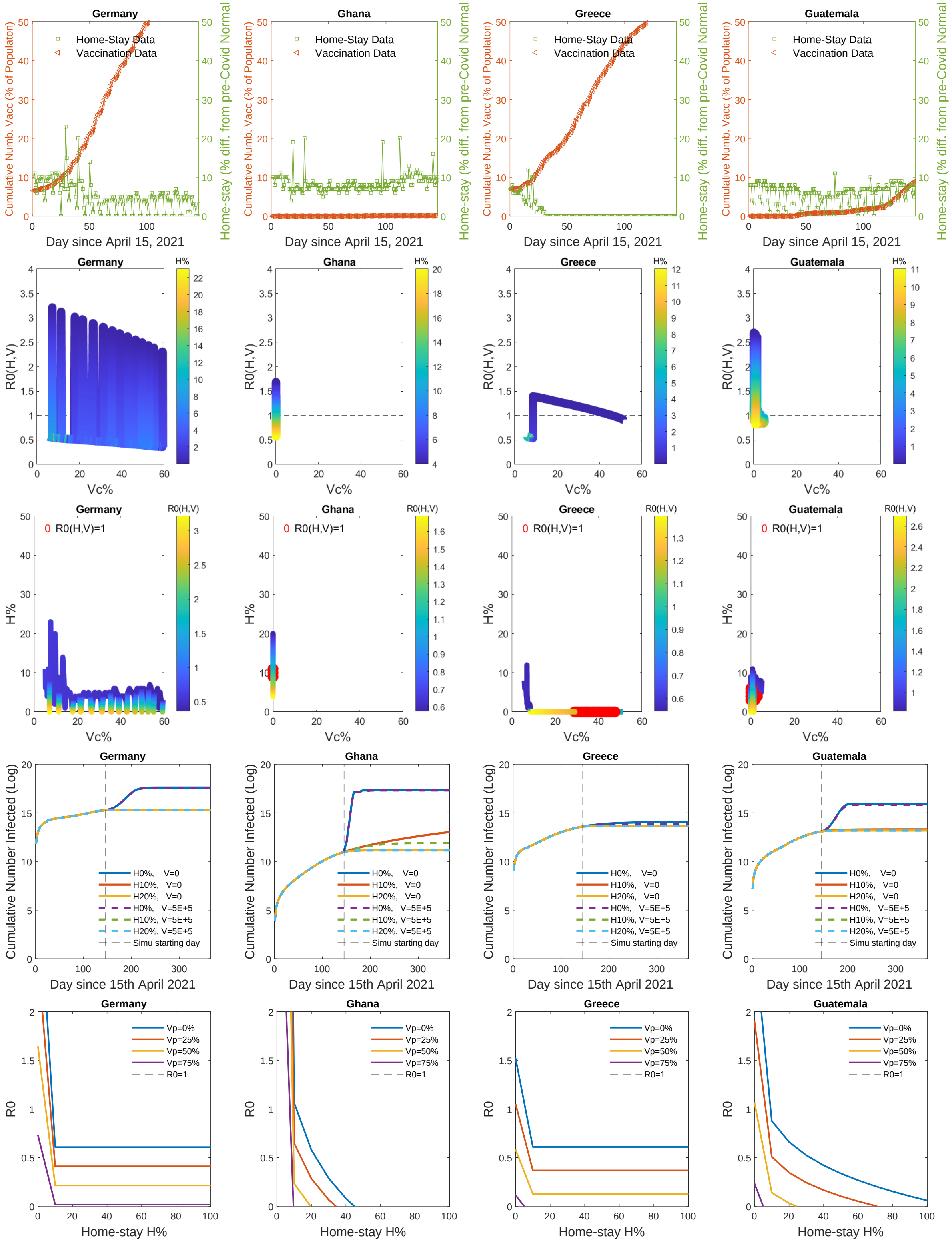

Germany
Ghana
Greece
Guatemala
Home-Stay Data
Vaccination Data
Cumulative Numb. Vacc (% of Populaton)
Home-stay (% diff. from pre-Covid Normal
Day since April 15, 2021
R0(H,V)
H%
Vc%
R0(H,V)=1
Cumulative Number Infected (Log)
H0%, V=0
H10%, V=0
H20%, V=0
H0%, V=5E+5
H10%, V=5E+5
H20%, V=5E+5
Simu starting day
Day since 15th April 2021
Vp=0%
Vp=25%
Vp=50%
Vp=75%
R0=1
R0
Home-stay H%

Figure 20

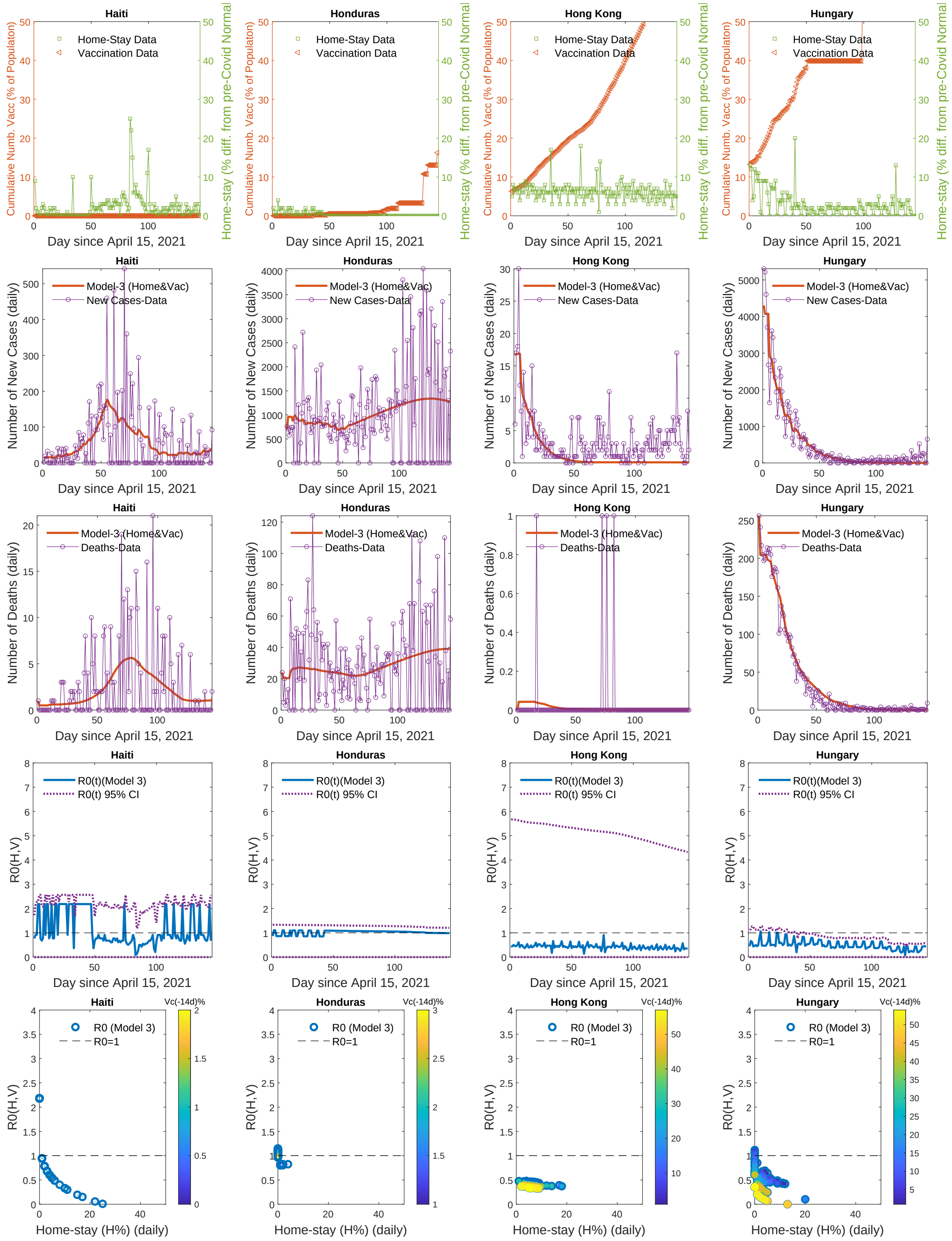
Haiti
Honduras
Hong Kong
Hungary
Home-Stay Data
Vaccination Data
Cumulative Numb. Vacc (% of Populaton)
Home-stay (% diff. from pre-Covid Normal
Day since April 15, 2021
Model-3 (Home&Vac)
New Cases-Data
Number of New Cases (daily)
Deaths-Data
Number of Deaths (daily)
R0(t)(Model 3)
R0(t) 95% CI
R0(H,V)
R0 (Model 3)
R0=1
Vc(-14d)%
Home-stay (H%) (daily)

Figure 21

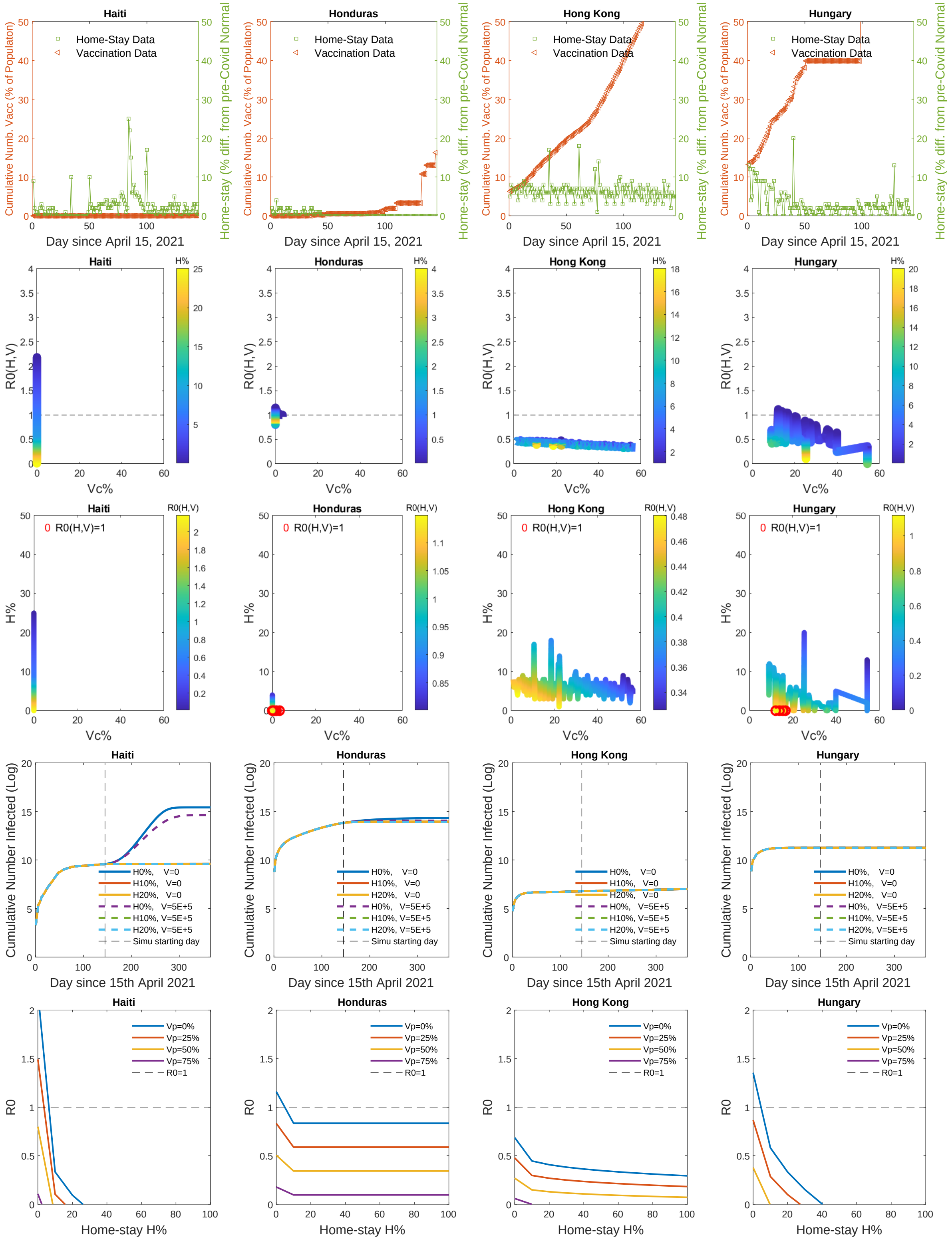
Haiti
Honduras
Hong Kong
Hungary
Home-Stay Data
Vaccination Data
Cumulative Numb. Vacc (% of Populaton)
Home-stay (% diff. from pre-Covid Normal
Day since April 15, 2021
R0(H,V)
H%
Vc%
0 R0(H,V)=1
Cumulative Number Infected (Log)
H0%, V=0
H10%, V=0
H20%, V=0
H0%, V=5E+5
H10%, V=5E+5
H20%, V=5E+5
Simu starting day
Day since 15th April 2021
R0
Vp=0%
Vp=25%
Vp=50%
Vp=75%
R0=1
Home-stay H%

Figure 22

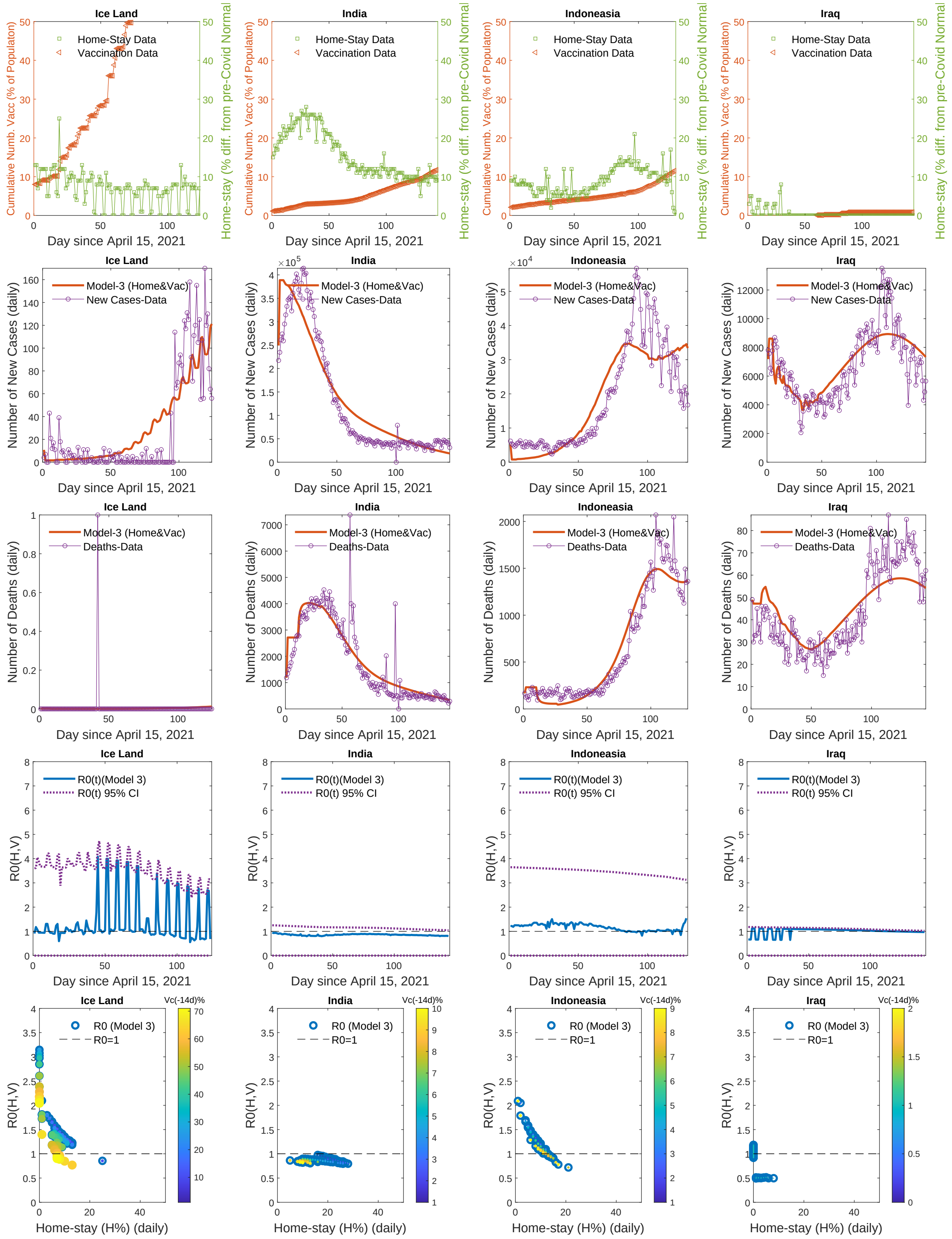

Ice Land
India
Indoneasia
Iraq
Home-Stay Data
Vaccination Data
Cumulative Numb. Vacc (% of Populaton)
Home-stay (% diff. from pre-Covid Normal
Day since April 15, 2021
Model-3 (Home&Vac)
New Cases-Data
Number of New Cases (daily)
Deaths-Data
Number of Deaths (daily)
R0(t)(Model 3)
R0(t) 95% CI
R0(H,V)
R0 (Model 3)
R0=1
Vc(-14d)%
Home-stay (H%) (daily)

Figure 23

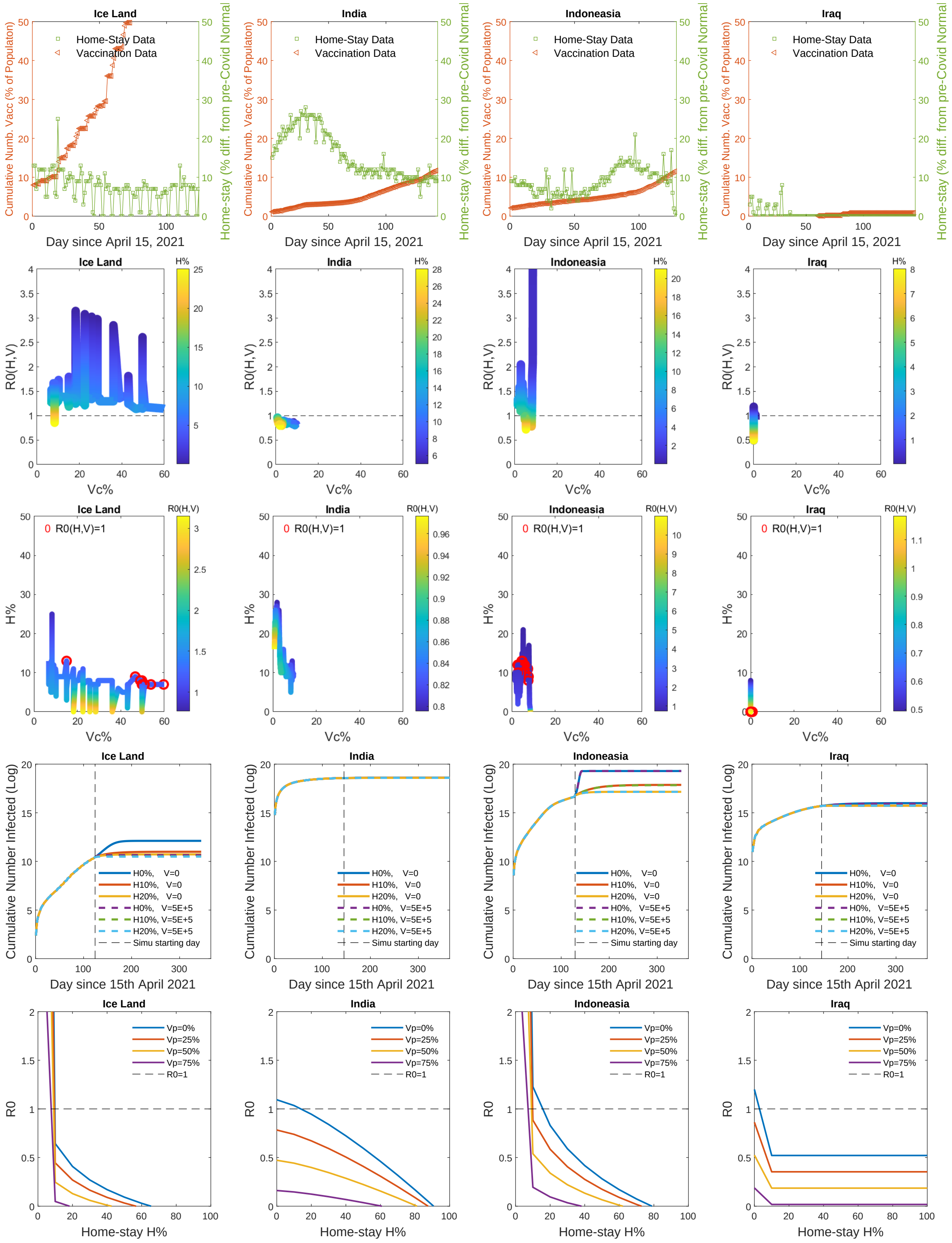

Ice Land
India
Indoneasia
Iraq
Home-Stay Data
Vaccination Data
Cumulative Numb. Vacc (% of Populaton)
Home-stay (% diff. from pre-Covid Normal
Day since April 15, 2021
R0(H,V)
Vc%
H%
R0(H,V)=1
Cumulative Number Infected (Log)
H0%, V=0
H10%, V=0
H20%, V=0
H0%, V=5E+5
H10%, V=5E+5
H20%, V=5E+5
Simu starting day
Day since 15th April 2021
R0
Vp=0%
Vp=25%
Vp=50%
Vp=75%
R0=1
Home-stay H%

Figure 24

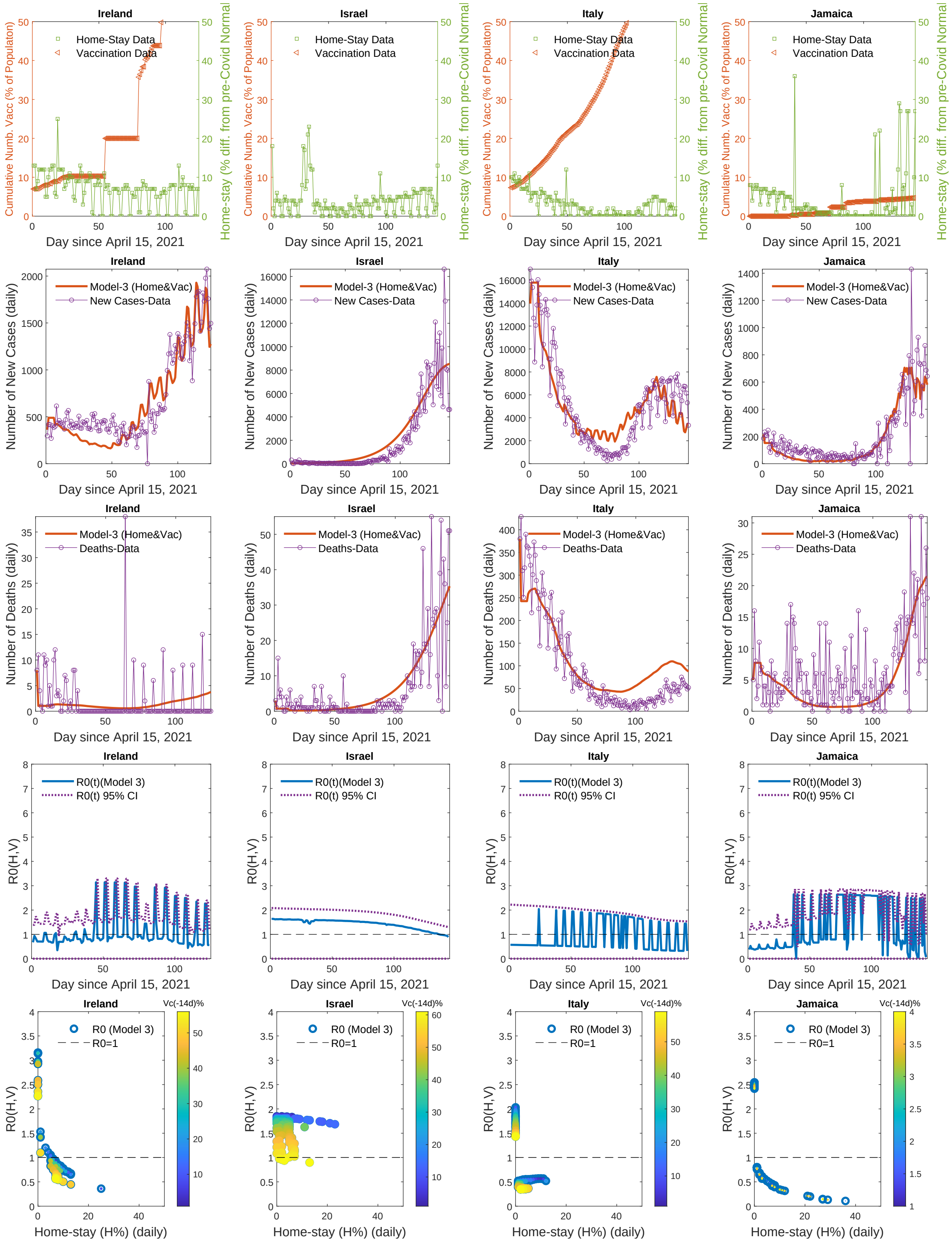
Ireland
Israel
Italy
Jamaica
Home-Stay Data
Vaccination Data
Cumulative Numb. Vacc (% of Populaton)
Home-stay (% diff. from pre-Covid Normal
Day since April 15, 2021
Model-3 (Home&Vac)
New Cases-Data
Number of New Cases (daily)
Deaths-Data
Number of Deaths (daily)
R0(t)(Model 3)
R0(t) 95% CI
R0(H,V)
R0 (Model 3)
R0=1
Vc(-14d)%
Home-stay (H%) (daily)

Figure 25

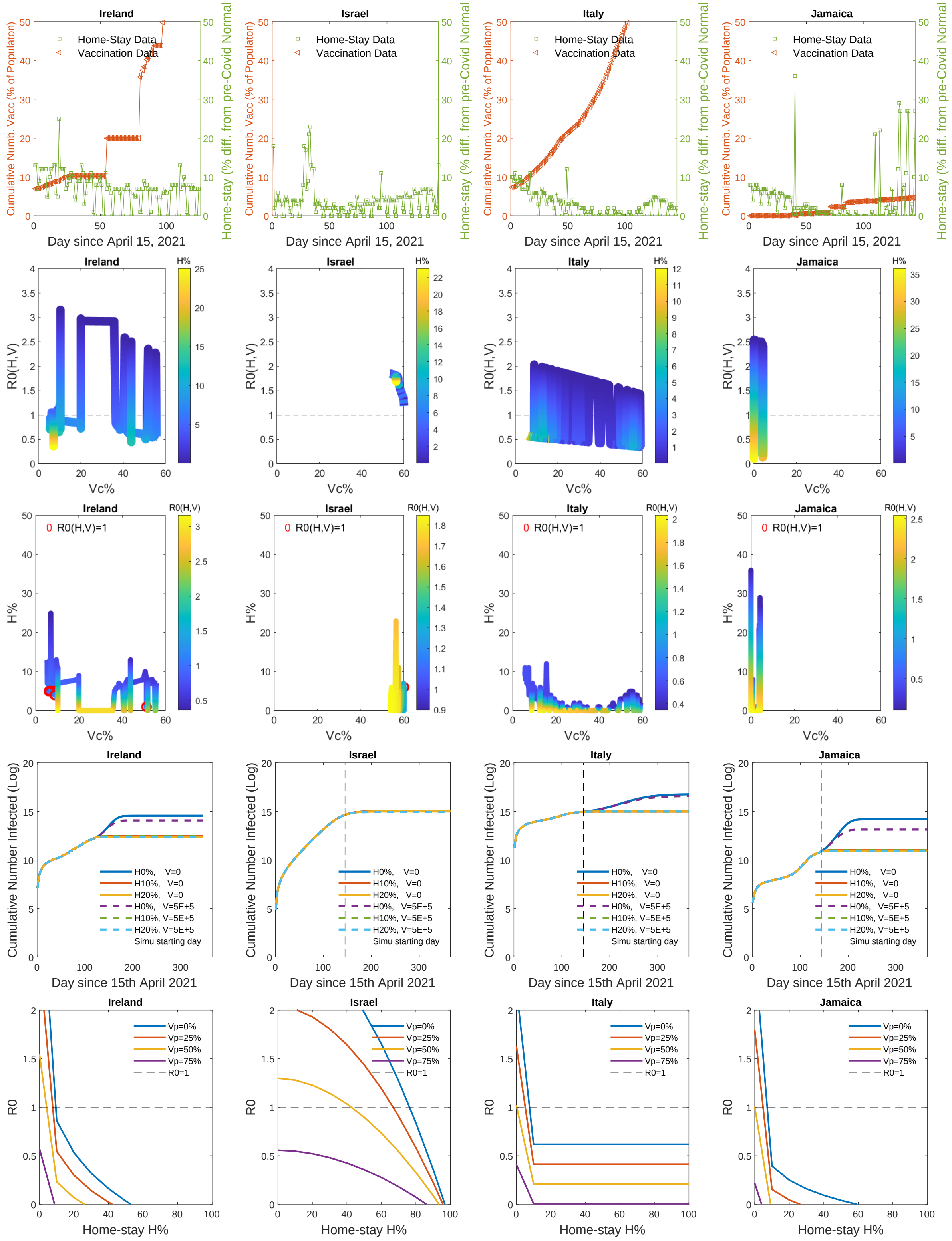
Ireland
Israel
Italy
Jamaica
Home-Stay Data
Vaccination Data
Cumulative Numb. Vacc (% of Populaton)
Home-stay (% diff. from pre-Covid Normal
Day since April 15, 2021
R0(H,V)
H%
Vc%
0 R0(H,V)=1
Cumulative Number Infected (Log)
H0%, V=0
H10%, V=0
H20%, V=0
H0%, V=5E+5
H10%, V=5E+5
H20%, V=5E+5
Simu starting day
Day since 15th April 2021
R0
Vp=0%
Vp=25%
Vp=50%
Vp=75%
R0=1
Home-stay H%

Figure 26

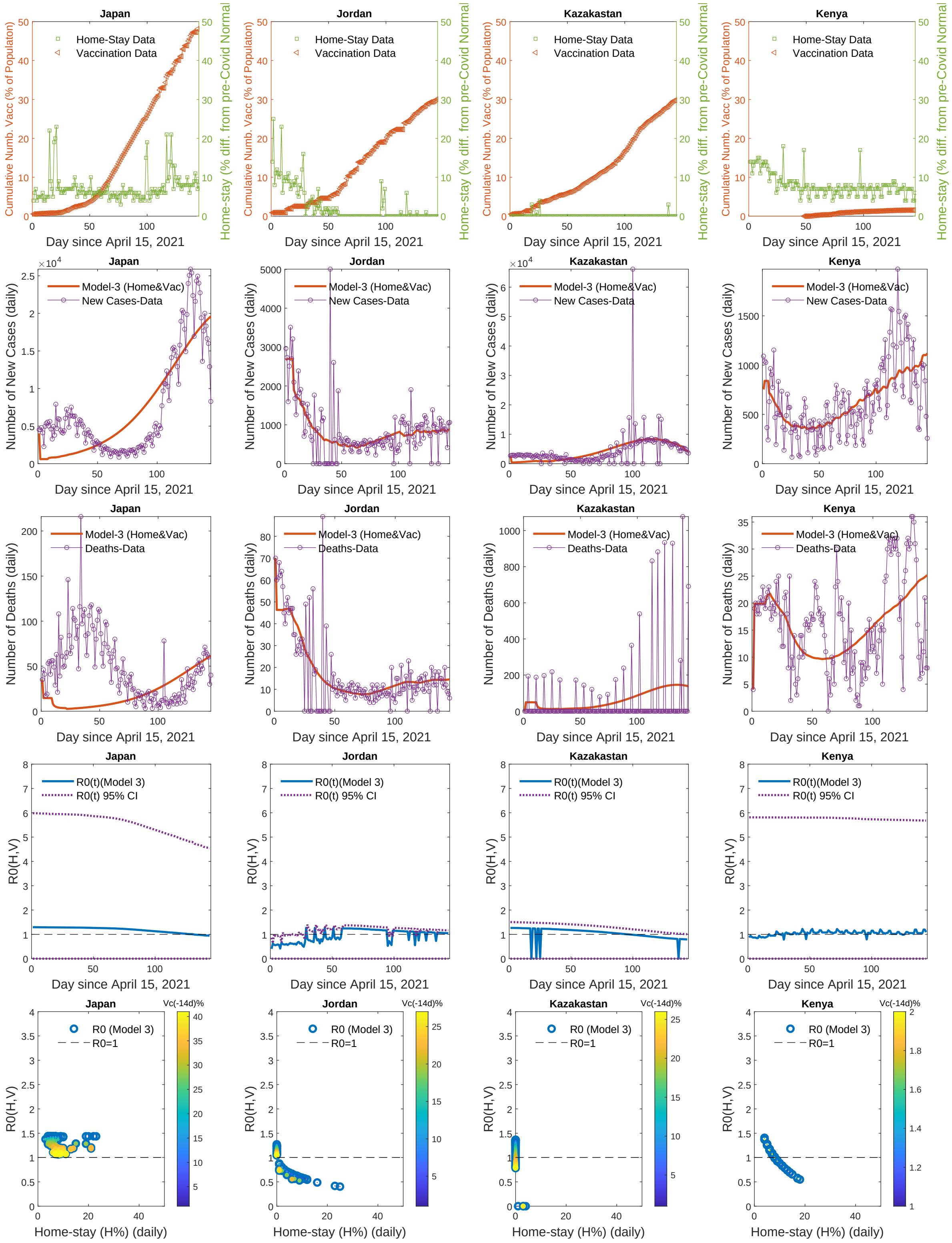
Japan
Jordan
Kazakastan
Kenya
Home-Stay Data
Vaccination Data
Cumulative Numb. Vacc (% of Populaton)
Home-stay (% diff. from pre-Covid Normal
Day since April 15, 2021
Model-3 (Home&Vac)
New Cases-Data
Number of New Cases (daily)
Deaths-Data
Number of Deaths (daily)
R0(t)(Model 3)
R0(t) 95% CI
R0(H,V)
R0 (Model 3)
R0=1
Vc(-14d)%
Home-stay (H%) (daily)

Figure 27

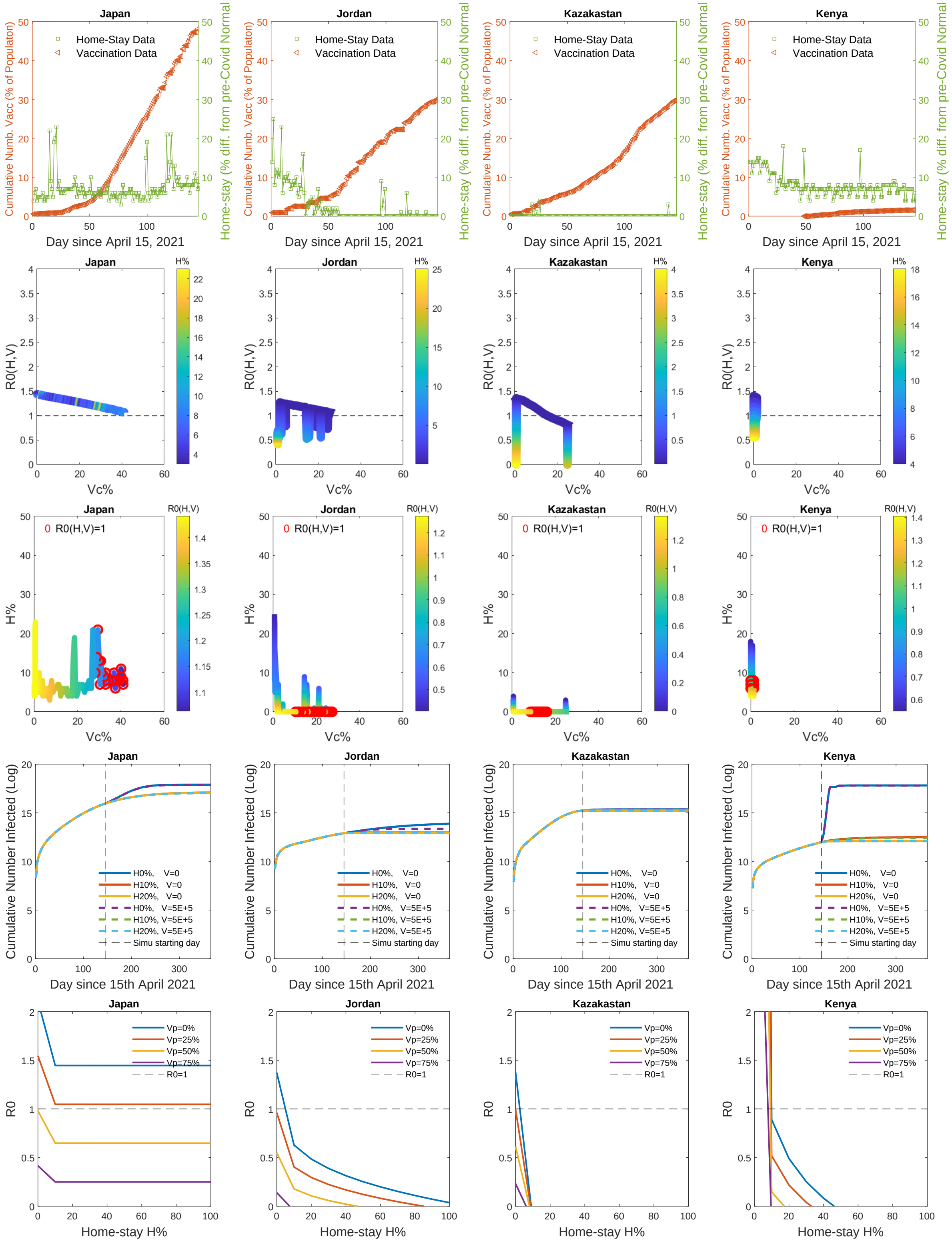
Japan
Jordan
Kazakastan
Kenya
Home-Stay Data
Vaccination Data
Cumulative Numb. Vacc (% of Populaton)
Home-stay (% diff. from pre-Covid Normal
Day since April 15, 2021
R0(H,V)
H%
Vc%
R0(H,V)=1
Cumulative Number Infected (Log)
H0%, V=0
H10%, V=0
H20%, V=0
H0%, V=5E+5
H10%, V=5E+5
H20%, V=5E+5
Simu starting day
Day since 15th April 2021
R0
Vp=0%
Vp=25%
Vp=50%
Vp=75%
R0=1
Home-stay H%

Figure 28

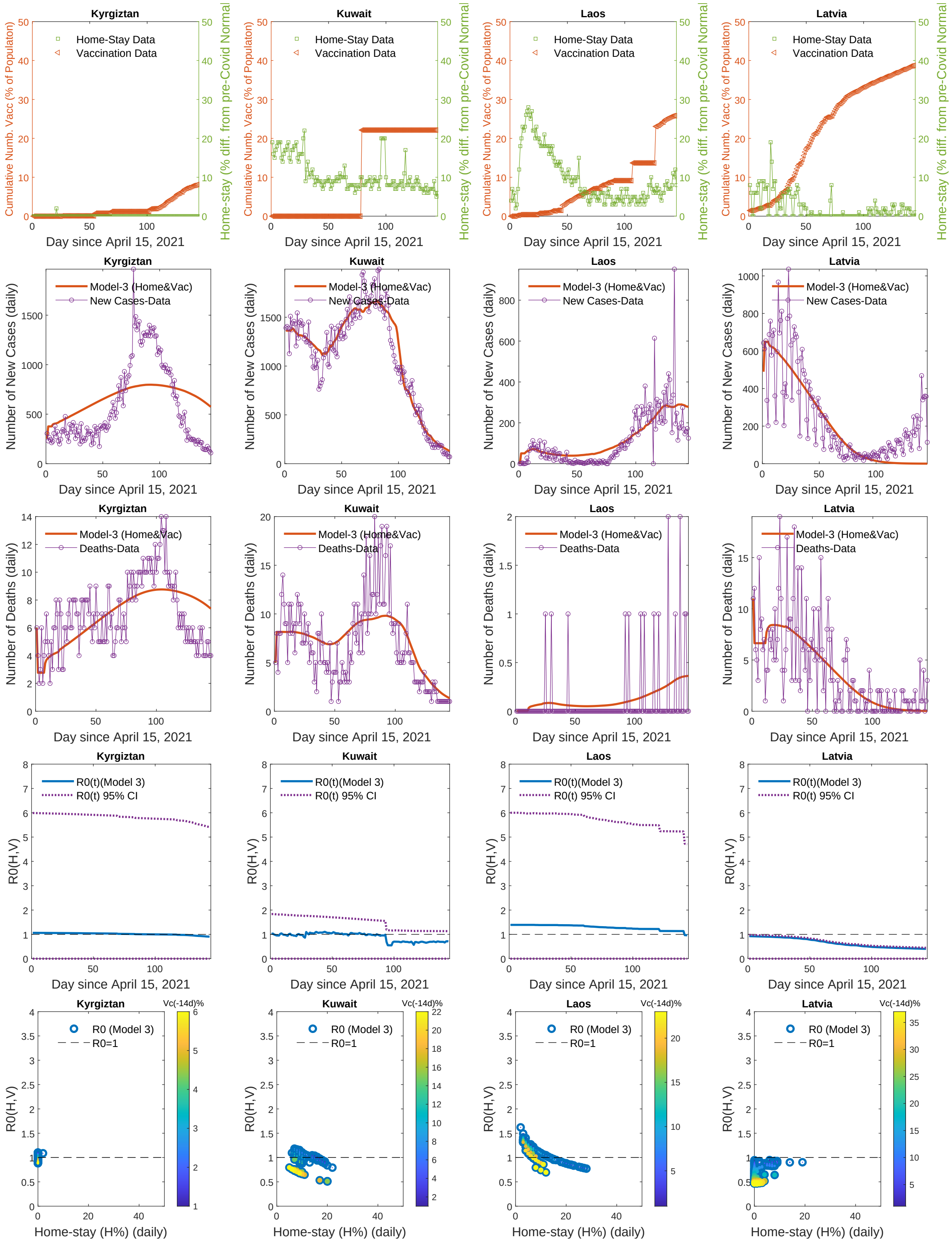
Kyrgiztan
Kuwait
Laos
Latvia
Home-Stay Data
Vaccination Data
Cumulative Numb. Vacc (% of Populaton)
Home-stay (% diff. from pre-Covid Normal
Day since April 15, 2021
Model-3 (Home&Vac)
New Cases-Data
Number of New Cases (daily)
Deaths-Data
Number of Deaths (daily)
R0(t)(Model 3)
R0(t) 95% CI
R0(H,V)
R0 (Model 3)
R0=1
Vc(-14d)%
Home-stay (H%) (daily)

Figure 29

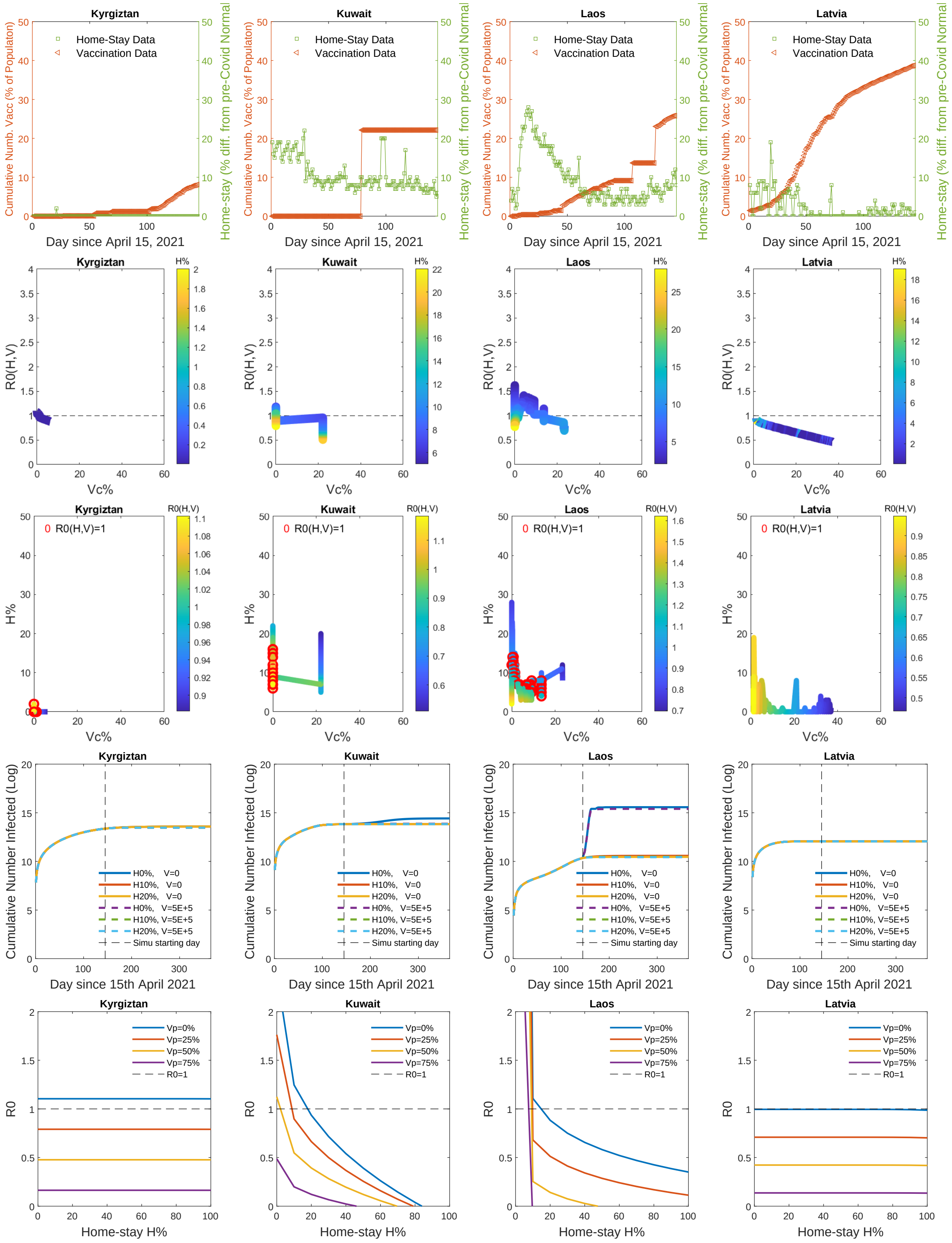
Kyrgiztan
Kuwait
Laos
Latvia
Home-Stay Data
Vaccination Data
Cumulative Numb. Vacc (% of Populaton)
Home-stay (% diff. from pre-Covid Normal
Day since April 15, 2021
R0(H,V)
Vc%
H%
R0(H,V)=1
Cumulative Number Infected (Log)
H0%, V=0
H10%, V=0
H20%, V=0
H0%, V=5E+5
H10%, V=5E+5
H20%, V=5E+5
Simu starting day
Day since 15th April 2021
R0
Vp=0%
Vp=25%
Vp=50%
Vp=75%
R0=1
Home-stay H%

Figure 30

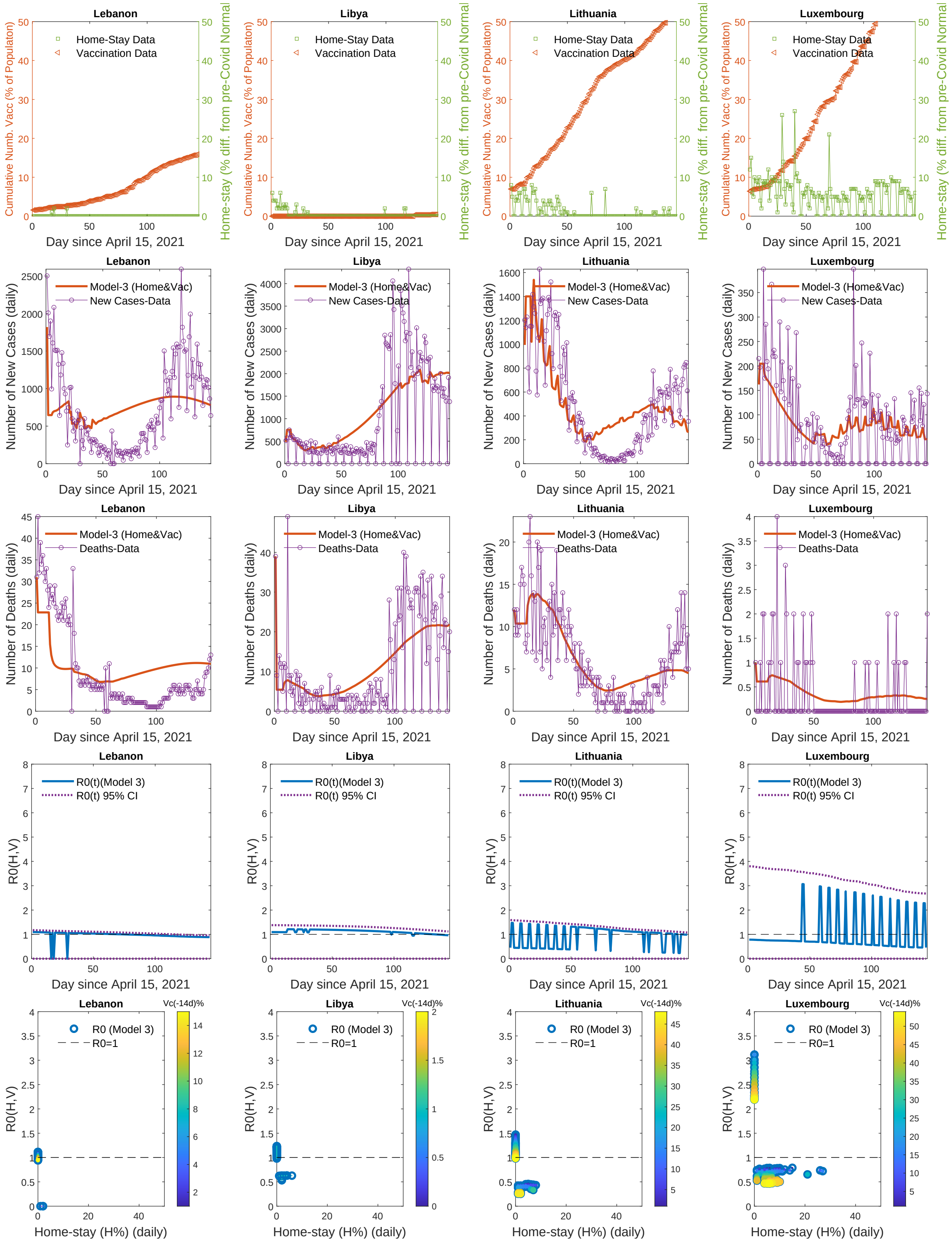

Lebanon
Libya
Lithuania
Luxembourg
Home-Stay Data
Vaccination Data
Cumulative Numb. Vacc (% of Populaton)
Home-stay (% diff. from pre-Covid Normal
Day since April 15, 2021
Model-3 (Home&Vac)
New Cases-Data
Number of New Cases (daily)
Deaths-Data
Number of Deaths (daily)
R0(t)(Model 3)
R0(t) 95% CI
R0(H,V)
R0 (Model 3)
R0=1
Vc(-14d)%
Home-stay (H%) (daily)

Figure 31

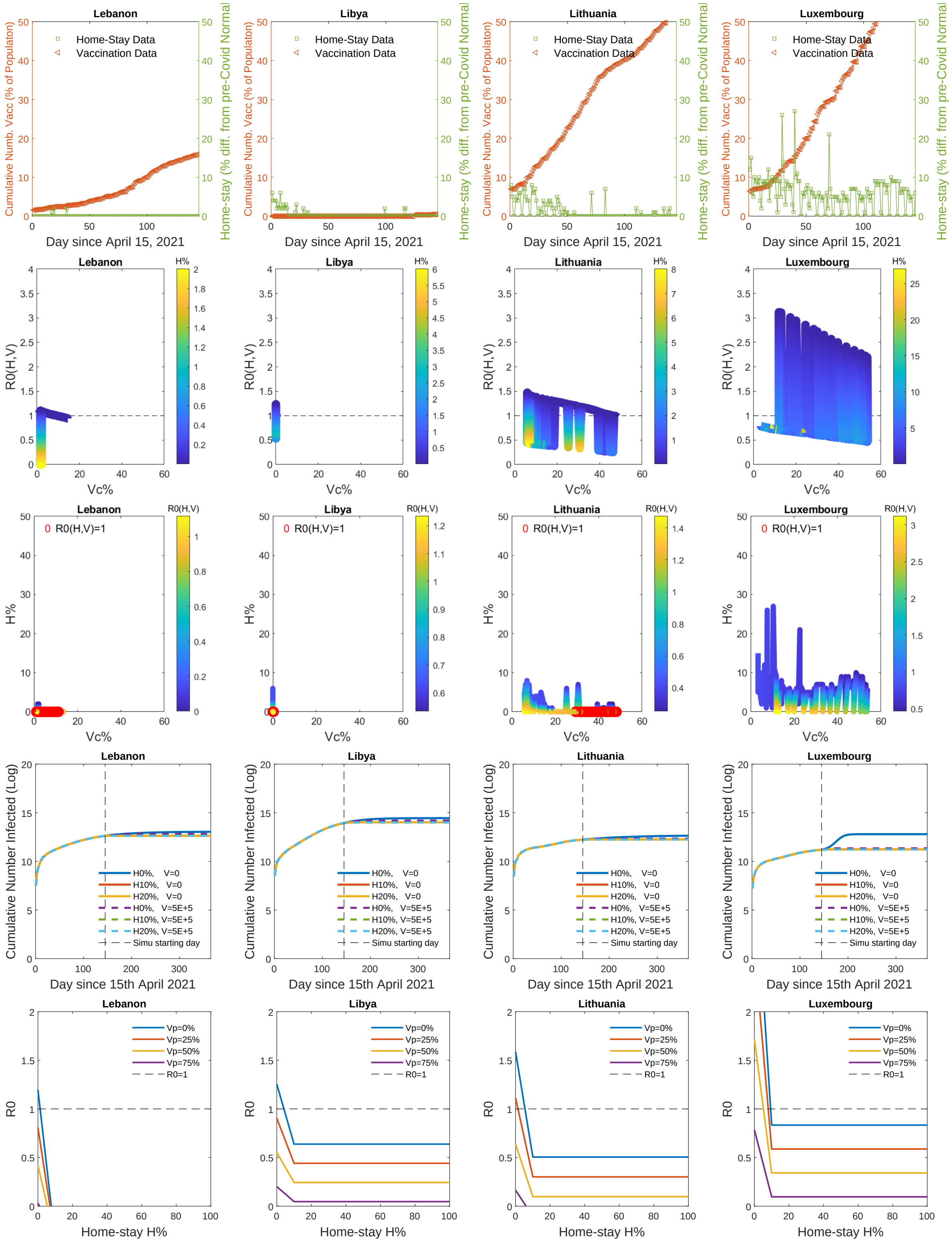
Lebanon
Libya
Lithuania
Luxembourg
Home-Stay Data
Vaccination Data
Cumulative Numb. Vacc (% of Populaton)
Home-stay (% diff. from pre-Covid Normal
Day since April 15, 2021
R0(H,V)
H%
Vc%
R0(H,V)=1
Cumulative Number Infected (Log)
H0%, V=0
H10%, V=0
H20%, V=0
H0%, V=5E+5
H10%, V=5E+5
H20%, V=5E+5
Simu starting day
Day since 15th April 2021
R0
Vp=0%
Vp=25%
Vp=50%
Vp=75%
R0=1
Home-stay H%

Figure 32

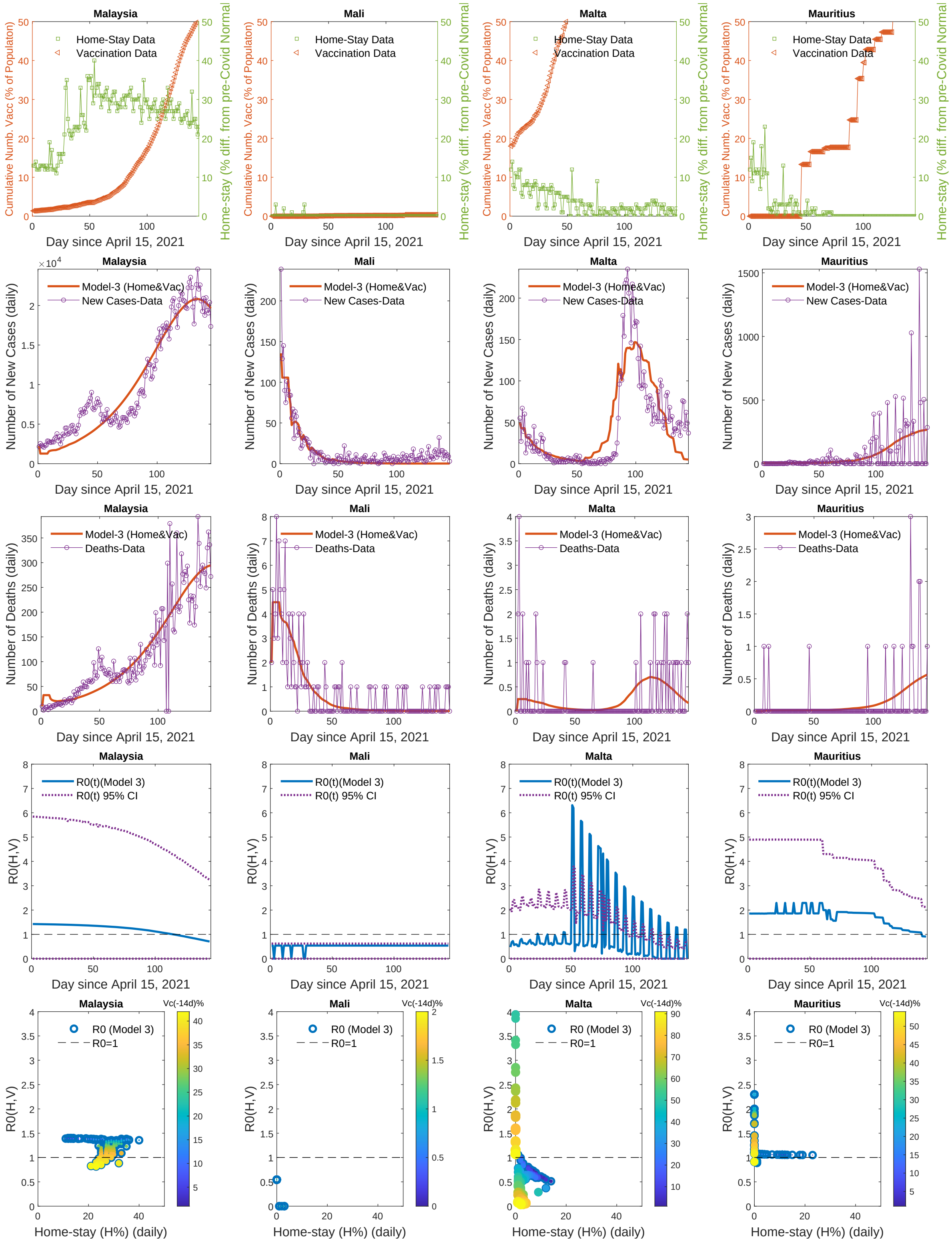
Malaysia
Mali
Malta
Mauritius
Home-Stay Data
Vaccination Data
Cumulative Numb. Vacc (% of Populaton)
Home-stay (% diff. from pre-Covid Normal
Day since April 15, 2021
Model-3 (Home&Vac)
New Cases-Data
Number of New Cases (daily)
Deaths-Data
Number of Deaths (daily)
R0(t)(Model 3)
R0(t) 95% CI
R0(H,V)
R0 (Model 3)
R0=1
Vc(-14d)%
Home-stay (H%) (daily)

Figure 33

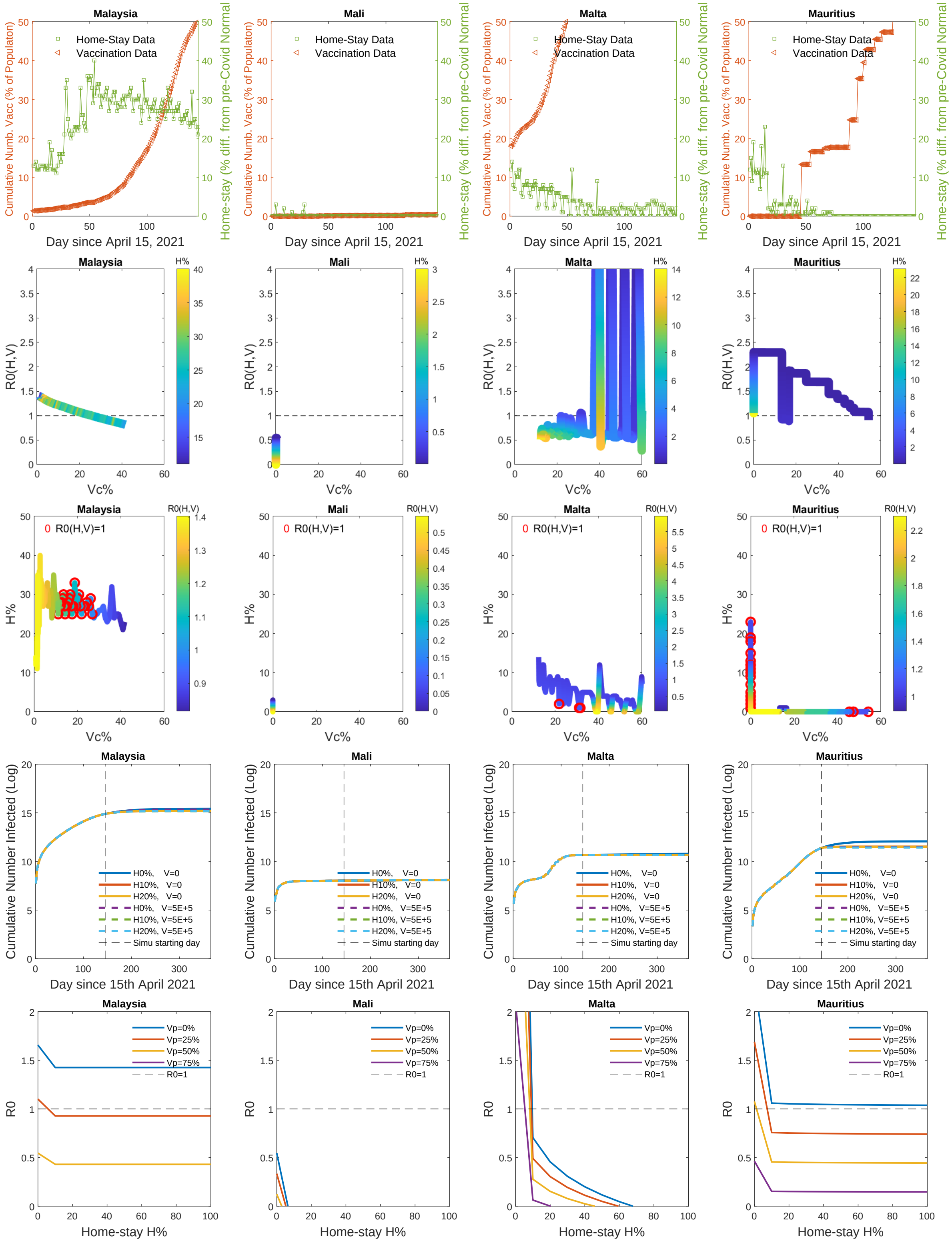
Malaysia
Mali
Malta
Mauritius
Home-Stay Data
Vaccination Data
Cumulative Numb. Vacc (% of Populaton)
Home-stay (% diff. from pre-Covid Normal
Day since April 15, 2021
R0(H,V)
Vc%
H%
0 R0(H,V)=1
Cumulative Number Infected (Log)
H0%, V=0
H10%, V=0
H20%, V=0
H0%, V=5E+5
H10%, V=5E+5
H20%, V=5E+5
Simu starting day
Day since 15th April 2021
Vp=0%
Vp=25%
Vp=50%
Vp=75%
R0=1
R0
Home-stay H%

Figure 34

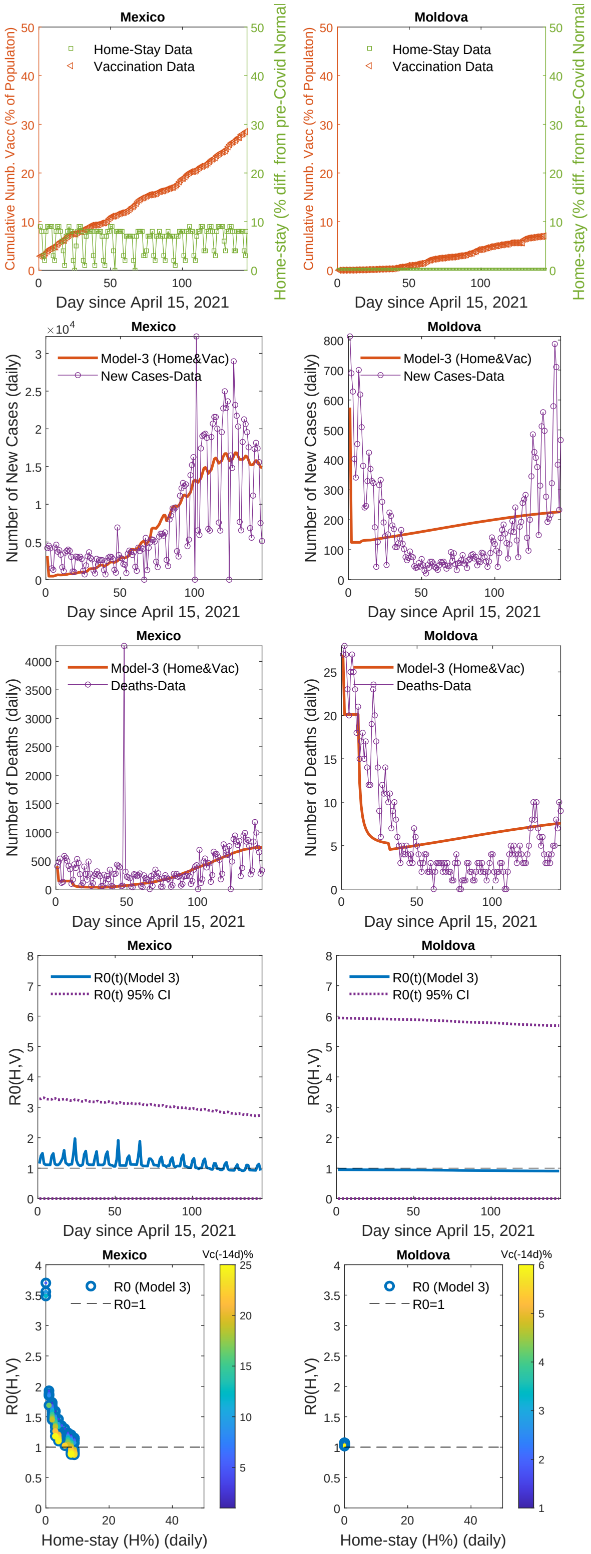
Mexico
Moldova
Home-Stay Data
Vaccination Data
Cumulative Numb. Vacc (% of Populaton)
Home-stay (% diff. from pre-Covid Normal
Day since April 15, 2021
Model-3 (Home&Vac)
New Cases-Data
Number of New Cases (daily)
Deaths-Data
Number of Deaths (daily)
R0(t)(Model 3)
R0(t) 95% CI
R0(H,V)
R0 (Model 3)
R0=1
Vc(-14d)%
Home-stay (H%) (daily)

Figure 35

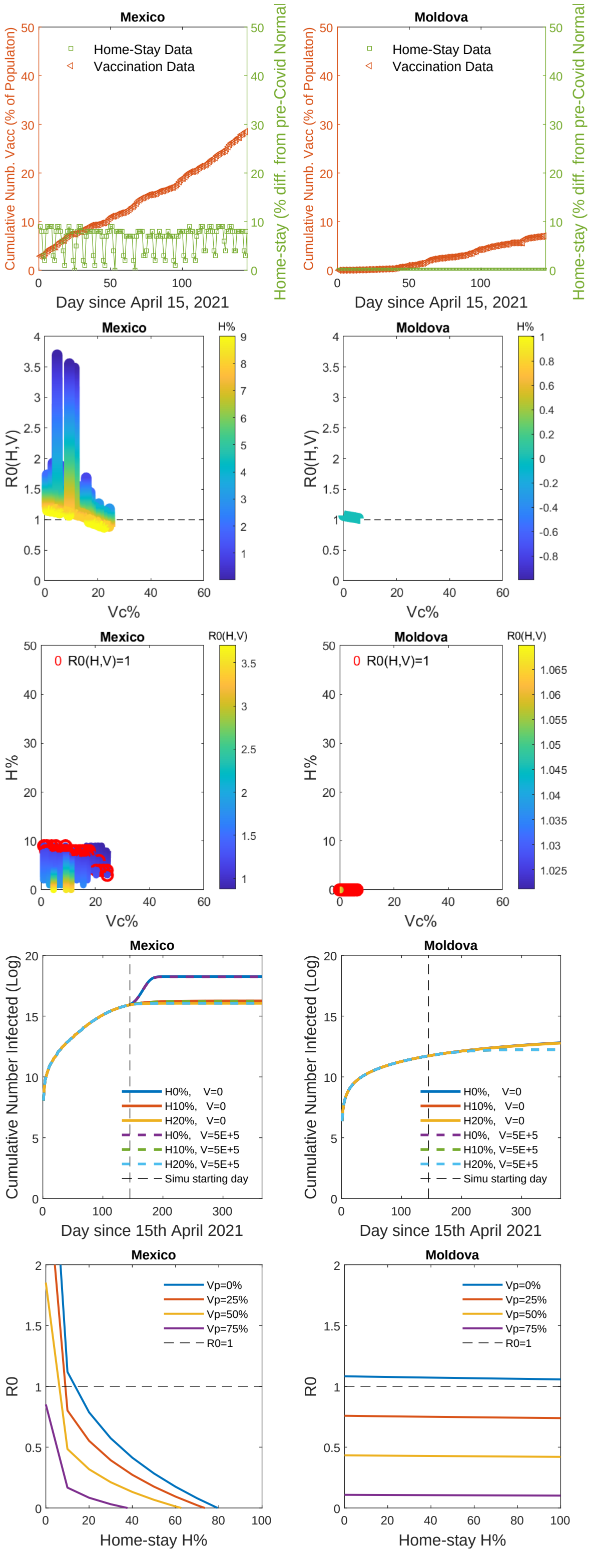
Mexico
Moldova
Home-Stay Data
Vaccination Data
Cumulative Numb. Vacc (% of Populaton)
Home-stay (% diff. from pre-Covid Normal
Day since April 15, 2021
R0(H,V)
H%
Vc%
R0(H,V)=1
Cumulative Number Infected (Log)
H0%, V=0
H10%, V=0
H20%, V=0
H0%, V=5E+5
H10%, V=5E+5
H20%, V=5E+5
Simu starting day
Day since 15th April 2021
Vp=0%
Vp=25%
Vp=50%
Vp=75%
R0=1
R0
Home-stay H%

Figure 36

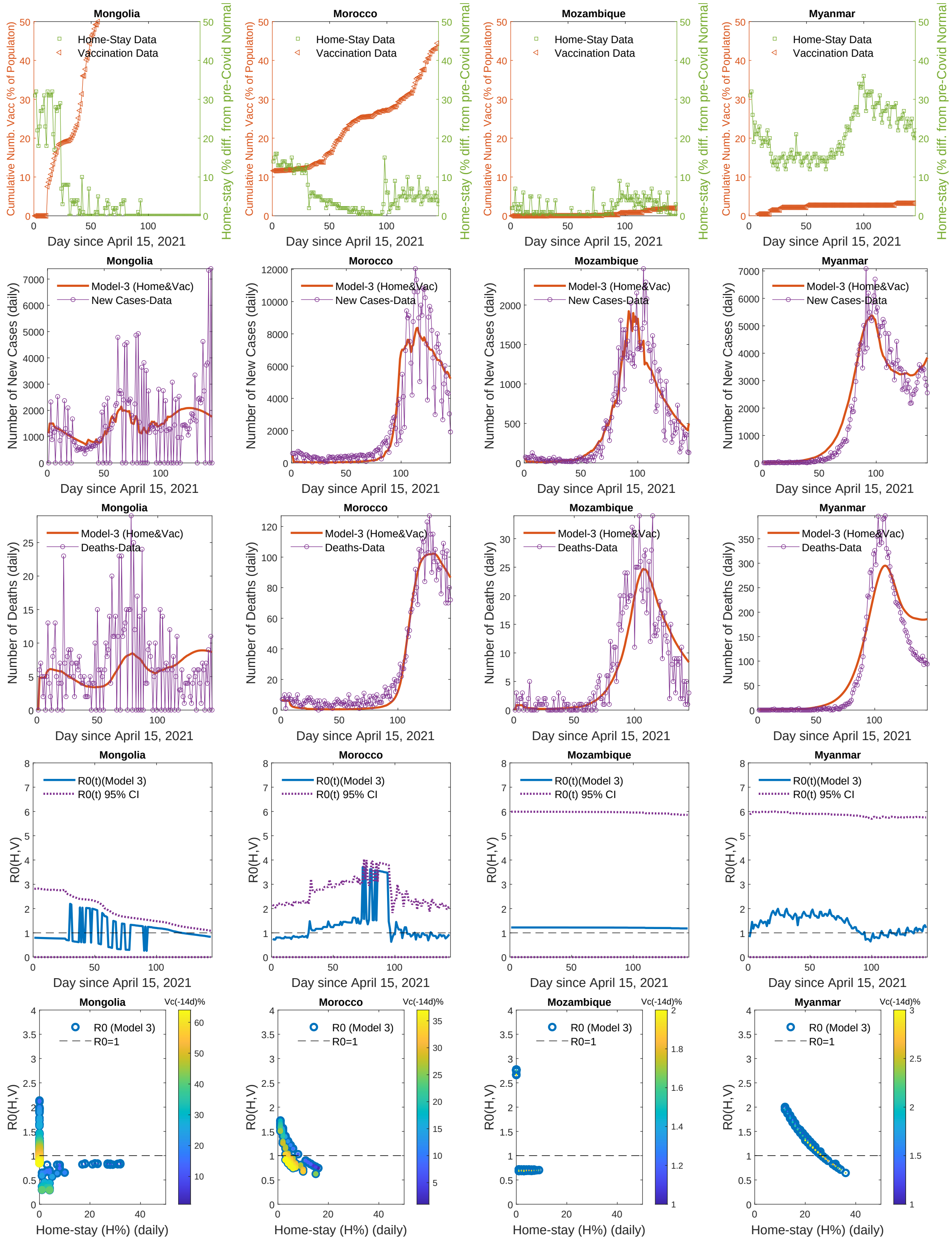
Mongolia
Morocco
Mozambique
Myanmar
Home-Stay Data
Vaccination Data
Cumulative Numb. Vacc (% of Populaton)
Home-stay (% diff. from pre-Covid Normal
Day since April 15, 2021
Model-3 (Home&Vac)
New Cases-Data
Number of New Cases (daily)
Deaths-Data
Number of Deaths (daily)
R0(t)(Model 3)
R0(t) 95% CI
R0(H,V)
R0 (Model 3)
R0=1
Vc(-14d)%
Home-stay (H%) (daily)

Figure 37

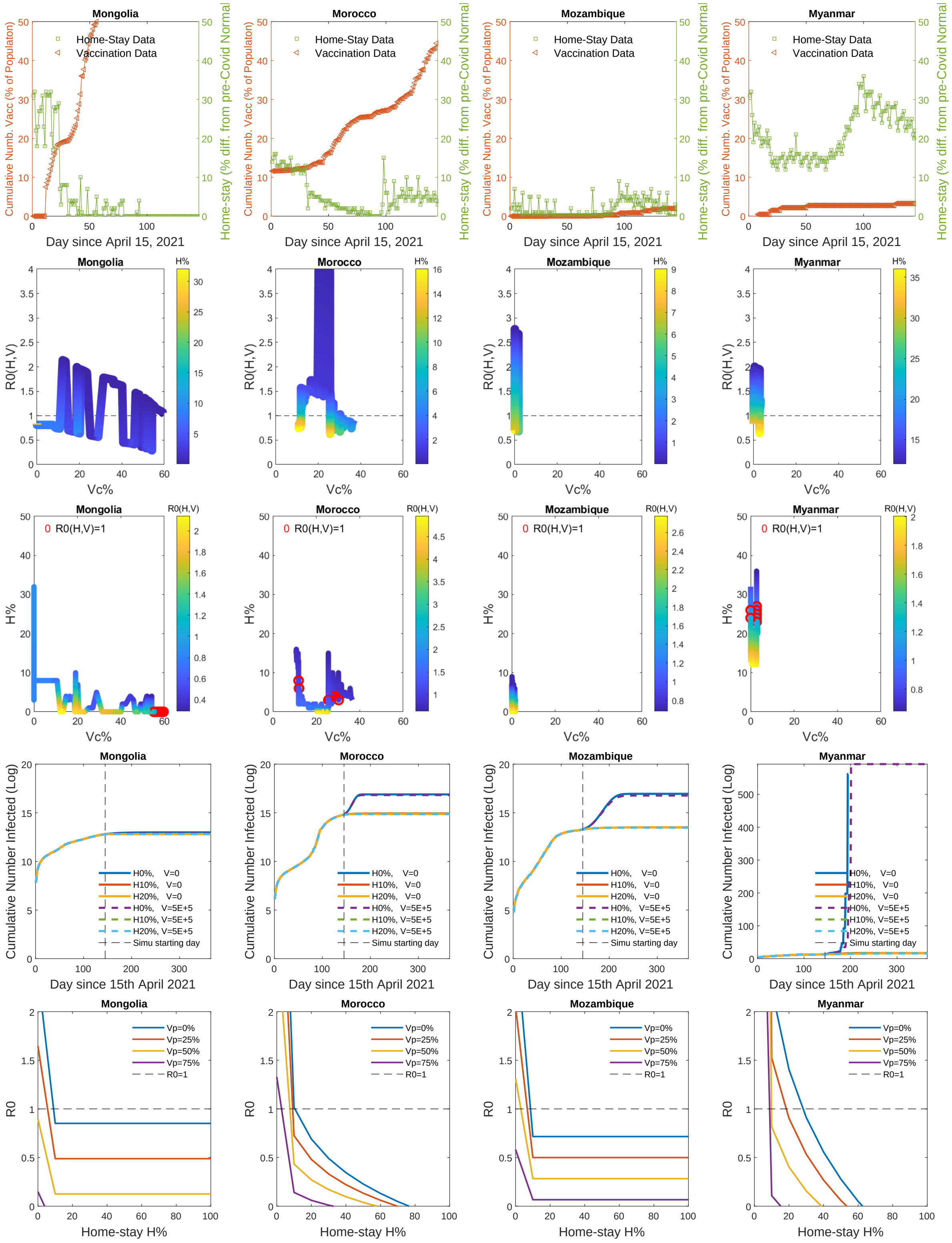
Mongolia
Morocco
Mozambique
Myanmar
Home-Stay Data
Vaccination Data
Cumulative Numb. Vacc (% of Populaton)
Home-stay (% diff. from pre-Covid Normal
Day since April 15, 2021
R0(H,V)
Vc%
H%
R0(H,V)=1
Cumulative Number Infected (Log)
H0%, V=0
H10%, V=0
H20%, V=0
H0%, V=5E+5
H10%, V=5E+5
H20%, V=5E+5
Simu starting day
Day since 15th April 2021
Vp=0%
Vp=25%
Vp=50%
Vp=75%
R0=1
R0
Home-stay H%

Figure 38

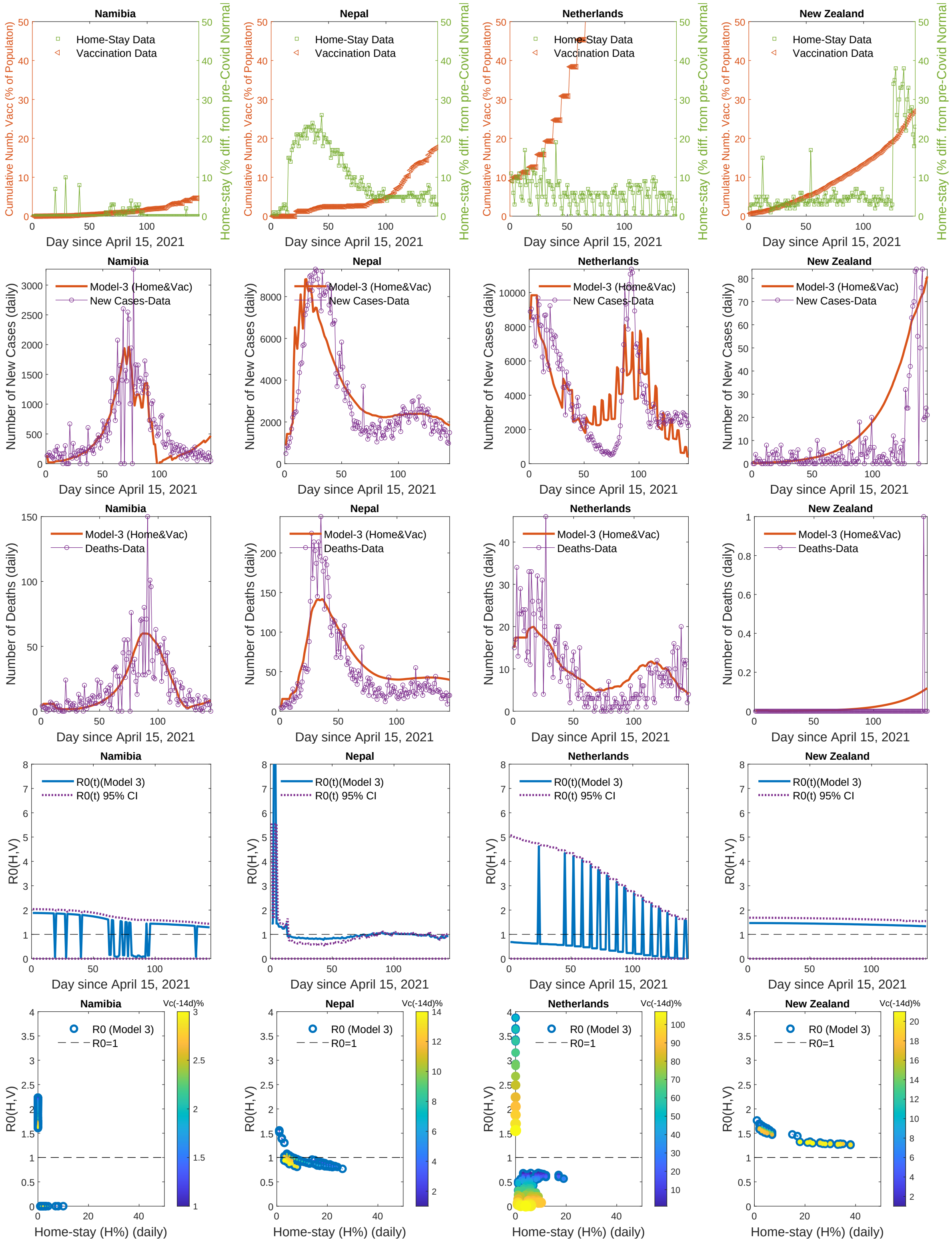
Namibia
Nepal
Netherlands
New Zealand
Home-Stay Data
Vaccination Data
Cumulative Numb. Vacc (% of Populaton)
Home-stay (% diff. from pre-Covid Normal
Day since April 15, 2021
Model-3 (Home&Vac)
New Cases-Data
Number of New Cases (daily)
Deaths-Data
Number of Deaths (daily)
R0(t)(Model 3)
R0(t) 95% CI
R0(H,V)
R0 (Model 3)
R0=1
Vc(-14d)%
Home-stay (H%) (daily)

Figure 39

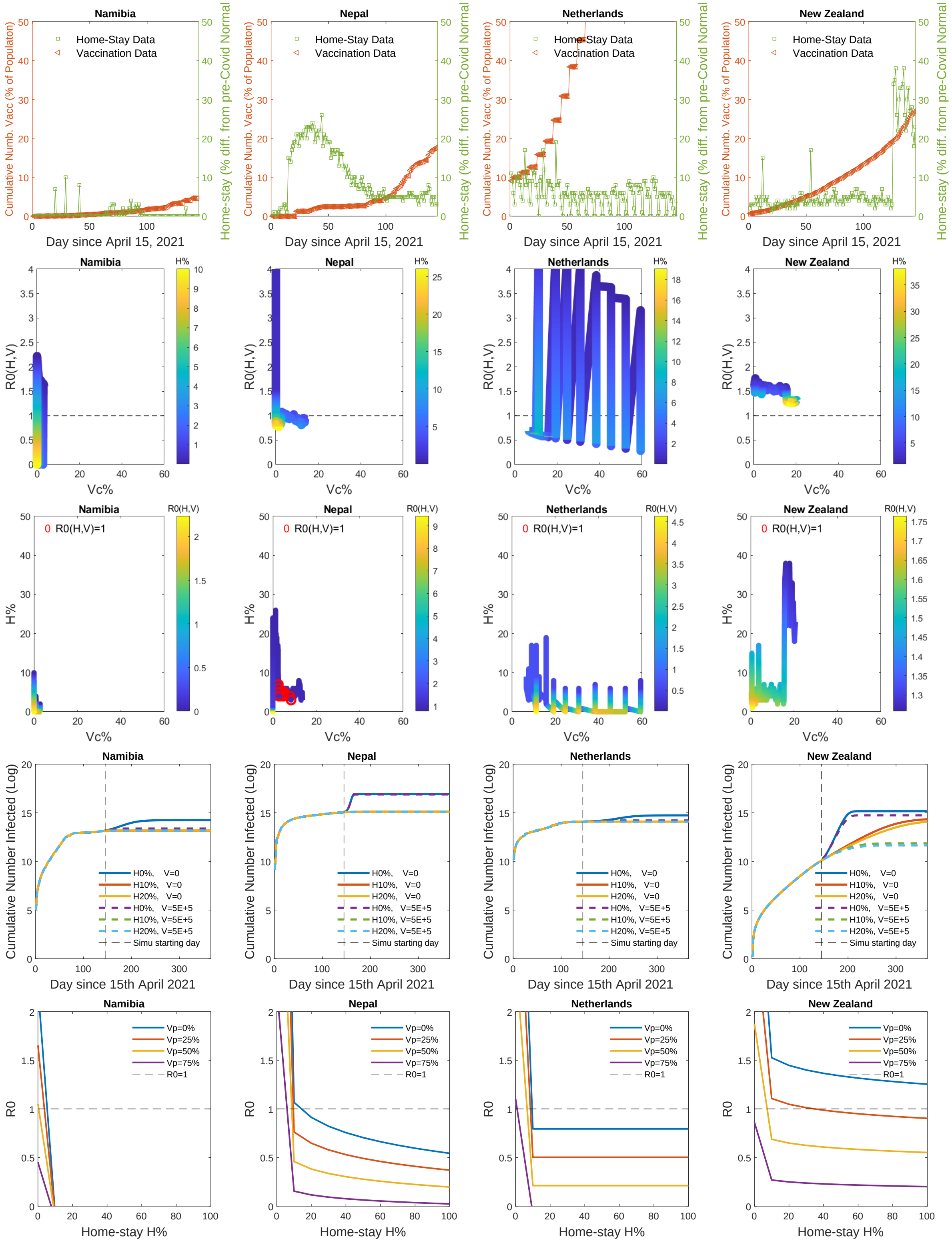
Namibia
Nepal
Netherlands
New Zealand
Home-Stay Data
Vaccination Data
Cumulative Numb. Vacc (% of Populaton)
Home-stay (% diff. from pre-Covid Normal
Day since April 15, 2021
R0(H,V)
H%
Vc%
0 R0(H,V)=1
Cumulative Number Infected (Log)
H0%, V=0
H10%, V=0
H20%, V=0
H0%, V=5E+5
H10%, V=5E+5
H20%, V=5E+5
Simu starting day
Day since 15th April 2021
R0
Vp=0%
Vp=25%
Vp=50%
Vp=75%
R0=1
Home-stay H%

Figure 40

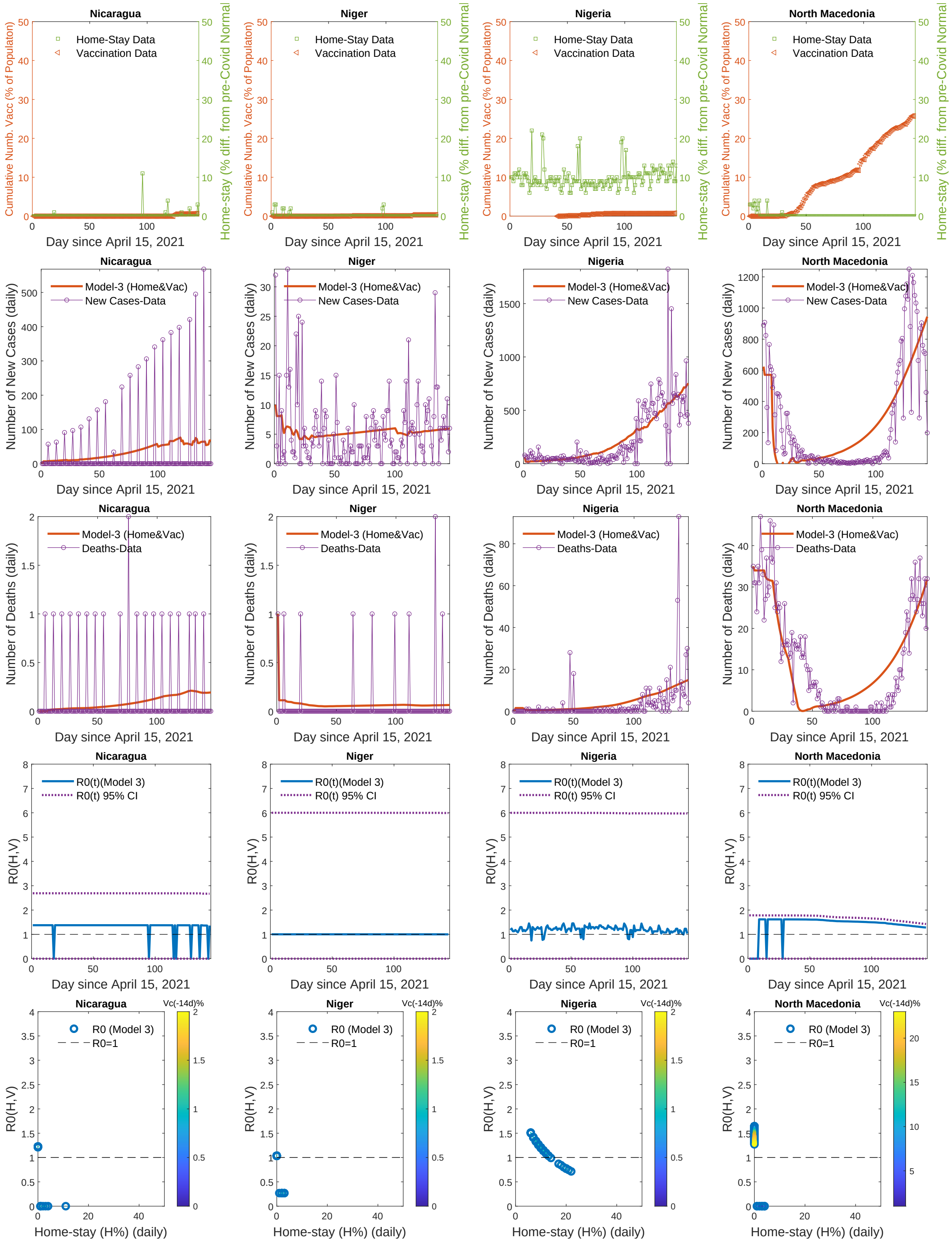

Nicaragua
Niger
Nigeria
North Macedonia
Home-Stay Data
Vaccination Data
Cumulative Numb. Vacc (% of Populaton)
Home-stay (% diff. from pre-Covid Normal
Day since April 15, 2021
Model-3 (Home&Vac)
New Cases-Data
Number of New Cases (daily)
Deaths-Data
Number of Deaths (daily)
R0(t)(Model 3)
R0(t) 95% CI
R0(H,V)
R0 (Model 3)
R0=1
Vc(-14d)%
Home-stay (H%) (daily)

Figure 41

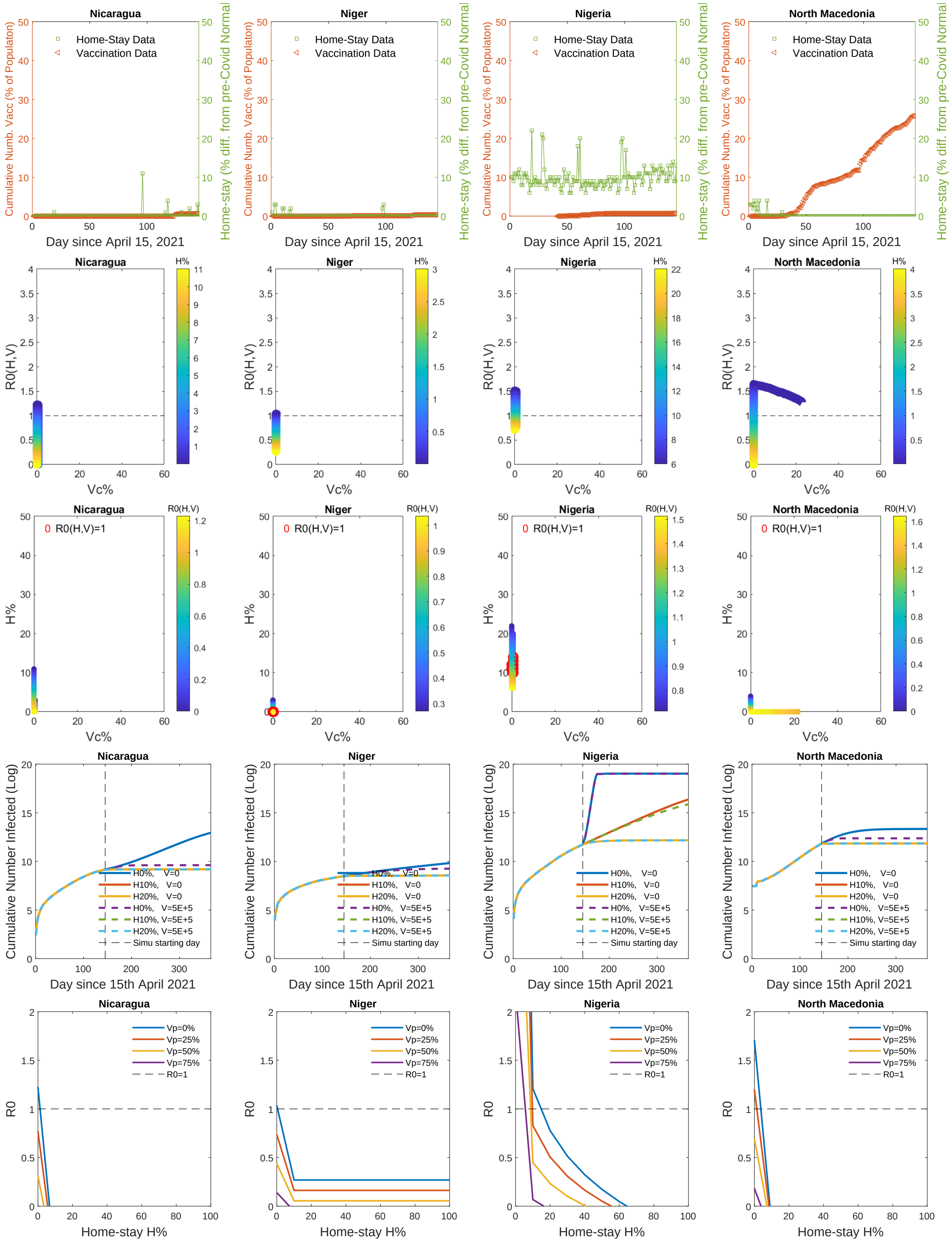
Nicaragua
Niger
Nigeria
North Macedonia
Home-Stay Data
Vaccination Data
Cumulative Numb. Vacc (% of Populaton)
Home-stay (% diff. from pre-Covid Normal
Day since April 15, 2021
R0(H,V)
Vc%
H%
R0(H,V)=1
Cumulative Number Infected (Log)
H0%, V=0
H10%, V=0
H20%, V=0
H0%, V=5E+5
H10%, V=5E+5
H20%, V=5E+5
Simu starting day
Day since 15th April 2021
R0
Vp=0%
Vp=25%
Vp=50%
Vp=75%
R0=1
Home-stay H%

Figure 42

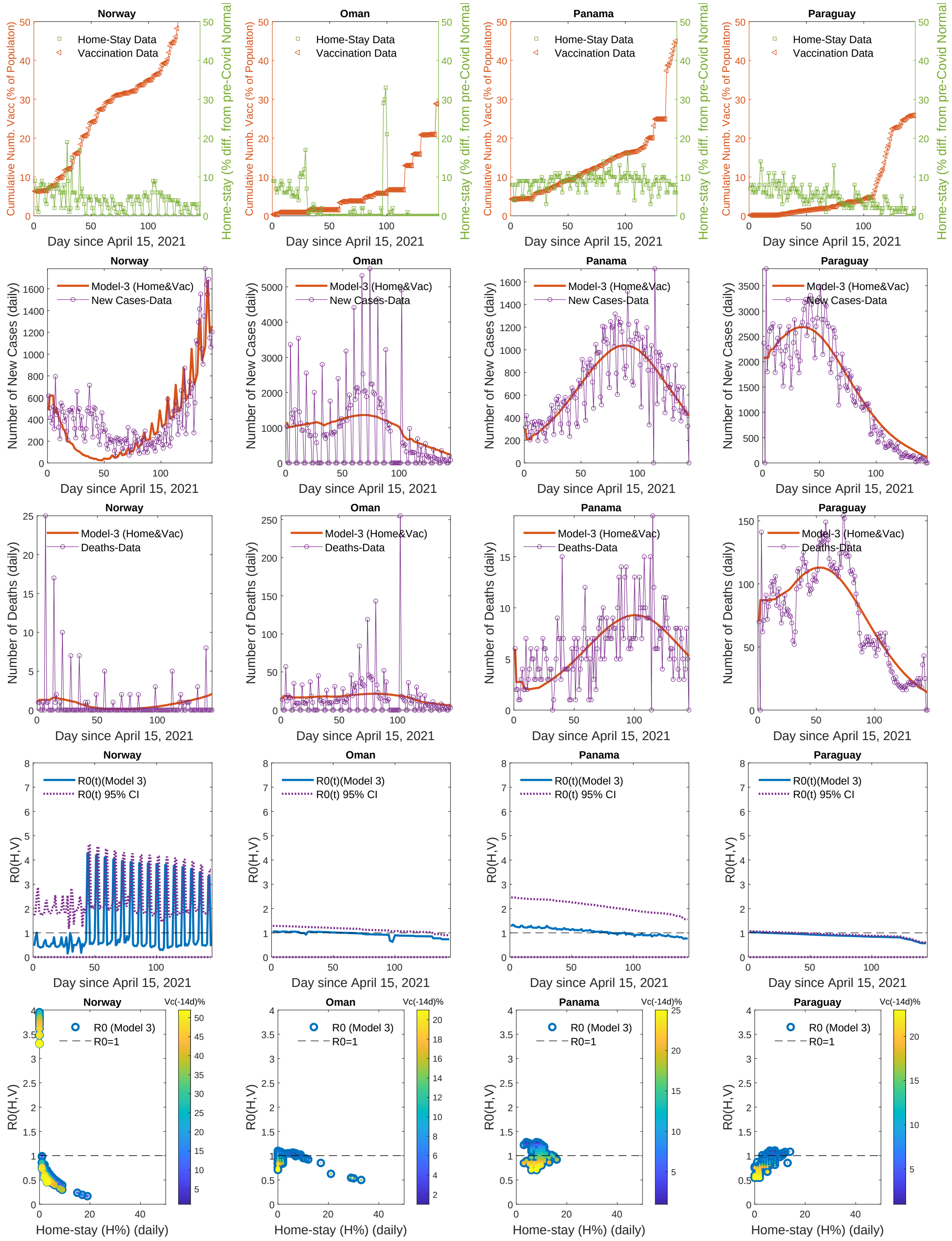

Norway
Oman
Panama
Paraguay
Home-Stay Data
Vaccination Data
Cumulative Numb. Vacc (% of Populaton)
Home-stay (% diff. from pre-Covid Normal
Day since April 15, 2021
Model-3 (Home&Vac)
New Cases-Data
Number of New Cases (daily)
Deaths-Data
Number of Deaths (daily)
R0(t)(Model 3)
R0(t) 95% CI
R0(H,V)
R0 (Model 3)
R0=1
Vc(-14d)%
Home-stay (H%) (daily)

Figure 43

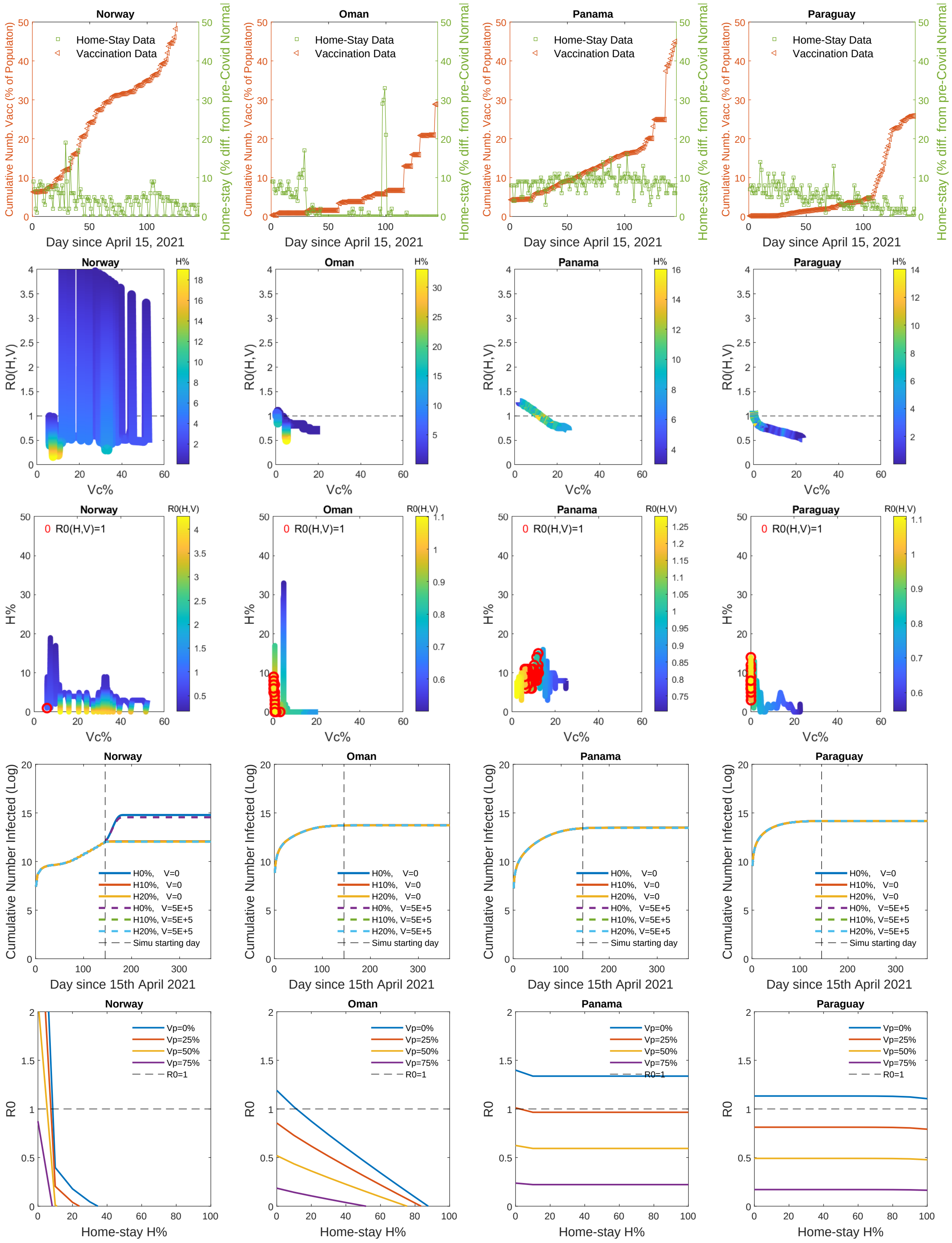
Norway
Oman
Panama
Paraguay
Home-Stay Data
Vaccination Data
Cumulative Numb. Vacc (% of Populaton)
Home-stay (% diff. from pre-Covid Normal
Day since April 15, 2021
R0(H,V)
H%
Vc%
0 R0(H,V)=1
Cumulative Number Infected (Log)
H0%, V=0
H10%, V=0
H20%, V=0
H0%, V=5E+5
H10%, V=5E+5
H20%, V=5E+5
Simu starting day
Day since 15th April 2021
R0
Vp=0%
Vp=25%
Vp=50%
Vp=75%
R0=1
Home-stay H%

Figure 44

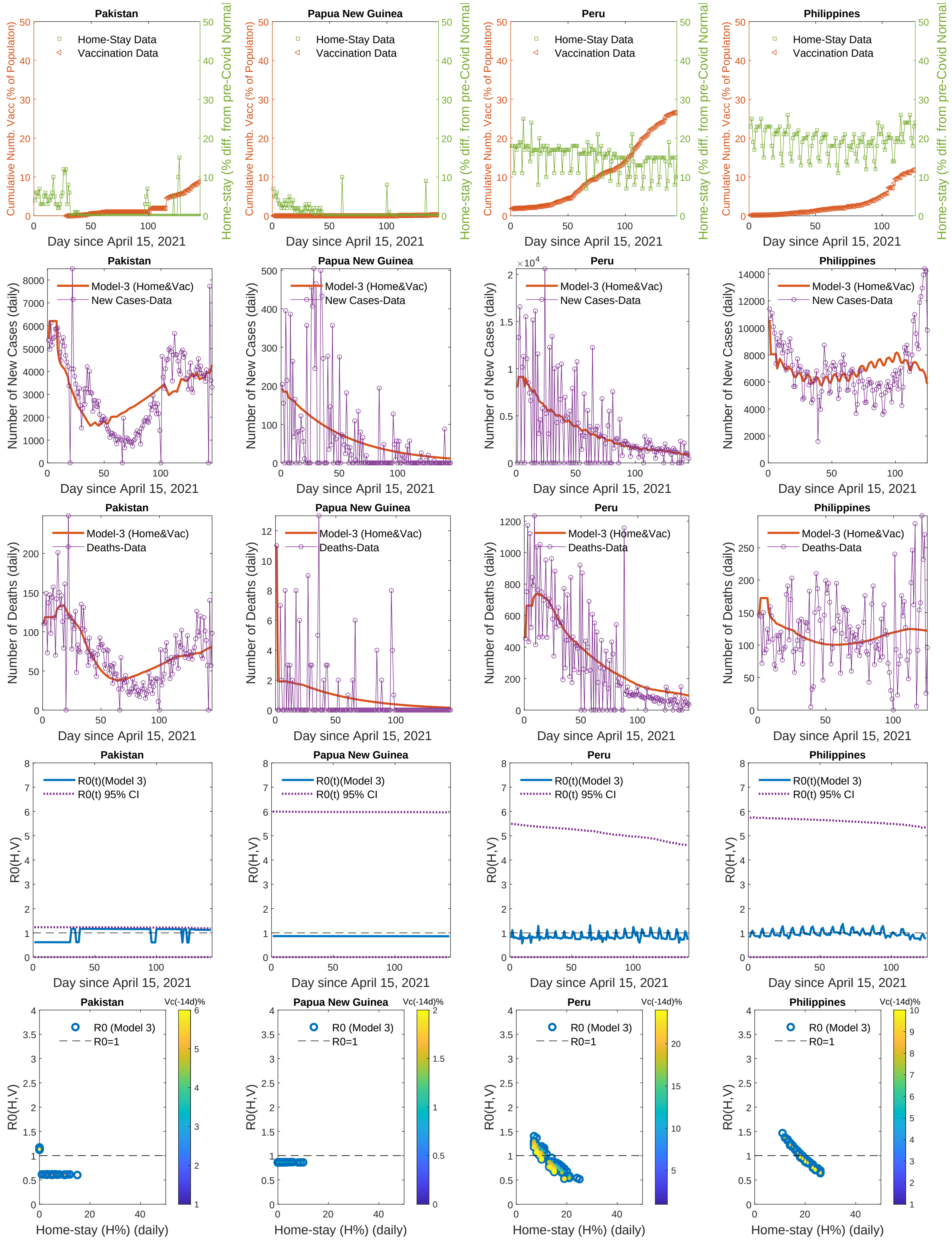
Pakistan
Papua New Guinea
Peru
Philippines
Home-Stay Data
Vaccination Data
Cumulative Numb. Vacc (% of Populaton)
Home-stay (% diff. from pre-Covid Normal
Day since April 15, 2021
Model-3 (Home&Vac)
New Cases-Data
Number of New Cases (daily)
Deaths-Data
Number of Deaths (daily)
R0(t)(Model 3)
R0(t) 95% CI
R0(H,V)
R0 (Model 3)
R0=1
Vc(-14d)%
Home-stay (H%) (daily)

Figure 45

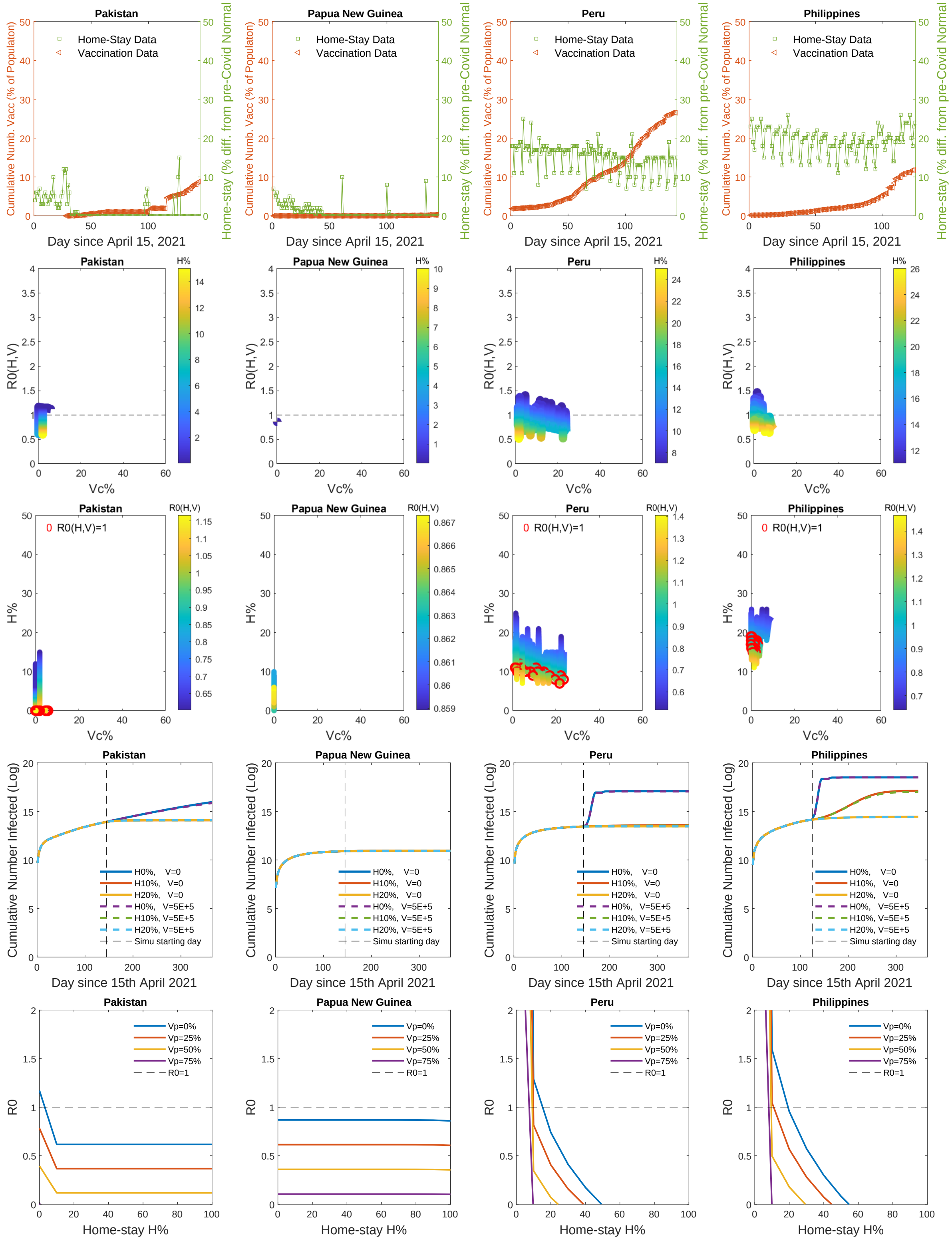

Pakistan
Papua New Guinea
Peru
Philippines
Home-Stay Data
Vaccination Data
Cumulative Numb. Vacc (% of Populaton)
Home-stay (% diff. from pre-Covid Normal
Day since April 15, 2021
R0(H,V)
H%
Vc%
R0(H,V)=1
Cumulative Number Infected (Log)
H0%, V=0
H10%, V=0
H20%, V=0
H0%, V=5E+5
H10%, V=5E+5
H20%, V=5E+5
Simu starting day
Day since 15th April 2021
R0
Vp=0%
Vp=25%
Vp=50%
Vp=75%
R0=1
Home-stay H%

Figure 46

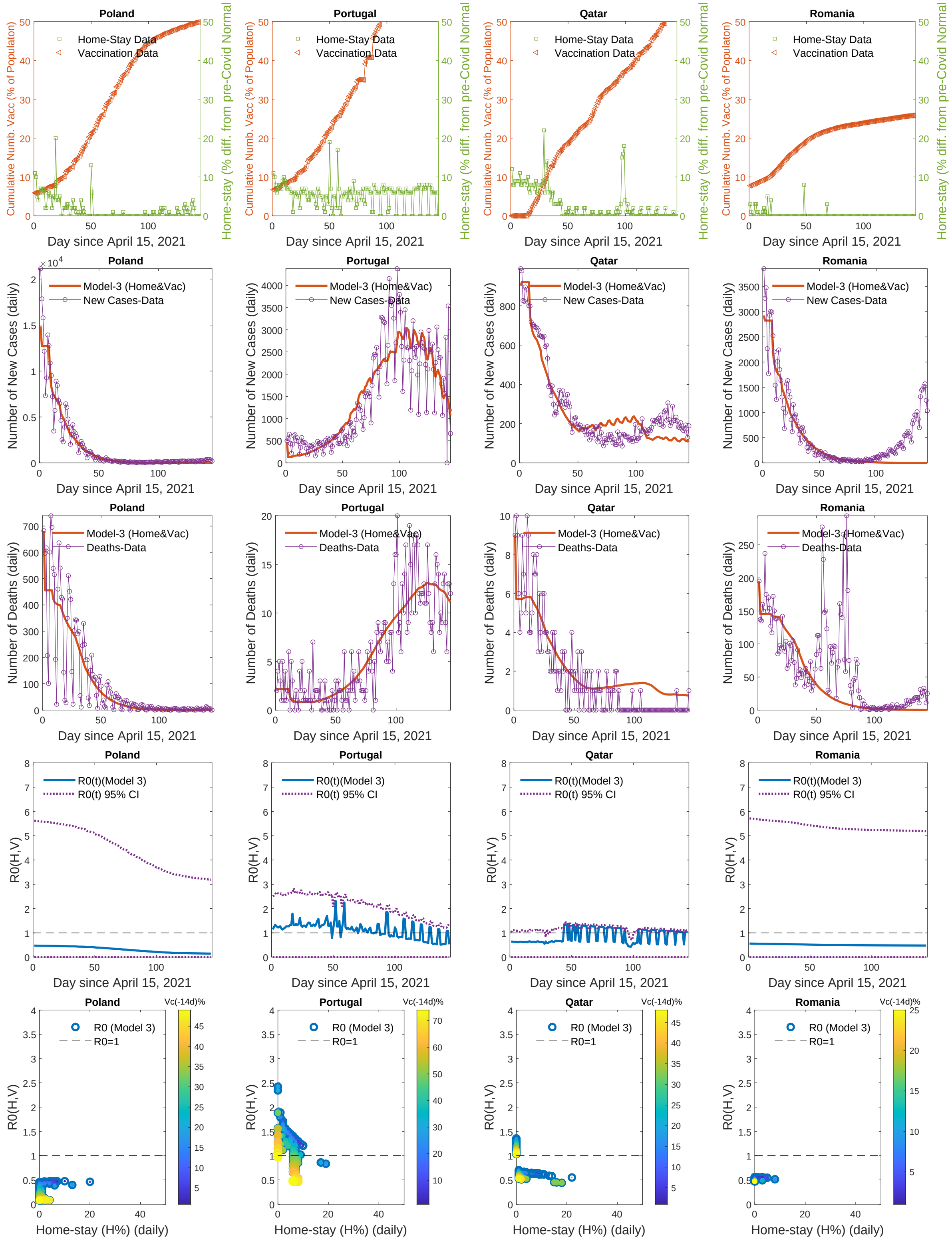
Poland
Portugal
Qatar
Romania
Home-Stay Data
Vaccination Data
Cumulative Numb. Vacc (% of Populaton)
Home-stay (% diff. from pre-Covid Normal
Day since April 15, 2021
Model-3 (Home&Vac)
New Cases-Data
Number of New Cases (daily)
Deaths-Data
Number of Deaths (daily)
R0(t)(Model 3)
R0(t) 95% CI
R0(H,V)
R0 (Model 3)
R0=1
Vc(-14d)%
Home-stay (H%) (daily)

Figure 47

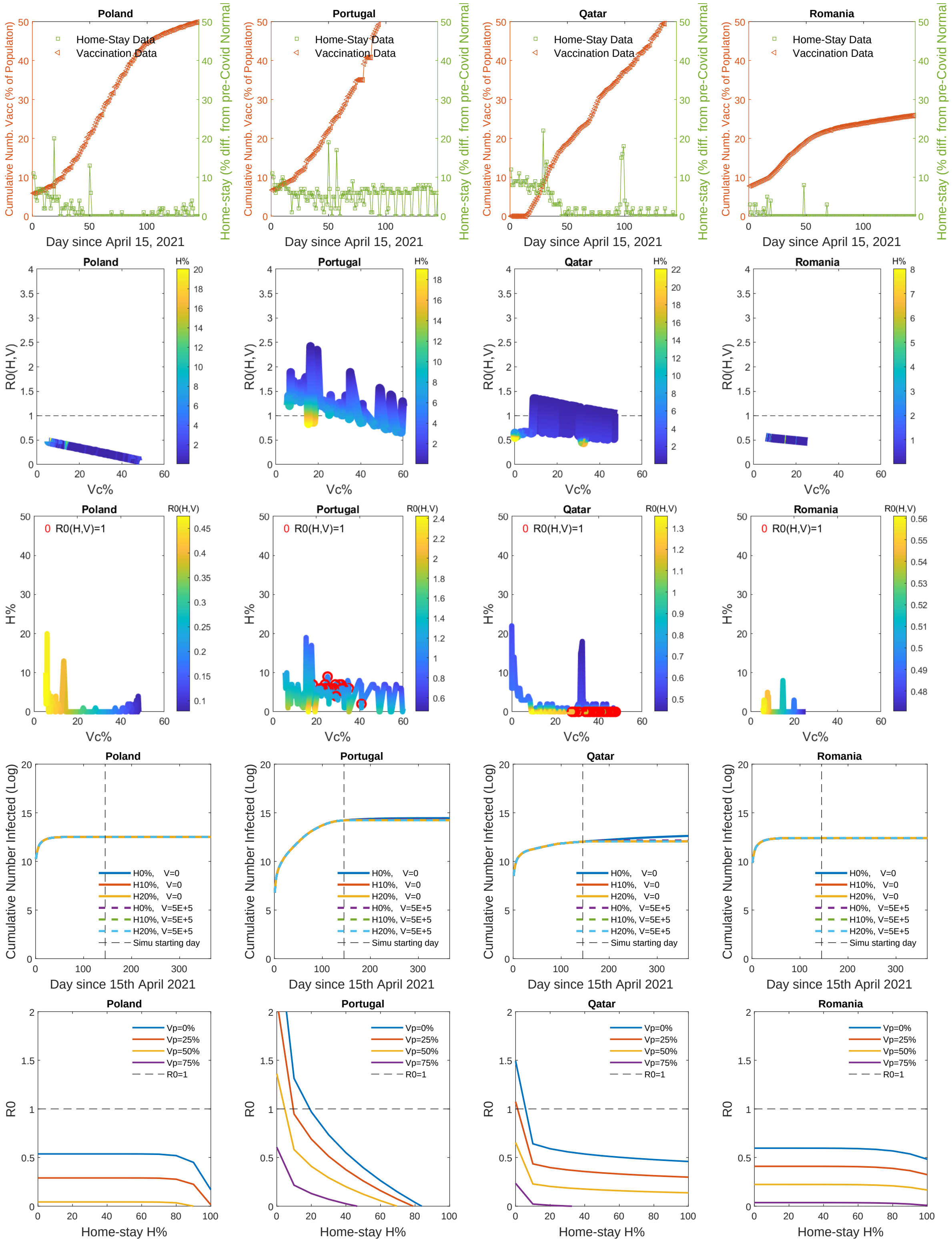
Poland
Portugal
Qatar
Romania
Home-Stay Data
Vaccination Data
Cumulative Numb. Vacc (% of Populaton)
Home-stay (% diff. from pre-Covid Normal
Day since April 15, 2021
R0(H,V)
Vc%
H%
0 R0(H,V)=1
Cumulative Number Infected (Log)
Day since 15th April 2021
H0%, V=0
H10%, V=0
H20%, V=0
H0%, V=5E+5
H10%, V=5E+5
H20%, V=5E+5
Simu starting day
R0
Home-stay H%
Vp=0%
Vp=25%
Vp=50%
Vp=75%
R0=1

Figure 48

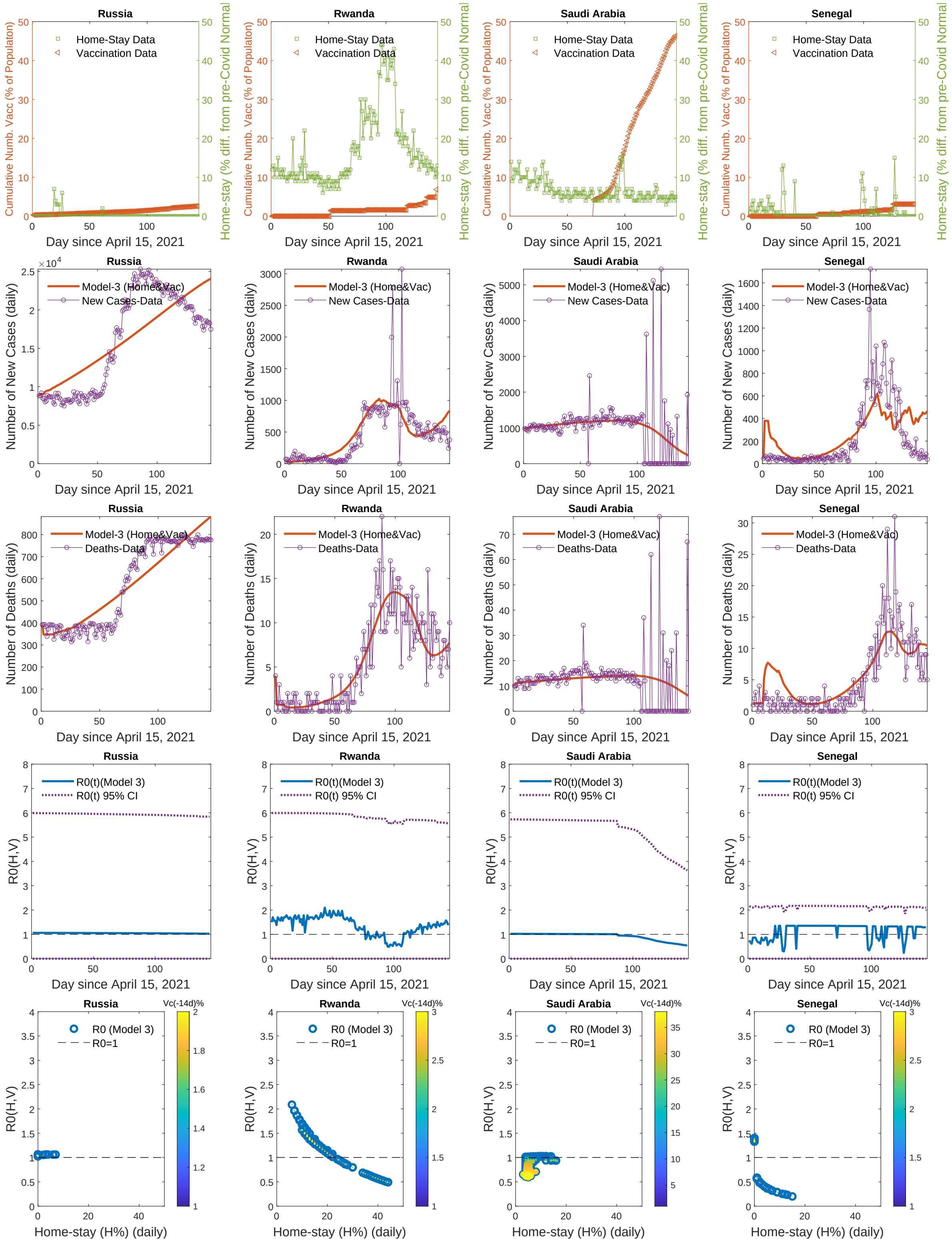
Russia
Rwanda
Saudi Arabia
Senegal
Home-Stay Data
Vaccination Data
Cumulative Numb. Vacc (% of Populaton)
Home-stay (% diff. from pre-Covid Normal
Day since April 15, 2021
Model-3 (Home&Vac)
New Cases-Data
Number of New Cases (daily)
Deaths-Data
Number of Deaths (daily)
R0(t)(Model 3)
R0(t) 95% CI
R0(H,V)
R0 (Model 3)
R0=1
Vc(-14d)%
Home-stay (H%) (daily)

Figure 49

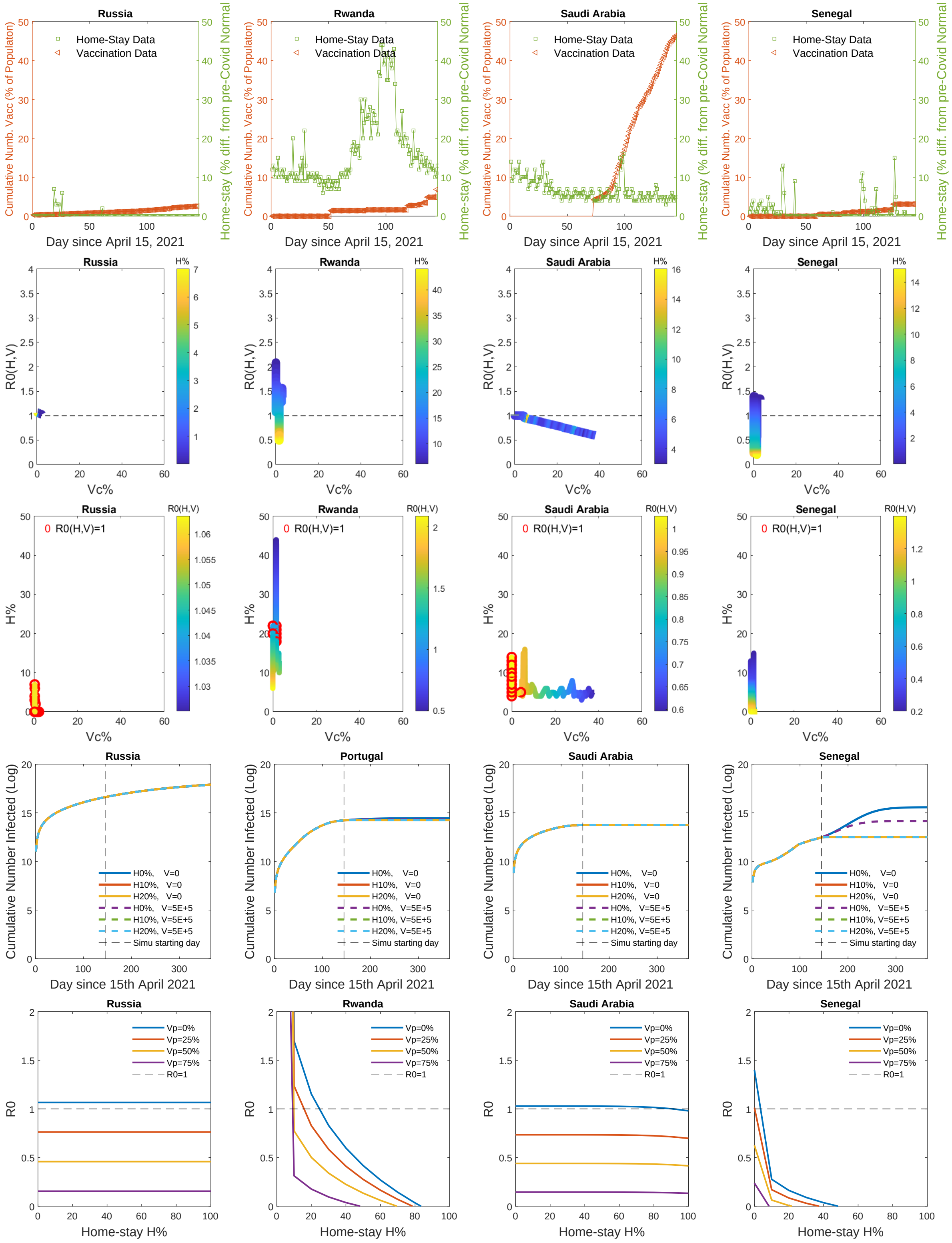
Russia
Rwanda
Saudi Arabia
Senegal
Portugal
Home-Stay Data
Vaccination Data
Cumulative Numb. Vacc (% of Populaton)
Home-stay (% diff. from pre-Covid Normal
Day since April 15, 2021
R0(H,V)
Vc%
H%
R0(H,V)=1
Cumulative Number Infected (Log)
Day since 15th April 2021
H0%, V=0
H10%, V=0
H20%, V=0
H0%, V=5E+5
H10%, V=5E+5
H20%, V=5E+5
Simu starting day
R0
Home-stay H%
Vp=0%
Vp=25%
Vp=50%
Vp=75%
R0=1

Figure 50

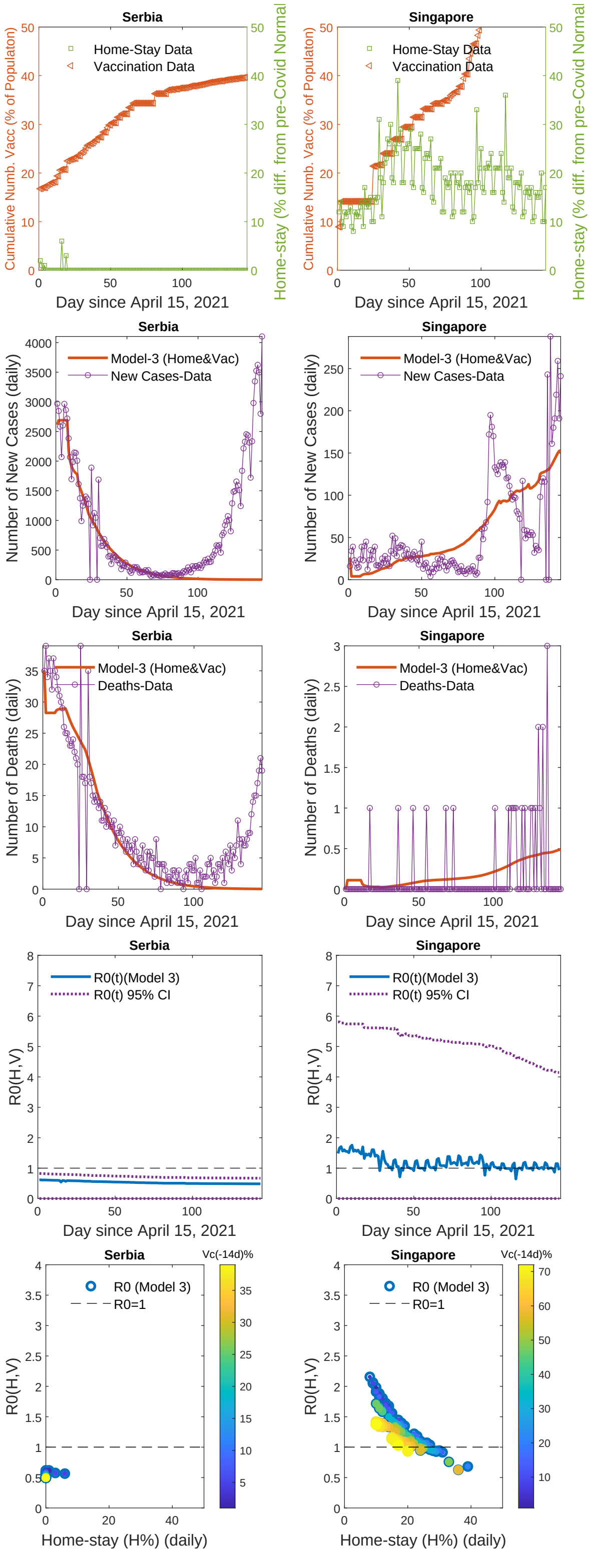

Serbia
Singapore
Home-Stay Data
Vaccination Data
Cumulative Numb. Vacc (% of Populaton)
Home-stay (% diff. from pre-Covid Normal
Day since April 15, 2021
Model-3 (Home&Vac)
New Cases-Data
Number of New Cases (daily)
Deaths-Data
Number of Deaths (daily)
R0(t)(Model 3)
R0(t) 95% CI
R0(H,V)
R0 (Model 3)
R0=1
Vc(-14d)%
Home-stay (H%) (daily)

Figure 51

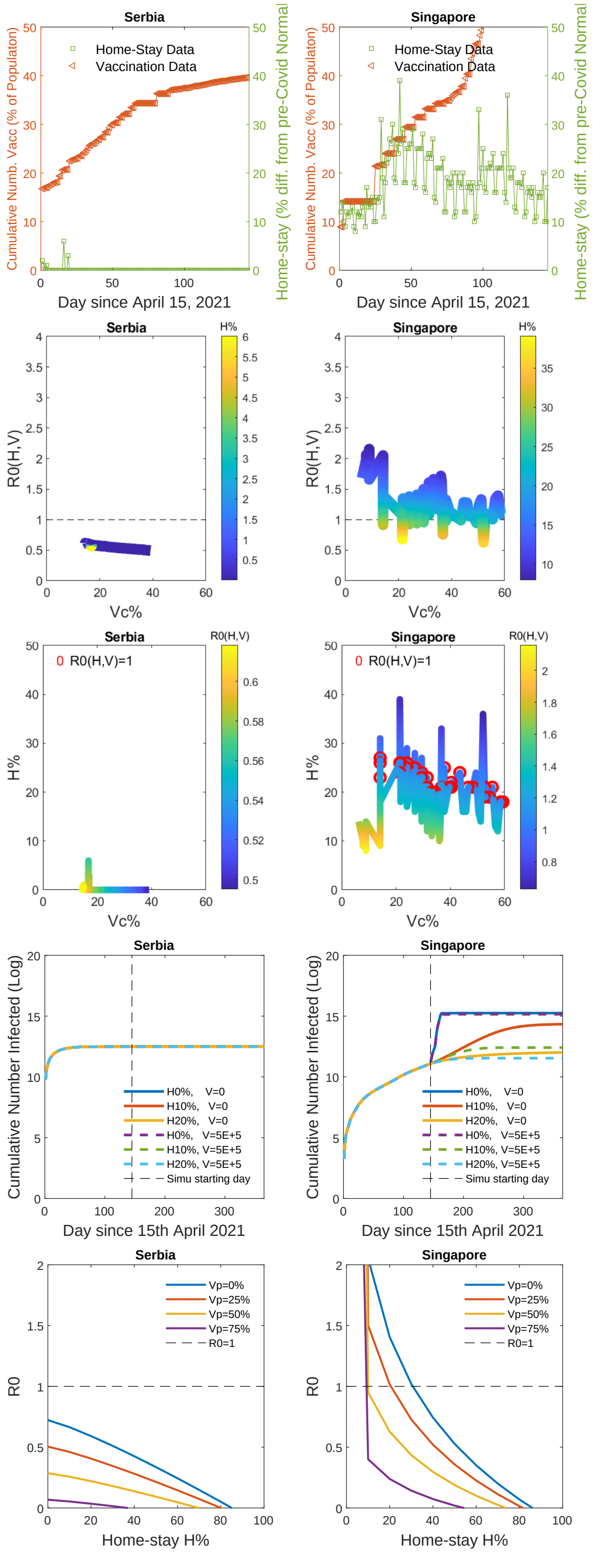
Serbia
Singapore
Home-Stay Data
Vaccination Data
Cumulative Numb. Vacc (% of Populaton)
Home-stay (% diff. from pre-Covid Normal
Day since April 15, 2021
R0(H,V)
H%
Vc%
R0(H,V)=1
Cumulative Number Infected (Log)
H0%, V=0
H10%, V=0
H20%, V=0
H0%, V=5E+5
H10%, V=5E+5
H20%, V=5E+5
Simu starting day
Day since 15th April 2021
Vp=0%
Vp=25%
Vp=50%
Vp=75%
R0=1
R0
Home-stay H%

Figure 52

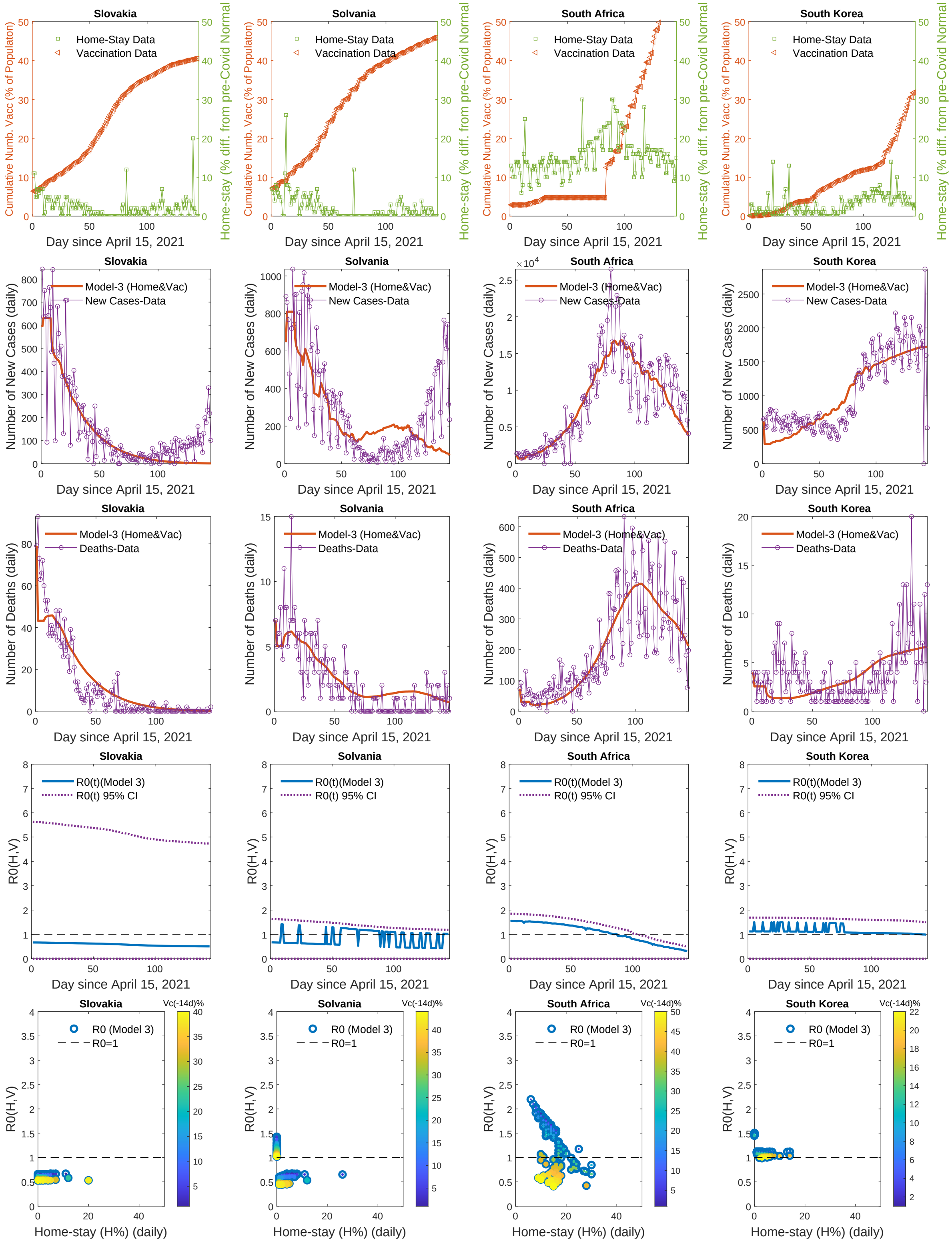
Slovakia
Solvania
South Africa
South Korea
Home-Stay Data
Vaccination Data
Cumulative Numb. Vacc (% of Populaton)
Home-stay (% diff. from pre-Covid Normal
Day since April 15, 2021
Model-3 (Home&Vac)
New Cases-Data
Number of New Cases (daily)
Deaths-Data
Number of Deaths (daily)
R0(t)(Model 3)
R0(t) 95% CI
R0(H,V)
R0 (Model 3)
R0=1
Vc(-14d)%
Home-stay (H%) (daily)

Figure 53

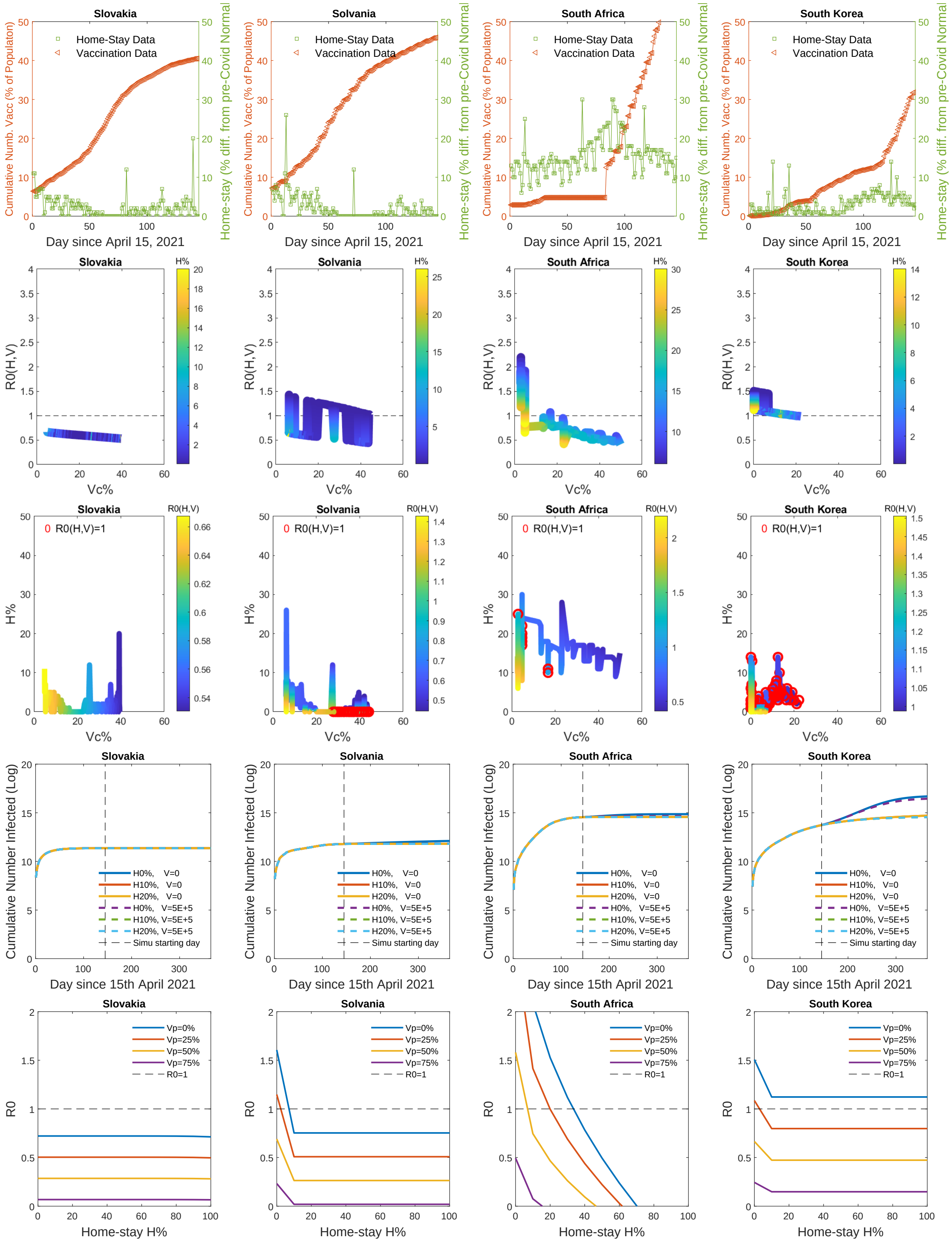
Slovakia
Solvania
South Africa
South Korea
Home-Stay Data
Vaccination Data
Cumulative Numb. Vacc (% of Populaton)
Home-stay (% diff. from pre-Covid Normal
Day since April 15, 2021
R0(H,V)
H%
Vc%
0 R0(H,V)=1
Cumulative Number Infected (Log)
H0%, V=0
H10%, V=0
H20%, V=0
H0%, V=5E+5
H10%, V=5E+5
H20%, V=5E+5
Simu starting day
Day since 15th April 2021
R0
Vp=0%
Vp=25%
Vp=50%
Vp=75%
R0=1
Home-stay H%

Figure 54

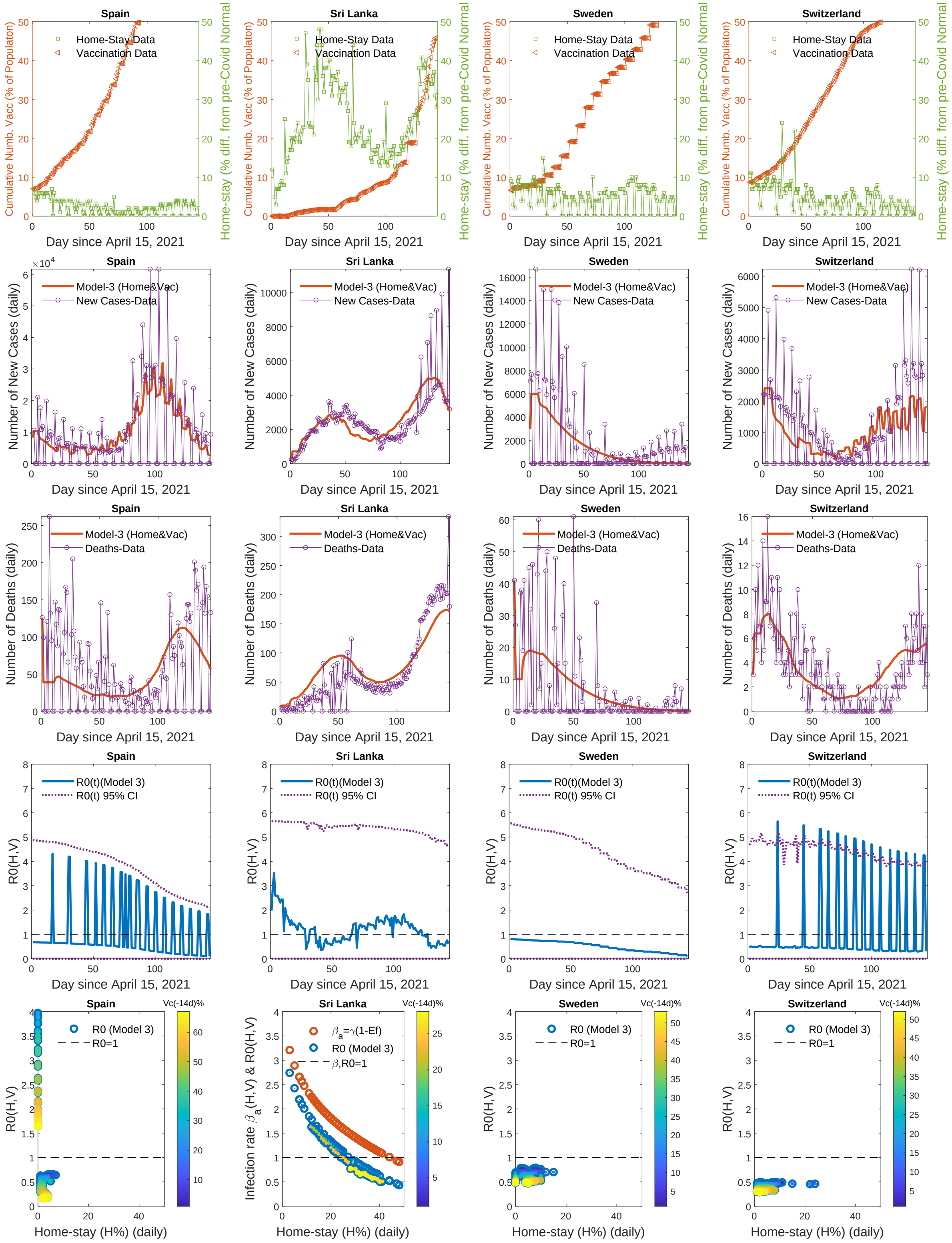
Spain
Sri Lanka
Sweden
Switzerland
Home-Stay Data
Vaccination Data
Cumulative Numb. Vacc (% of Populaton)
Home-stay (% diff. from pre-Covid Normal
Day since April 15, 2021
Model-3 (Home&Vac)
New Cases-Data
Number of New Cases (daily)
Deaths-Data
Number of Deaths (daily)
R0(t)(Model 3)
R0(t) 95% CI
R0(H,V)
R0 (Model 3)
R0=1
Vc(-14d)%
β_a=γ(1-Ef)
β,R0=1
Infection rate β_a(H,V) & R0(H,V)
Home-stay (H%) (daily)

Figure 55

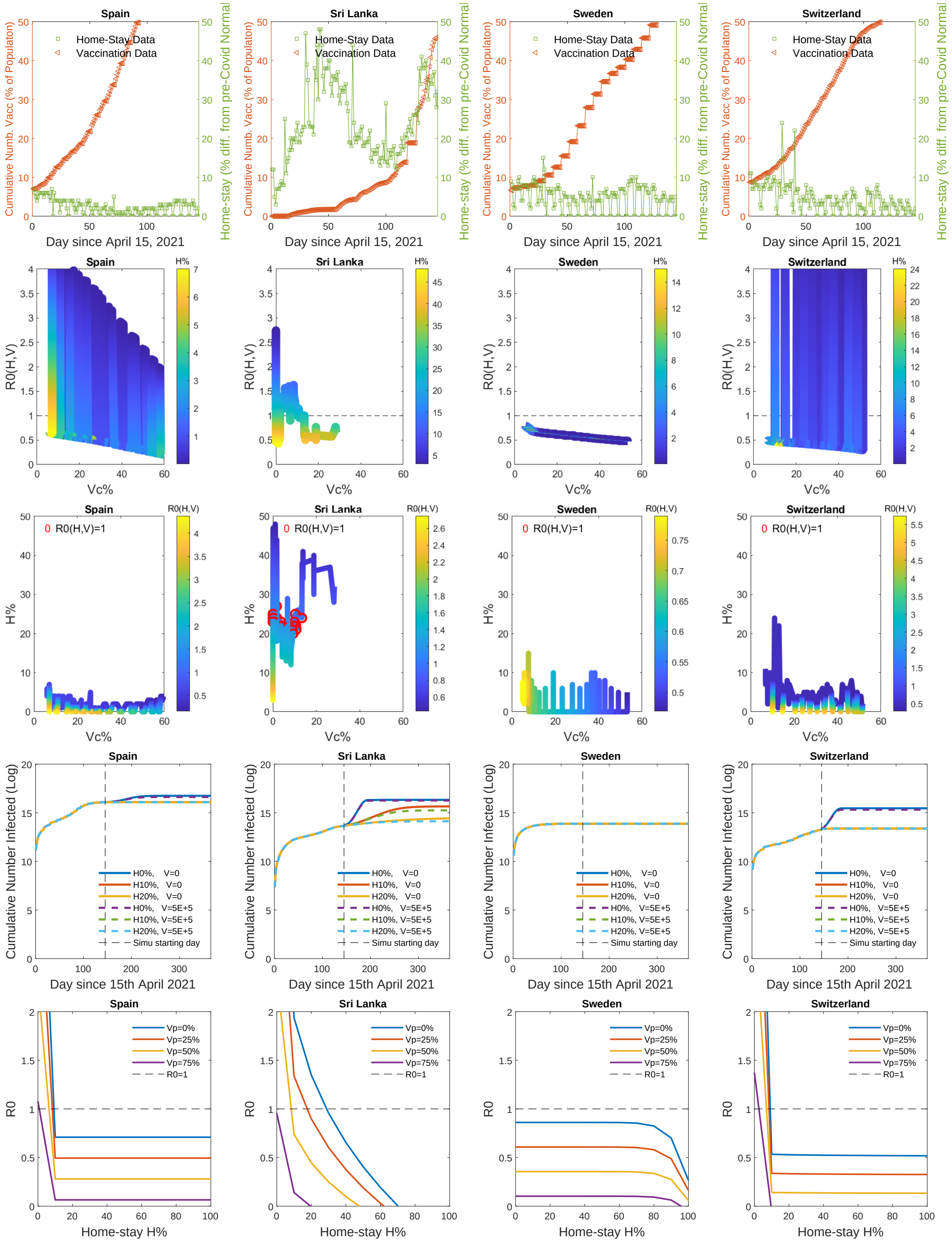
Spain
Sri Lanka
Sweden
Switzerland
Home-Stay Data
Vaccination Data
Cumulative Numb. Vacc (% of Populaton)
Home-stay (% diff. from pre-Covid Normal
Day since April 15, 2021
R0(H,V)
H%
Vc%
0 R0(H,V)=1
Cumulative Number Infected (Log)
H0%, V=0
H10%, V=0
H20%, V=0
H0%, V=5E+5
H10%, V=5E+5
H20%, V=5E+5
Simu starting day
Day since 15th April 2021
R0
Vp=0%
Vp=25%
Vp=50%
Vp=75%
R0=1
Home-stay H%

Figure 56

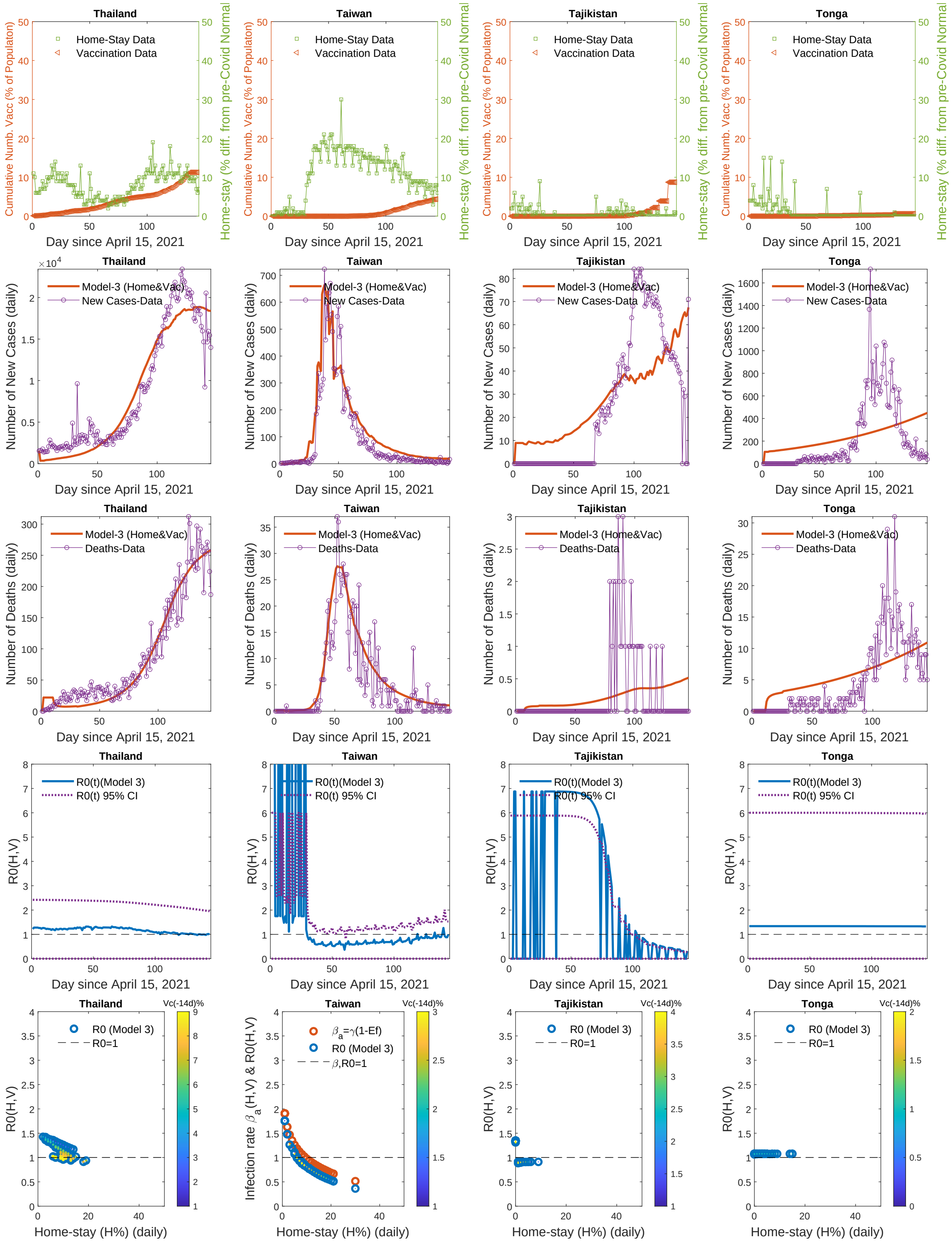
Thailand
Taiwan
Tajikistan
Tonga
Home-Stay Data
Vaccination Data
Cumulative Numb. Vacc (% of Populaton)
Home-stay (% diff. from pre-Covid Normal
Day since April 15, 2021
Model-3 (Home&Vac)
New Cases-Data
Number of New Cases (daily)
Deaths-Data
Number of Deaths (daily)
R0(t)(Model 3)
R0(t) 95% CI
R0(H,V)
R0 (Model 3)
R0=1
Vc(-14d)%
Infection rate β_a(H,V) & R0(H,V)
β_a=γ(1-Ef)
β,R0=1
Home-stay (H%) (daily)

Figure 57

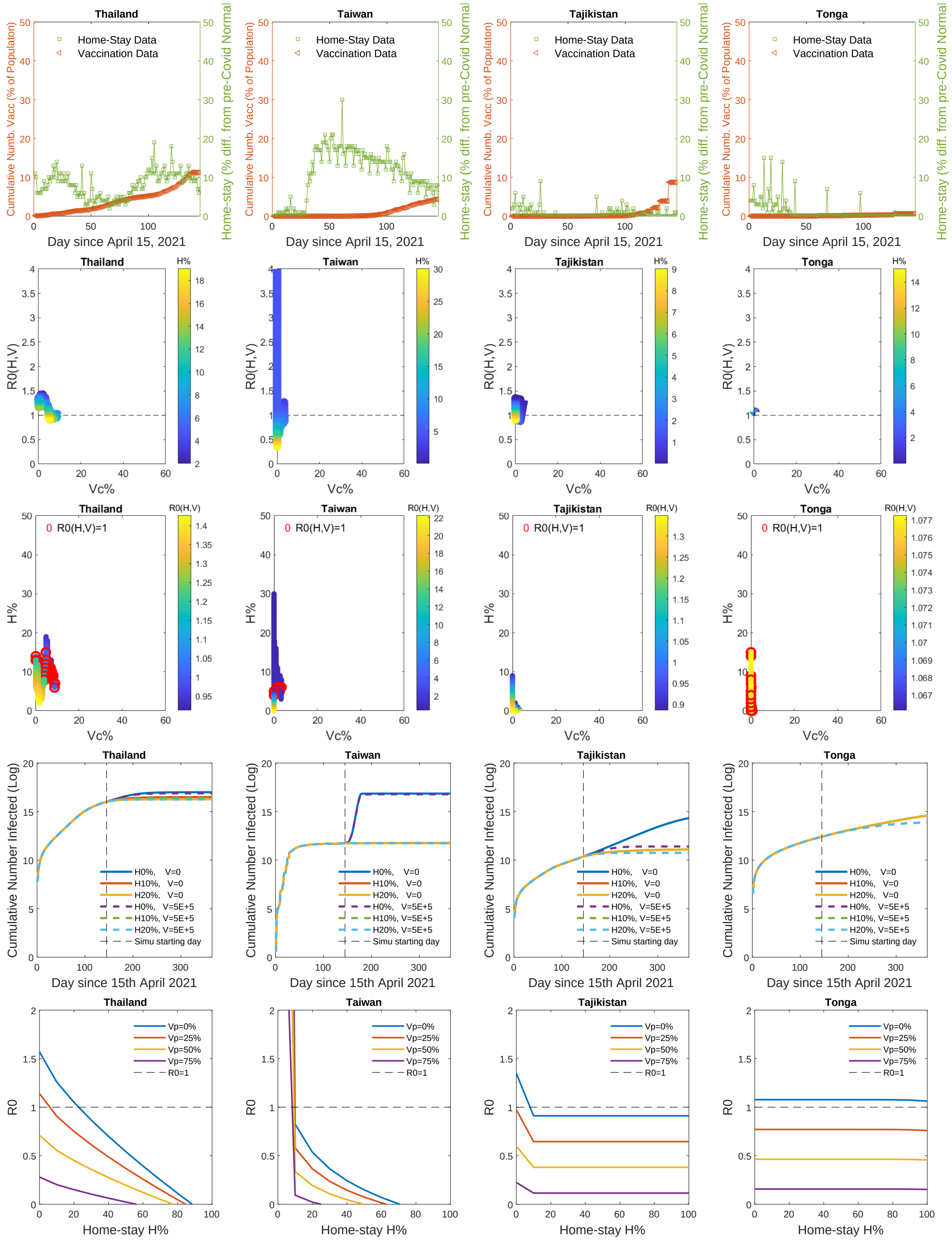

Thailand
Taiwan
Tajikistan
Tonga
Home-Stay Data
Vaccination Data
Cumulative Numb. Vacc (% of Populaton)
Home-stay (% diff. from pre-Covid Normal
Day since April 15, 2021
R0(H,V)
H%
Vc%
R0(H,V)=1
Cumulative Number Infected (Log)
H0%, V=0
H10%, V=0
H20%, V=0
H0%, V=5E+5
H10%, V=5E+5
H20%, V=5E+5
Simu starting day
Day since 15th April 2021
R0
Vp=0%
Vp=25%
Vp=50%
Vp=75%
R0=1
Home-stay H%

Figure 58

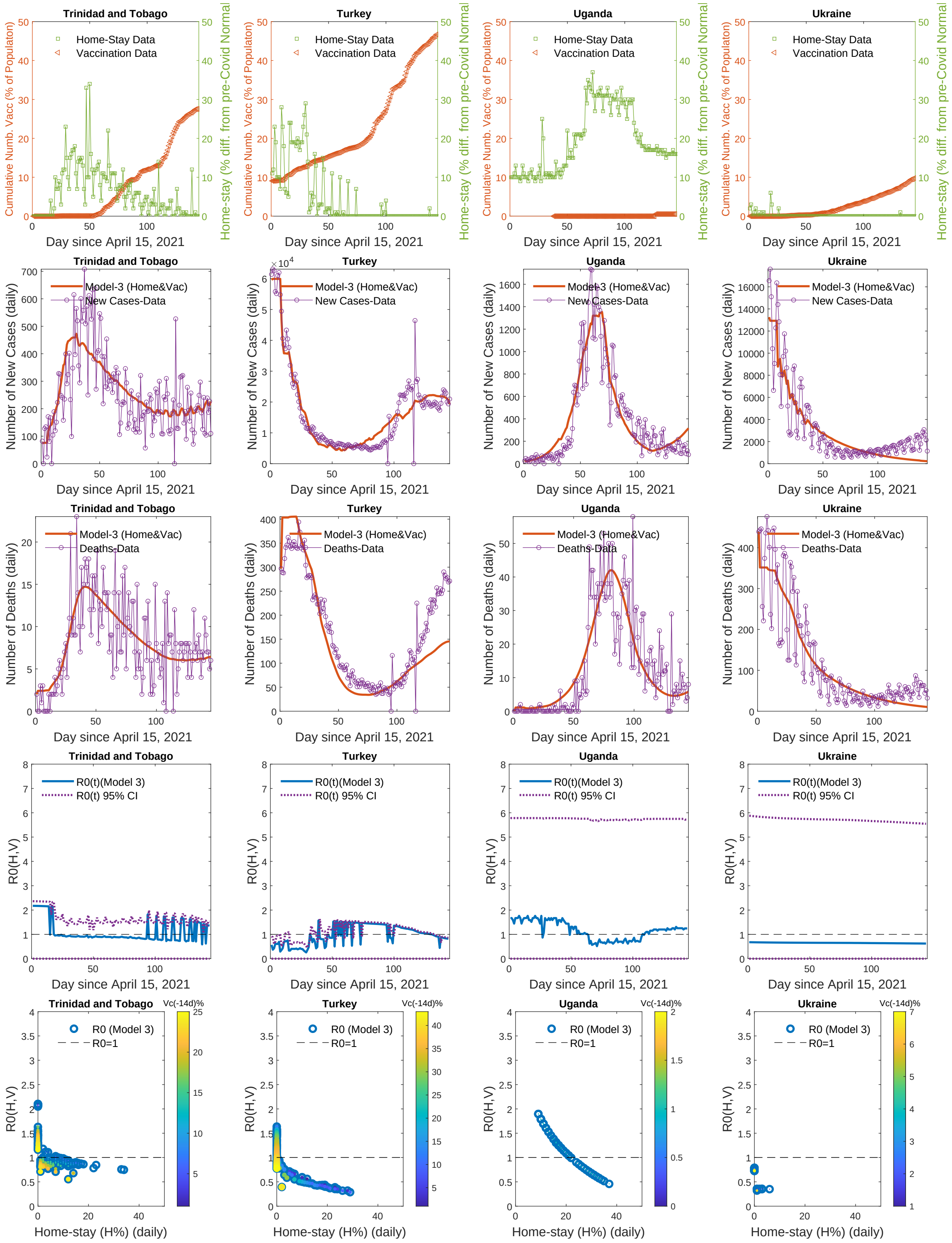
Trinidad and Tobago
Turkey
Uganda
Ukraine
Home-Stay Data
Vaccination Data
Cumulative Numb. Vacc (% of Populaton)
Home-stay (% diff. from pre-Covid Normal
Day since April 15, 2021
Model-3 (Home&Vac)
New Cases-Data
Number of New Cases (daily)
Deaths-Data
Number of Deaths (daily)
R0(t)(Model 3)
R0(t) 95% CI
R0(H,V)
R0 (Model 3)
R0=1
Vc(-14d)%
Home-stay (H%) (daily)

Figure 59

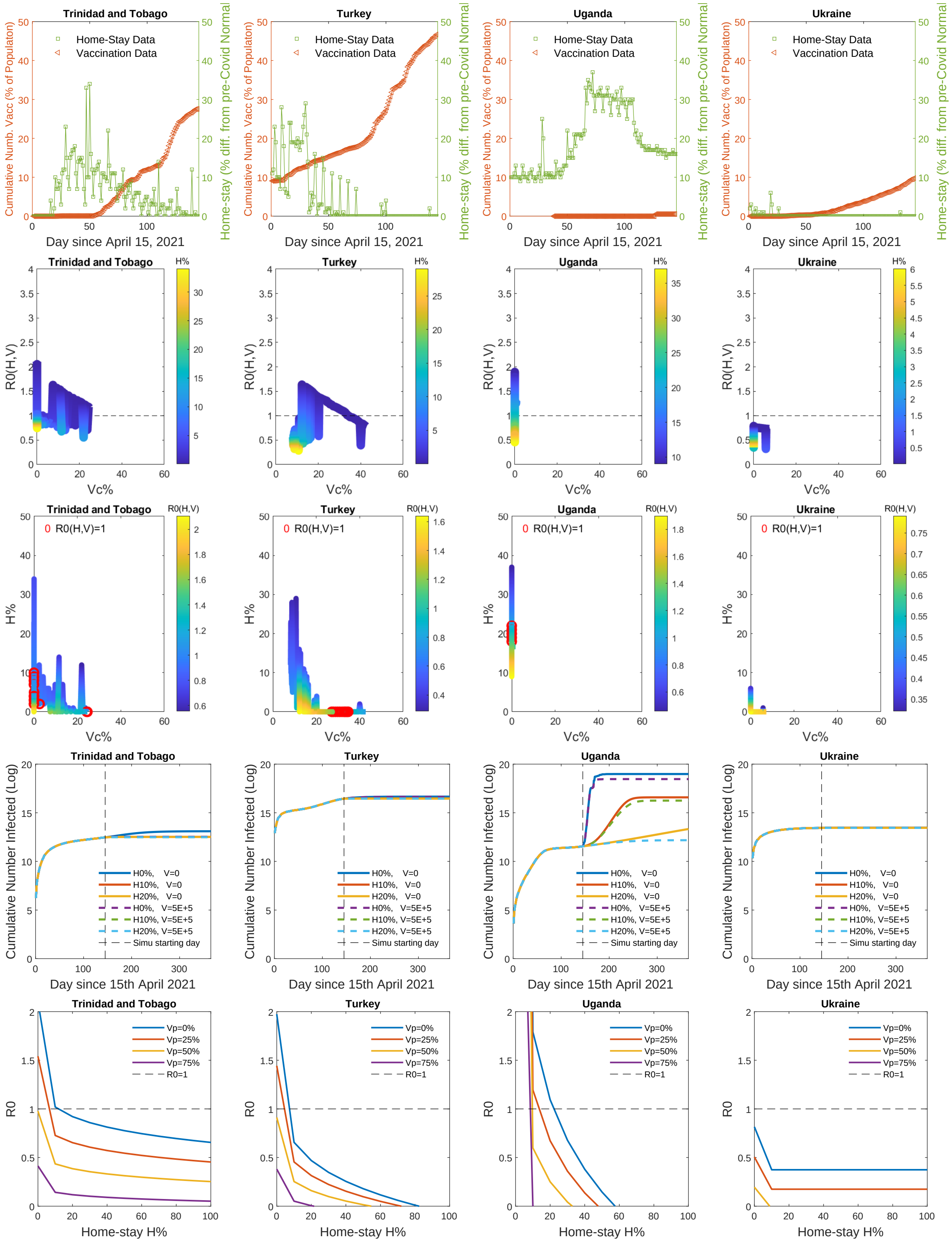
Trinidad and Tobago
Turkey
Uganda
Ukraine
Home-Stay Data
Vaccination Data
Cumulative Numb. Vacc (% of Populaton)
Home-stay (% diff. from pre-Covid Normal
Day since April 15, 2021
R0(H,V)
H%
Vc%
0 R0(H,V)=1
Cumulative Number Infected (Log)
H0%, V=0
H10%, V=0
H20%, V=0
H0%, V=5E+5
H10%, V=5E+5
H20%, V=5E+5
Simu starting day
Day since 15th April 2021
R0
Vp=0%
Vp=25%
Vp=50%
Vp=75%
R0=1
Home-stay H%

Figure 60

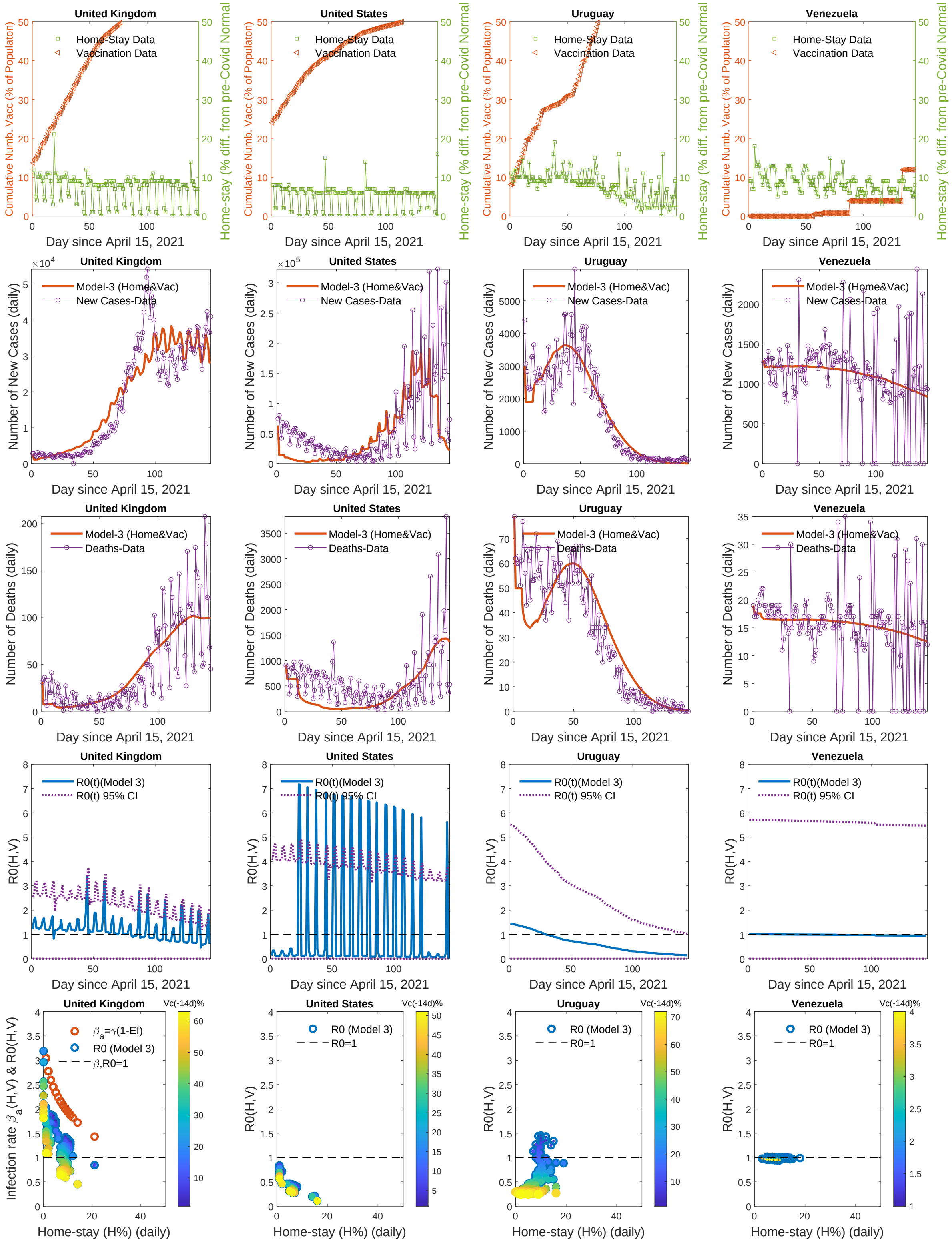
United Kingdom
United States
Uruguay
Venezuela
Home-Stay Data
Vaccination Data
Cumulative Numb. Vacc (% of Populaton)
Home-stay (% diff. from pre-Covid Normal
Day since April 15, 2021
Model-3 (Home&Vac)
New Cases-Data
Number of New Cases (daily)
Deaths-Data
Number of Deaths (daily)
R0(t)(Model 3)
R0(t) 95% CI
R0(H,V)
Vc(-14d)%
R0 (Model 3)
R0=1
Infection rate β_a(H,V) & R0(H,V)
Home-stay (H%) (daily)

Figure 61

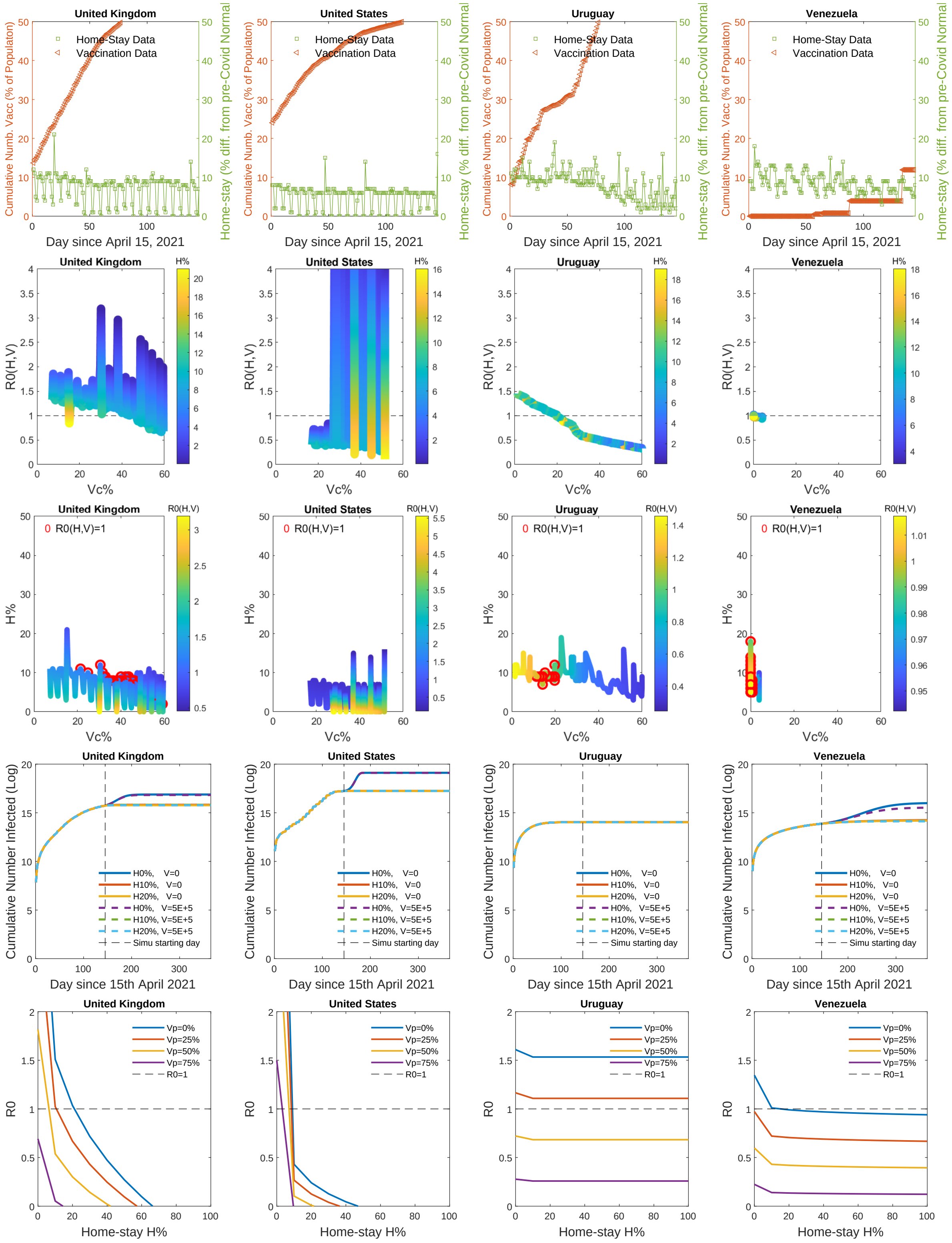
United Kingdom
United States
Uruguay
Venezuela
Home-Stay Data
Vaccination Data
Cumulative Numb. Vacc (% of Populaton)
Home-stay (% diff. from pre-Covid Normal
Day since April 15, 2021
R0(H,V)
H%
Vc%
0 R0(H,V)=1
Cumulative Number Infected (Log)
H0%, V=0
H10%, V=0
H20%, V=0
H0%, V=5E+5
H10%, V=5E+5
H20%, V=5E+5
Simu starting day
Day since 15th April 2021
R0
Vp=0%
Vp=25%
Vp=50%
Vp=75%
R0=1
Home-stay H%

Figure 62

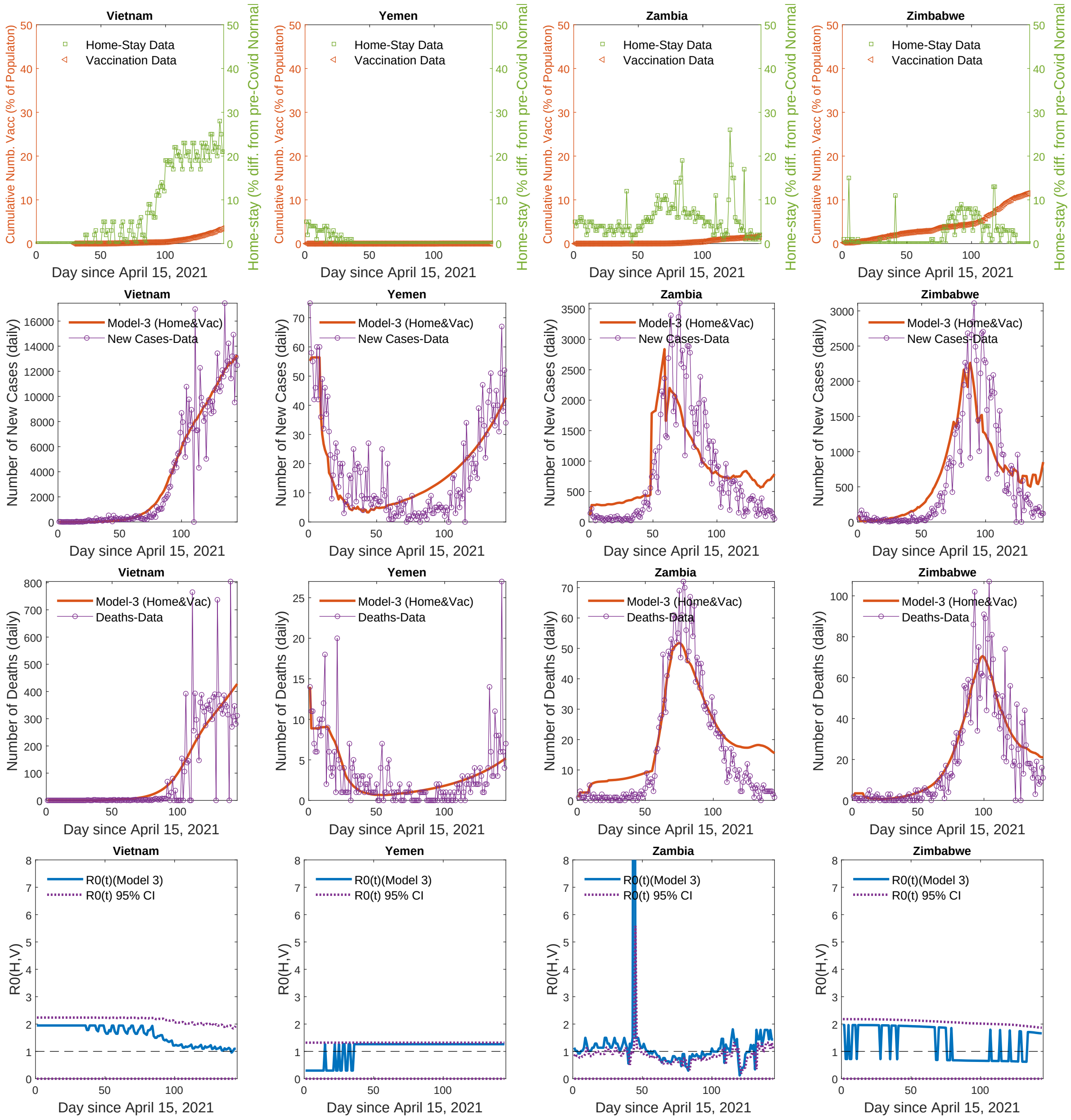
Vietnam
Yemen
Zambia
Zimbabwe
Home-Stay Data
Vaccination Data
Cumulative Numb. Vacc (% of Populaton)
Home-stay (% diff. from pre-Covid Normal
Day since April 15, 2021
Model-3 (Home&Vac)
New Cases-Data
Number of New Cases (daily)
Deaths-Data
Number of Deaths (daily)
R0(t)(Model 3)
R0(t) 95% CI
R0(H,V)

Figure 63

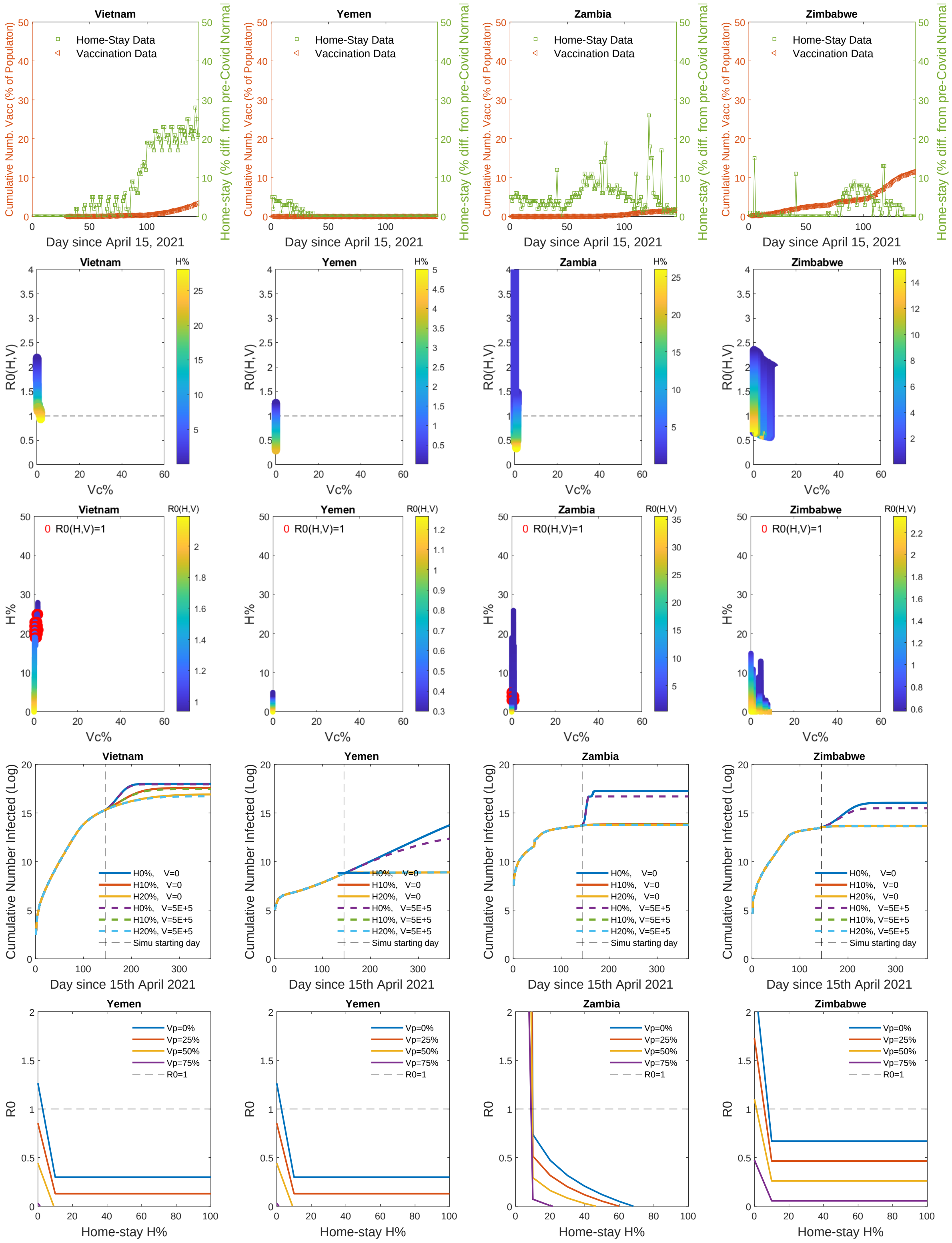
Vietnam
Yemen
Zambia
Zimbabwe
Home-Stay Data
Vaccination Data
Cumulative Numb. Vacc (% of Populaton)
Home-stay (% diff. from pre-Covid Normal
Day since April 15, 2021
R0(H,V)
H%
Vc%
R0(H,V)=1
Cumulative Number Infected (Log)
H0%, V=0
H10%, V=0
H20%, V=0
H0%, V=5E+5
H10%, V=5E+5
H20%, V=5E+5
Simu starting day
Day since 15th April 2021
R0
Vp=0%
Vp=25%
Vp=50%
Vp=75%
R0=1
Home-stay H%

Figure 64

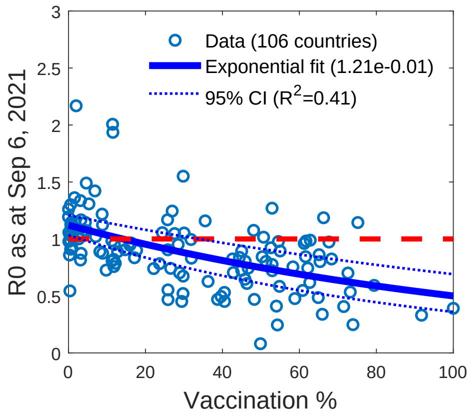

Figure 65: The World data: Current $R0$'s vs. the vaccinated population percentages, $Vc\%$. The current net reproductive rate $R0$, averaged over the last 7 days, of the 106 nations, declines with respect to their present percentages of people vaccinated, $Vc\%$. (The country specific values are given in the Table.1 in the Appendix).
