# Supplementary material for: Dynamical regulations on mobility and vaccinations for controlling COVID-19 spread
Source: Sci Rep. 2022 Mar 3;12:3554. doi: 10.1038/s41598-022-07371-5 (PMC8894369; doi:10.1038/s41598-022-07371-5)
Supplement: Supplementary file 4 — Supplementary Information 3. [file 41598_2022_7371_MOESM4_ESM.pdf]

Dynamical regulations on mobility and vaccinations for controlling COVID-19 spread

Conceptual models of infectious-window and death-window

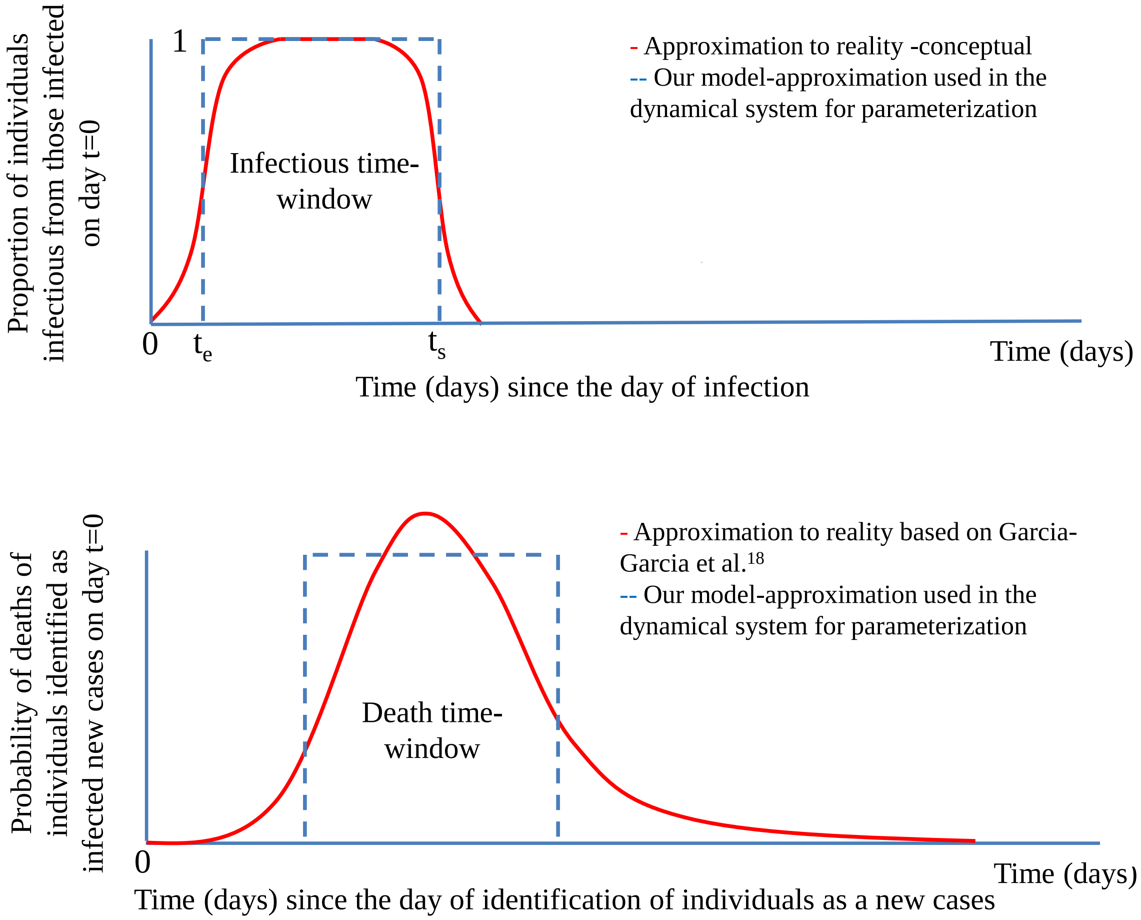

Figure 1: (a) A step-function approximation to infectious-window by assuming, at the begging of the hump, the proportion of individuals infectious at day t from the total infected at day $t = 0$, which is conceptually arising from the cumulative log-normal probability distribution model based on Garcia-Garcia et al.[18], and at the end of the hump, the proportion of individuals still remain infectious at day t from the total infected at day $t = 0$, tapering off the curve over time. The step-function model approximation gives a proportion of 0 or 1 individuals going in and out of being infectious in the infectious time-window. (b) A uniform distribution approximation to the log-normal probability distribution of deaths at days t for individuals identified as a new case on day $t = 0$, or shown illness since day $t = 0$, given by Garcia-Garcia et al.[18]
